# The burden of stomach cancer mortality by county, race, and ethnicity in the USA, 2000–2019: a systematic analysis of health disparities

**DOI:** 10.1016/j.lana.2023.100547

**Published:** 2023-08-04

**Authors:** GBD US Health Disparities Collaborators, Parkes Kendrick, Yekaterina O. Kelly, Mathew M. Baumann, Kelly Compton, Brigette F. Blacker, Farah Daoud, Zhuochen Li, Farah Mouhanna, Hasan Nassereldine, Chris Schmidt, Dillon O. Sylte, Lisa M. Force, Simon I. Hay, Erik J. Rodriquez, George A. Mensah, Anna M. Nápoles, Eliseo J. Pérez-Stable, Christopher J.L. Murray, Ali H. Mokdad, Laura Dwyer-Lindgren

**Keywords:** Stomach cancer mortality, Gastric cancer mortality, Health disparities, Racial-ethnic health disparities, Place-based health disparities, Small area estimates, Local estimates

## Abstract

**Background:**

There are persistent disparities in stomach cancer mortality among racial–ethnic groups in the USA, but the extent to which these patterns vary geographically is not well understood. This analysis estimated age-standardised mortality for five racial–ethnic groups, in 3110 USA counties over 20 years, to describe spatial–temporal variations in stomach cancer mortality and disparities between racial–ethnic groups.

**Methods:**

Redistribution methods for insufficient cause of death codes and validated small area estimation methods were applied to death registration data from the US National Vital Statistics System and population data from the US National Center for Health Statistics to estimate annual stomach cancer mortality rates. Estimates were stratified by county and racial–ethnic group (non-Latino and non-Hispanic [NL] American Indian or Alaska Native [AIAN], NL Asian or Pacific Islander [Asian], NL Black [Black], Latino or Hispanic [Latino], and NL White [White]) from 2000 to 2019. Estimates were corrected for misreporting of racial–ethnic group on death certificates using published misclassification ratios. We masked (ie, did not display) estimates for county and racial–ethnic group combinations with a mean annual population of less than 1000; thus, we report estimates for 3079 (of 3110) counties for the total population, and 474, 667, 1488, 1478, and 3051 counties for the AIAN, Asian, Black, Latino, and White populations, respectively.

**Findings:**

Between 2000 and 2019, national age-standardised stomach cancer mortality was lowest among the White population in every year. Nationally, stomach cancer mortality declined for all racial–ethnic groups across this time period, with the most rapid declines occurring among the Asian (percent decline 48.3% [45.1–51.1]) and Black populations (42.6% [40.2–44.6]). Mortality among the other racial–ethnic groups declined more moderately, decreasing by 36.7% (35.3–38.1), 35.1% (32.2–37.7), and 31.6% (23.9–38.0) among the White, Latino, and AIAN populations, respectively. Similar patterns were observed at the county level, although with wide geographic variation. In 2019, a majority of counties had higher mortality rates among minoritised racial–ethnic populations compared to the White population: 81.1% (377 of 465 counties with unmasked estimates for both racial–ethnic groups) among the AIAN population, 88.2% (1295 of 1469) among the Latino population, 99.4% (663 of 667) among the Asian population, and 99.9% (1484 of 1486) among the Black population. However, the size of these disparities ranged widely across counties, with the largest range from 0.3 to 17.1 among the AIAN population.

**Interpretation:**

Stomach cancer mortality has decreased substantially across populations and geographies in the USA. However, disparities in stomach cancer mortality among racial–ethnic groups are widespread and have persisted over the last two decades. Local-level data are crucial to understanding the scope of this unequal burden among minoritised groups in the USA.

**Funding:**

10.13039/100000025National Institute on Minority Health and 10.13039/100006545Health Disparities; 10.13039/100000050National Heart, Lung, and Blood Institute; 10.13039/100000054National Cancer Institute; 10.13039/100000049National Institute on Aging; 10.13039/100000069National Institute of Arthritis and Musculoskeletal and Skin Diseases; 10.13039/100006085Office of Disease Prevention; and 10.13039/100000118Office of Behavioral and Social Sciences Research, National Institutes of Health (contract #75N94019C00016).

**Research in context**

**Evidence before this study**

Differences in stomach cancer mortality among racial–ethnic groups in the USA have long been recognised at the national level. AIAN, Asian, Black, and Latino populations continue to have higher rates of death due to stomach cancer compared to White populations, despite substantial declines in mortality across all racial–ethnic groups. Additionally, larger declines in absolute versus relative disparities across racial–ethnic groups have been observed. Certain structural inequities such as household crowding are commonly experienced by low-income, rural, and/or racial–ethnic minority populations and are associated with *Helicobacter pylori (H. pylori)* infection, which has been identified as a leading risk factor for stomach cancer. Additionally, other environmental factors—such as a high sodium diet and lack of access to fresh fruits and vegetables—are strongly associated with an increased risk of developing stomach cancer when an individual is infected with *H. pylori*. Other potential risks for stomach cancer, such as commercial tobacco smoking and obesity, are also more common in certain populations, including racial–ethnic minoritised populations and those living in poverty. Additionally, survival following a stomach cancer diagnosis remains especially low compared to other cancer types in the USA.

Stomach cancer mortality rates have also been shown to vary by location, including among states and counties. We searched PubMed from inception to July 8, 2023, using the string “(“Stomach” [All Fields] OR “Gastric” [All Fields]) AND (“Cancer” [All Fields] OR “Neoplasms” [All Fields]) AND (“Mortality” [All Fields] OR “Death”) AND (“County” [All Fields] OR “Subnational”) AND “United States” [All Fields]” for studies examining county-level patterns of stomach cancer mortality by racial–ethnic group in the USA. Several studies have previously examined county-level patterns of stomach cancer mortality, however few have simultaneously stratified by racial–ethnic group, and those that have were all limited in geographic scope.

**Added value of this study**

We estimated age-standardised stomach cancer mortality by year, county, and racial–ethnic group (non-Latino and non-Hispanic American Indian or Alaska Native [AIAN], non-Latino and non-Hispanic Asian or Pacific Islander [Asian], non-Latino and non-Hispanic Black [Black], Latino or Hispanic [Latino], and non-Latino and non-Hispanic White [White]) in the USA from 2000 to 2019. This paper provides the first country-wide time-series analysis of stomach cancer mortality at the USA county level that includes estimates for five racial–ethnic groups. Thus, we present here trends in age-standardised stomach cancer mortality rates and disparities among racial–ethnic groups at a novel level of local granularity and for a longer period of recent history than has previously been studied.

**Implications of all the available evidence**

In 2019, age-standardised stomach cancer mortality was lowest among the White population and highest among the Black population. Between 2000 and 2019, all racial–ethnic groups experienced large declines in mortality, but mortality among minoritised populations remained statistically significantly higher in almost all counties. Mortality declined most rapidly among the Asian and Black populations compared to other racial–ethnic groups, but mortality was so high among these groups in 2000 that disparities compared to the White population persisted across all years. Additionally, progress towards improved stomach cancer outcomes was not equal across counties. Among the AIAN and Latino populations, the national-level disparities compared to the White population remained unchanged over time in both magnitude and direction, and at the county level there was considerable variation in these disparities in 2019. These results highlight important differences in the burden of this disease among racial–ethnic groups and locations. It is crucial to combat systemic racism and poverty which, by various mechanisms, place minoritised populations at increased risk for developing stomach cancer. Furthermore, it may be beneficial to consider *H. pylori* eradication among some populations at higher risk of developing stomach cancer, although critical research is needed in this space. These detailed estimates underscore the importance of understanding unequal progress and persistent disparities.

## Introduction

The American Cancer Society estimates there will be approximately 26,500 new cases and 11,130 deaths from stomach cancer in the United States in 2023.^1^ There are persistent racial and ethnic disparities in stomach cancer mortality rates,^2^, ^3^, ^4^ and stomach cancer is one of the few cancer types (in addition to gallbladder and liver cancer^5^^,^^6^) for which the White population has the lowest mortality rate among racial–ethnic groups.^3^^,^^4^^,^^7^ Although stomach cancer mortality declined by 41.5% in the USA between 1990 and 2017, individuals from minoritised racial–ethnic populations are consistently more likely to be diagnosed with and die from this disease.^8^ Stomach cancer also has one of the lower survival rates across cancers.^9^ If detected at the localised stage, the 5-year relative survival rate in the USA is estimated to be 70%; however, approximately 60% of cases are diagnosed at the regional or distant stage, for which the 5-year relative survival rates are only 31% and 6%, respectively.^9^^,^^10^ The lack of effective screening tests and other preventative interventions for stomach cancer may contribute to these poor outcomes.

Disparities across racial–ethnic groups and the high rate of late-stage diagnoses indicate that progress is needed on prevention of this cancer. *Helicobacter pylori (H. pylori)* infection has been identified as an important risk factor for stomach cancer, and incidence of this bacterial infection is higher in environments with poorer living conditions, such as crowded households.^11^^,^^12^ Additionally, when an individual is infected with *H. pylori*, factors such as a high sodium diet and lack of access to fresh fruits and vegetables are strongly associated with increased risk of developing stomach cancer.^13^ Healthy food options remain less accessible in the USA to lower income individuals, who are also more likely to be from minoritised racial–ethnic populations.^14^ Prevalence of *H. pylori* infection is higher in some countries outside of the USA (such as Japan and Korea), and thus individuals born in these high-prevalence countries and who now live in the USA may be at greater risk of developing stomach cancer.^15^ Additionally, although not definitive, previous studies indicate there may be other important risks involved—such as commercial tobacco smoking and obesity^13^^,^^16^—some of which are more common in certain minoritised racial–ethnic populations, as well as among those living in poverty.^17^, ^18^, ^19^

Understanding disparities in stomach cancer at a local level is important for understanding how to decrease mortality. Previous studies have documented stark racial–ethnic disparities in stomach cancer incidence,^7^^,^^20^ mortality,^2^^,^^4^ and survival in the USA.^2^^,^^10^ However, most studies have focused on disparities and temporal trends at the national level^2^^,^^20^ or in a single location^4^ despite evidence that large differences exist across geography.^21^ Some studies that report across geographies only report on a subset of the USA or include results for only one racial–ethnic population.^7^^,^^10^^,^^21^ To our knowledge, no study has considered how racial–ethnic disparities in stomach cancer mortality have varied simultaneously over time and across counties. To fill these gaps, we estimated age-standardised stomach cancer mortality in the USA annually from 2000 to 2019, stratified by county and racial–ethnic group. This analysis describes how disparities have changed over time, both within and across counties, and they provide the most comprehensive picture to date of stomach cancer mortality in the USA.

## Methods

This analysis uses methods previously developed for estimating cause-specific mortality by county and racial–ethnic group.^22^^,^^23^ We provide a summary of these methods and their application to stomach cancer mortality below.

### Unit of analysis

Estimates were generated by county, sex, and combined race and Latino or Hispanic ethnicity on an annual basis from 2000 to 2019. Race and Latino or Hispanic ethnicity were combined into a single categorisation (“race–ethnicity”) with five mutually-exclusive groups: non-Latino and non-Hispanic American Indian or Alaska Native (AIAN), non-Latino and non-Hispanic Asian or Pacific Islander (Asian), non-Latino and non-Hispanic Black (Black), Latino or Hispanic (Latino), and non-Latino and non-Hispanic White (White). Due to constraints in the underlying data (Supplemental Material p 5), we combined the Asian and Native Hawaiian and Pacific Islander (NHPI) populations for this analysis, however we refer to this combined group as “Asian,” recognising that estimates for this combined group predominantly reflect the experience of the Asian population, which is much larger than the NHPI population (21.7 million non-Hispanic and non-Latino individuals identifying as Asian [either alone or in combination with other racial identities] compared with 1.2 million individuals identifying as NHPI in 2019).^24^

Some county boundaries changed over this period; therefore, we used a previously developed mapping of counties to temporally stable geographic units,^25^ which reduced the number of areas analysed from 3143 to 3110 counties or combined county units (Supplemental Table 3.1 pp 30–31). For simplicity, we refer to these 3110 areas as “counties.”

This study complies with the Guidelines for Accurate and Transparent Health Estimates Reporting (Supplemental Material p 4).^26^ This research received institutional review board approval from the University of Washington. Informed consent was not required because the study used deidentified data and was retrospective.

### Data

We used deidentified death records from the US National Vital Statistics System and population estimates from the US National Center for Health Statistics (NCHS) for this analysis. We tabulated these data by county, age group (0 years, 1–4 years, 5-year age bands from 5–9 to 80–84, 85+ years), sex, racial–ethnic group, and year. The racial–ethnic groups used in this analysis are single-race groups, so for death certificates where the individual was identified as having multiple racial identities, we used the “primary” (or “bridged”) race imputed by NCHS.^27^ We utilised the cause list and hierarchy developed for the Global Burden of Diseases, Injuries, and Risk Factors (GBD) 2021 study, and the associated mapping of ICD-10^28^ codes to GBD causes (Supplemental Material pp 33–47). For stomach cancer, ICD codes C16–C16.9, D00.2, D13.1, and D37.1 were included. We also applied algorithms developed for the GBD study to reassign “garbage codes”—codes assigned as an underlying cause of death that refer to an intermediate or immediate cause of death, are otherwise implausible, or are insufficiently specific—to the likely true underlying causes of death (Supplemental Material pp 5–7).^29^ Across all causes, the percentage of deaths with these codes is similar by racial–ethnic group, ranging from 25.5% (for the Latino population) to 29.4% (for the Black population). All causes in the GBD cause list with at least 10,000 deaths in total over the study period and at least 1000 deaths each among males and females separately were analysed concurrently, however the focus of this paper is on stomach cancer.

To better inform the estimates, especially in county and race–ethnicity combinations with smaller populations, we used data extracted from various sources on income and population density by county, and on post-secondary education, poverty, and birthplace (in the USA vs outside the USA) by county and race–ethnicity as covariates in the statistical model (Supplemental Material pp 7–10, 48–50). Finally, we utilised published estimates of race–ethnicity misclassification ratios, defined as the ratio of deaths among individuals of a particular racial–ethnic group as indicated by self-report to deaths among individuals of that same racial–ethnic group as indicated on death certificates.^30^

### Statistical analysis

We carried out the statistical analysis in three stages. First, we used small area estimation models to estimate stomach cancer mortality rates by county, racial–ethnic group, sex, age, and year, using the racial–ethnic group reported on death certificates. The purpose of these models is to estimate the underlying mortality rate while smoothing out stochastic noise. Models were fit using the Template Model Builder package^31^ in R version 3.6.1,^32^ and 1000 draws of the mortality rate were simulated from the approximated posterior distribution after fitting the models. Further details on model specification, model validation, and model performance are provided in the Supplemental Material (pp 10–22, 64–66). Second, we used race–ethnicity misclassification ratios to adjust draws of the mortality rate derived from the small area model (Supplemental Material pp 22–24). Third, to guarantee consistency among causes and that adjustment for misclassification did not change the overall mortality rate estimated for a given county, we performed a post-hoc calibration using a two-stage iterative proportional fitting algorithm (Supplemental Material pp 24–26).

Final point estimates were derived from the mean of the 1000 draws, and 95% uncertainty intervals (UIs) were derived from their 2.5th and 97.5th percentiles. We generated estimates for males and females combined and at aggregate geographic levels (ie, state and national) by population-weighting the age-specific mortality rates. Age-standardised mortality rates were calculated using the age structure of the USA population as recorded in the 2010 Census as the standard. Disparities in mortality between racial–ethnic groups were measured as both differences (absolute disparities)—calculated by subtracting mortality rates between racial–ethnic groups—and ratios (relative disparities)—calculated by dividing the mortality rates. When comparing any pair of age-standardised mortality estimates, we describe the difference as statistically significant when the posterior probability that the difference is greater than 0 was less than 2.5% or greater than 97.5%, akin to a two-tailed test with α = 0.05. We are using the term “statistically significant” and the threshold of 0.05 outside of the hypothesis testing context to help succinctly reflect the degree of uncertainty in the estimates, as this can vary widely by county and racial–ethnic group.

We masked (ie, did not display) the modelled mortality rate estimates in every year for county and racial–ethnic group combinations that had a mean annual population of less than 1000 because model performance declined notably below this threshold (Supplemental Material pp 18–22). Thus, we report estimates for 3079 (of 3110) counties for the total population, 474 for the AIAN population, 667 for the Asian population, 1488 for the Black population, 1478 for the Latino population, and 3051 for the White population. Over 97% of the population in each racial–ethnic group other than AIAN lived in the counties with unmasked estimates; 82% of the AIAN population lived in counties with unmasked estimates (Supplemental Table 3.5 [p 51]).

### Role of the funding source

Co-authors employed by the NIH contributed to data interpretation and to revising drafts of this report. Otherwise, the funders had no role in study design, data collection, data analysis, or the initial writing of the report.

## Results

Estimates for all counties and racial–ethnic groups are available in an online visualisation tool (https://vizhub.healthdata.org/subnational/usa). Additional tables and figures are available in the Supplemental Material, starting on page 69.

### Temporal changes in mortality and disparities at the national level

**Fig. 1:**
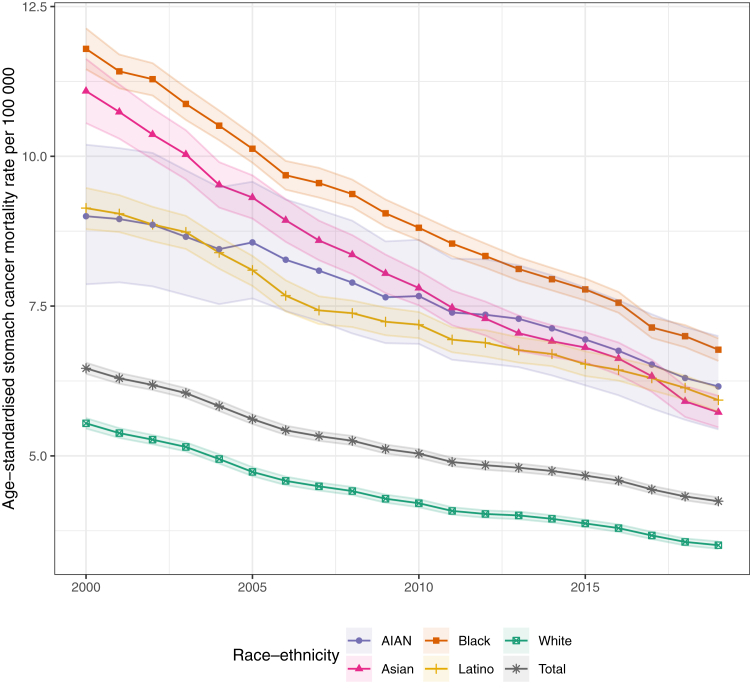
**National estimated age-standardised stomach cancer mortality rates, 2000**–**2019, by year and racial–ethnic group.** Shaded areas indicate 95% uncertainty intervals.

Nationally, age-standardised mortality due to stomach cancer was lowest among the White population and was substantially higher among the other racial–ethnic groups (Fig. 1). In 2019, the mortality rate was 3.5 (3.4–3.6) deaths per 100,000 among the White population, 5.7 (5.5–6.0) among the Asian population, 5.9 (5.7–6.1) among the Latino population, 6.2 (5.4–7.0) among the AIAN population, and 6.8 (6.6–7.0) among the Black population. The size of these differences between racial—ethnic groups differed for males and females separately, although mortality was still substantially lower among the White population compared to other groups (Supplemental Figs. S10 and S13 [pp 80, 83]). Between 2000 and 2019, disparities persisted even as mortality declined for the total population—from 6.5 (6.4–6.6) to 4.2 (4.2–4.3) deaths per 100,000—and for each racial–ethnic group. Mortality rate decreases were largest for the Asian population (48.3% [45.1–51.1], from 11.1 [10.6–11.6] to 5.7 [5.5–6.0] deaths per 100,000), followed by the Black population (42.6% [40.2–44.6], from 11.8 [11.5–12.1] to 6.8 [6.6–7.0] deaths per 100,000), the groups with the highest mortality rates in 2000. In comparison, mortality decreased within the White population by 36.7% (35.3–38.1), from 5.5 (5.5–5.6) to 3.5 (3.4–3.6) deaths per 100,000; in the Latino population by 35.1% (32.2–37.7), from 9.1 (8.8–9.5) to 5.9 (5.7–6.1); and in the AIAN population by 31.6% (23.9–38.0), from 9.0 (7.9–10.2) to 6.2 (5.4–7.0). For all racial–ethnic groups, these decreases occurred fairly evenly across the time series. The largest differences in the rate of change over time were among the White population, for which the mortality rate decreased 24.1% (22.5–25.7) from 2000 to 2010 but only decreased 16.6% (14.7–18.5) from 2010 to 2019.

As stomach cancer mortality declined for every group, disparities compared to the White population decreased, although more so for absolute compared to relative disparities. The largest decrease in absolute disparities was among the Asian population, for which the difference in mortality compared to the White population declined by 3.3 (2.8–3.8) deaths per 100,000 (from 5.5 [5.0–6.1] in 2000 to 2.2 [2.0–2.5] in 2019), while the relative disparities declined from 2.0 (1.9–2.1) to 1.6 (1.6–1.7). Among the Black population, the absolute disparities decreased by 3.0 (2.6–3.3) deaths per 100,000, from 6.3 (5.9–6.6) to 3.3 (3.1–3.4), while the relative disparities decreased from 2.1 (2.1–2.2) to 1.9 (1.9–2.0). These changes were more moderate among the Latino and AIAN populations. Absolute disparities decreased by 1.2 (0.8–1.5) deaths per 100,000—from 3.6 (3.3–3.9) in 2000 to 2.4 (2.2–2.6) in 2019—among the Latino population, and by 0.8 (0.0–1.7) deaths per 100,000—from 3.5 (2.3–4.6) in 2000 to 2.7 (1.9–3.5) in 2019—for the AIAN population. However, because mortality declined at a slightly slower rate among the Latino and AIAN populations compared to the White population, the relative disparities between these groups were fairly stable over time.

### County–level variation in mortality, by racial–ethnic group

**Fig. 2:**
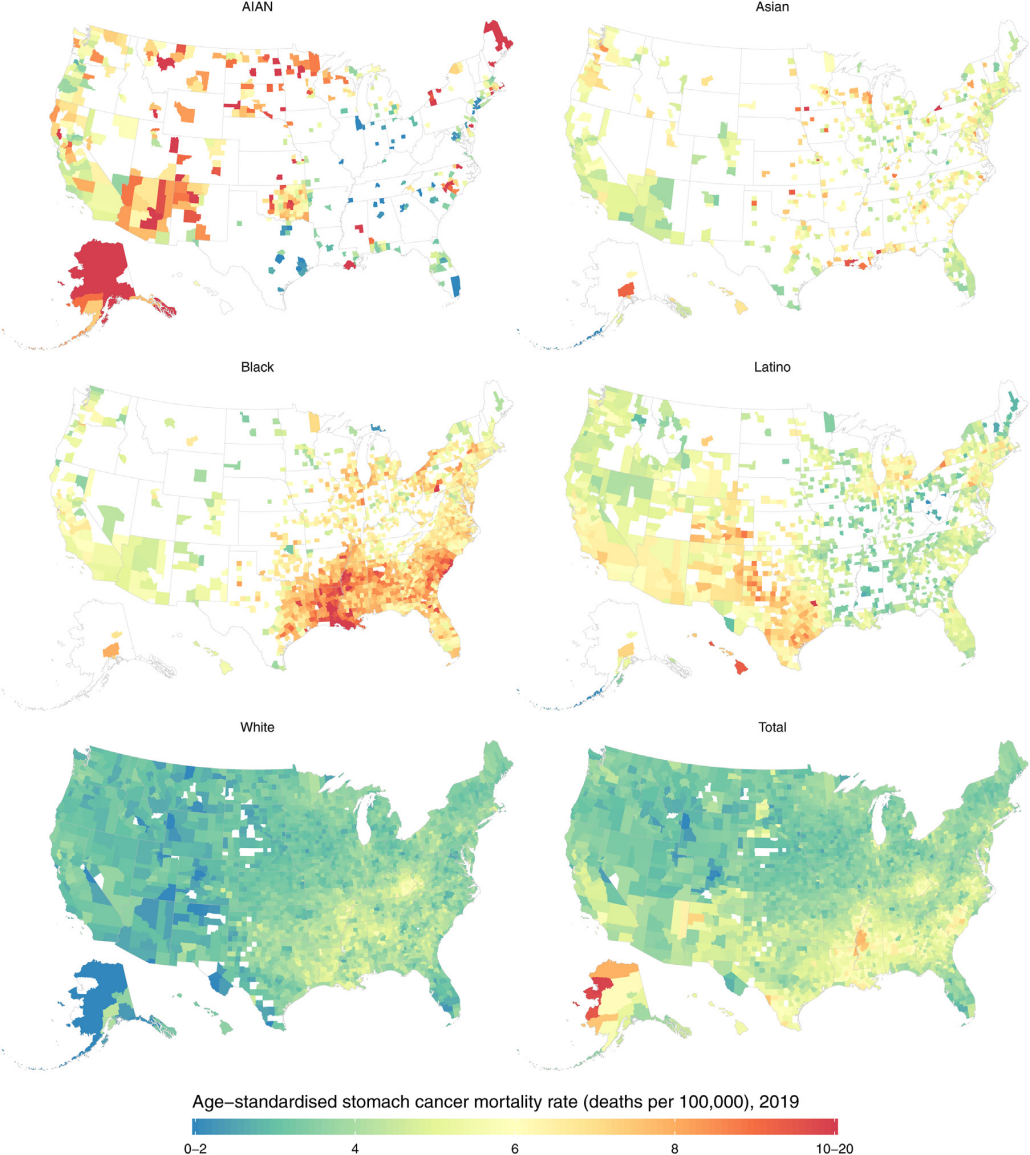
**Estimated age-standardised stomach cancer mortality rates in 2019, by county and racial**–**ethnic group.** The estimated mortality rate within a county by racial**–**ethnic group has been masked if the mean annual population was fewer than 1000 people because model performance declined notably below this threshold.

There was notable variation in mortality due to stomach cancer across counties (Fig. 2), with mortality rates in 2019 for the total population ranging from 1.7 to 9.9 (median 3.9 [interquartile range (IQR) 3.4–4.6]) deaths per 100,000. However, this range widened when additionally stratified by racial–ethnic group: the lowest estimated mortality rate in 2019 was 0.8 while the highest was 20.0 (median 4.6 [IQR 3.6–6.2]) deaths per 100,000. Mortality rates for each racial–ethnic group also varied widely across counties. In 2019, the magnitude of this variation was smallest in absolute terms for the White population, with a median mortality rate of 3.6 and IQR from 3.2 to 4.0 deaths per 100,000. There was more variation among other racial–ethnic groups: the median estimated mortality rates and associated IQRs (ordered from largest to smallest IQR range) were 6.0 (IQR 4.1–7.8) among the AIAN population, 5.1 (IQR 4.4–6.0) among the Latino population, 6.8 (IQR 6.1–7.6) among the Black population, and 5.5 (IQR 4.9–6.3) among the Asian population. Among each racial–ethnic group, the geographic distribution in mortality also differed somewhat for males and females separately. Among the female Latino population, there exists a band of relatively high mortality in the Southwest that is not as prominent for males (Supplementary Figs. S11 and S14 [pp 81, 84]). Among the female Asian population there exists higher relative mortality among many counties in the Midwest and South, a pattern that is less prominent for males (Supplementary Figs. S11 and S14 [pp 81, 84]).

**Fig. 3:**
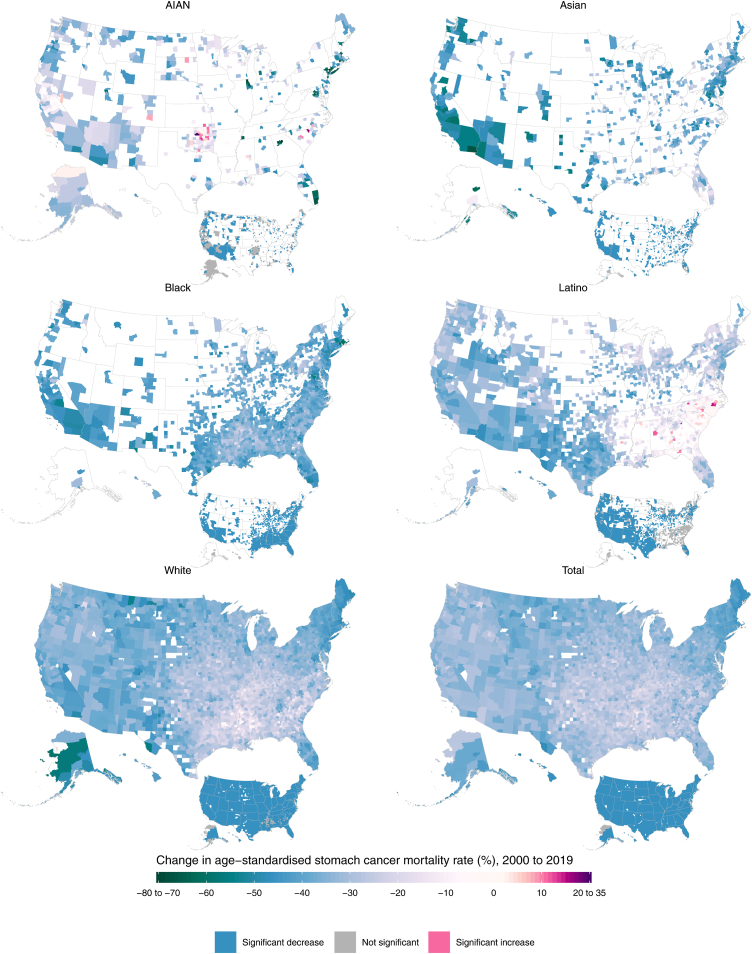
**Percent change in estimated age-standardised stomach cancer mortality rates from 2000 to 2019, by county and racial–ethnic group.** The estimated mortality rate within a county by racial**–**ethnic group has been masked if the mean annual population was fewer than 1000 people because model performance declined notably below this threshold.

Additionally, there was some variation in the temporal changes in mortality across geography, although these patterns were relatively similar across most counties apart from some outliers. Within each racial–ethnic group, over 94% of counties with unmasked estimates had decreases in mortality between 2000 and 2019, with a similar percentage experiencing decreases in both the earlier (2000–2010) and latter (2010–2019) decades (Fig. 3, Supplementary Figs. S5 and S7 [pp 75, 77]). However, the magnitude of these changes varied across counties, and for some racial–ethnic groups the declines were slightly different from 2000 to 2010 compared to 2010 to 2019. Mortality decreased most evenly across the time series among the Asian population; from 2000 to 2010, the median percent decrease was 23.2% (IQR 16.3%–28.1%), and from 2010 to 2019 it was similar (22.9% [IQR 17.6%–26.7%]). The trends across time periods among the Black and AIAN populations were also largely consistent. Among the Black population, the median percent decrease was 23.3% [IQR 19.7%–26.7%] from 2000 to 2010, while it was 20.2% [IQR 17.2%–23.3%] for 2010–2019. Among the AIAN population, from 2000 to 2010 the median percent decrease was 13.5% [IQR 3.7%–24.0%], whereas from 2010 to 2019 it was 17.1% [IQR 10.2%–23.9%]. However, these decreases were less consistent across time periods for the Latino and White populations. Among the Latino population, the median percent decrease was 19.1% [IQR 12.7%–23.6%] for the period from 2000 to 2010, but for 2010–2019 it was 12.7% [IQR 7.0%–17.6%]. Among the White population, the median percent decrease from 2000 to 2010 was 20.8% [IQR 17.0%–24.5%], while it was 13.5% [IQR 10.1%–16.5%] for 2010–2019.

### Intersection of racial–ethnic and county-level inequalities in mortality

**Fig. 4:**
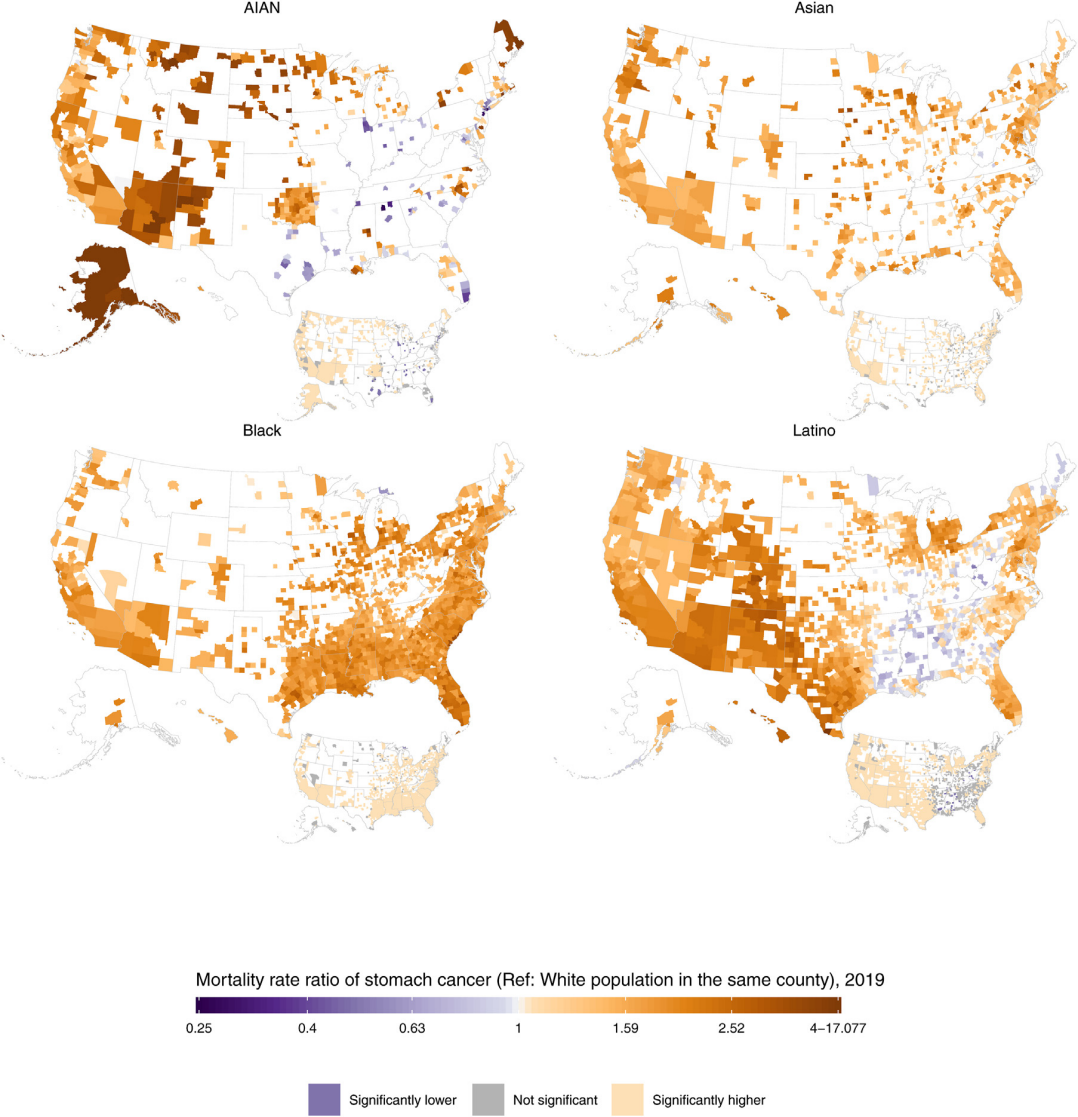
**Ratio of the estimated age-standardised stomach cancer mortality rates among the AIAN, Asian, Black, and Latino populations compared with the White population in 2019, by county.** The estimated mortality rate within a county by racial**–**ethnic group has been masked if the mean annual population was fewer than 1000 people because model performance declined notably below this threshold.

**Fig. 5:**
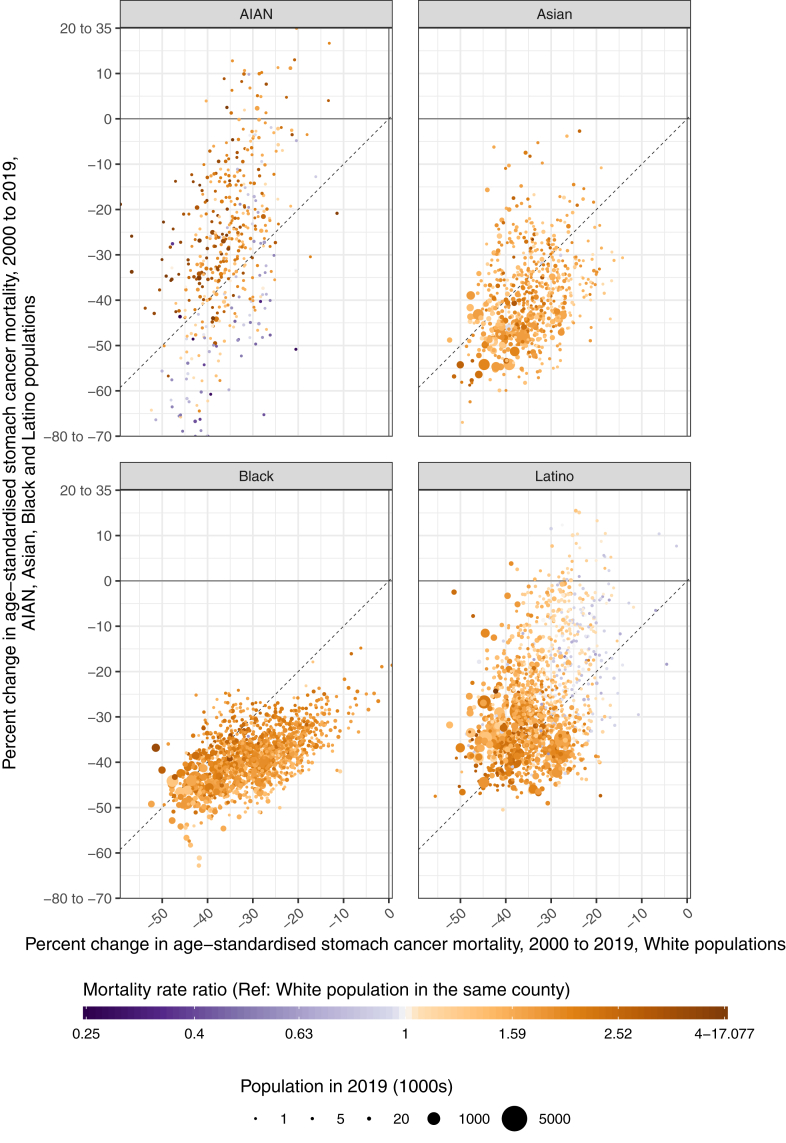
**Percent change in age-standardised stomach cancer mortality from 2000 to 2019 among the AIAN, Asian, Black, and Latino populations compared with the White population, by county.** The estimated mortality rate within a county by racial**–**ethnic group has been masked if the mean annual population was fewer than 1000 people because model performance declined notably below this threshold.

In 2019, the direction of racial–ethnic disparities in stomach cancer mortality was generally consistent across counties (Fig. 4, Fig. 5). Among counties with unmasked estimates, nearly all had higher mortality rates among minoritised racial–ethnic groups compared to the White population: 99.9% (1484 of 1486; 94.4% statistically significant) of counties among the Black population, 99.4% (663 of 667; 83.7% statistically significant) of counties among the Asian population, 88.2% (1295 of 1469; 63.3% statistically significant) of counties among the Latino population, and 81.1% (377 of 465; 63.2% statistically significant) of counties among the AIAN population. There were varying degrees of geographic variation in the size of these disparities, although the IQR tended to be fairly narrow. Within the Latino population, the relative disparities compared to the White population (ratios in mortality rates) ranged from 0.5 to 4.0, with a median of 1.5 and an IQR of 1.2–1.8 among counties with unmasked estimates. The distribution in absolute disparities (differences in mortality rates) had a median of 1.7 deaths per 100,000, ranging from −2.3 to 8.7 but with an IQR of 0.6–2.7. Additionally, among the ten counties with the largest Latino populations, the relative disparities ranged from 1.1 (1.0–1.3) in Miami-Dade County, Florida (corresponding to an absolute disparity of 0.4 [−0.1 to 1.0] deaths per 100,000) to 2.3 (2.0–2.6) in Maricopa County, Arizona (absolute disparity of 3.7 [3.0–4.3] deaths per 100,000). Among the AIAN population, the relative disparities ranged from 0.3 to 17.1 (median 1.8 [IQR 1.2–2.4]) while the absolute disparities ranged from −3.7 to 16.8 (median 2.5 [IQR 0.6–4.7]) deaths per 100,000. There was a wide range in relative disparities compared to the White population among the ten counties with the largest AIAN populations, from 1.5 (1.2–2.1) in Oklahoma County, Oklahoma (absolute disparity of 2.0 [0.6–3.8] deaths per 100,000) to 4.1 (3.1–5.6) in McKinley County, New Mexico (absolute disparity of 6.7 [5.5–8.0] deaths per 100,000).

The geographic distribution in disparities was narrower for the Asian and Black populations. Among the Asian population, the relative disparities ranged from 0.9 to 3.6 (median 1.6 [IQR 1.4–1.8]), while the absolute disparities ranged from −0.5 to 7.7 (median 2.0 [IQR 1.4 to 2.8]) deaths per 100,000. In the ten most populous counties for this racial–ethnic group, the lowest relative disparity was 1.3 (1.1–1.5) in Cook County, Illinois (absolute disparity of 1.1 [0.4–1.9] deaths per 100,000), while the highest was 2.0 (1.8–2.3) in Orange County, California (absolute disparity of 3.0 [2.4–3.6] deaths per 100,000). Among the Black population, the relative disparities ranged from 0.6 to 3.3 (median 1.8 [IQR 1.6–2.0]) while the absolute disparities ranged from −1.5 to 7.8 (median 3.0 [IQR 2.3–3.7]) deaths per 100,000. Among the most populous counties for this population, the relative disparities varied between 1.3 (1.2–1.5) in Kings County, New York (absolute disparity of 1.4 [0.9–2.0] deaths per 100,000) to 2.2 (1.9–2.4) in Dallas County, Texas (absolute disparity of 3.8 [3.2–4.6] deaths per 100,000). Because mortality is so much lower among the White population for both males and females, these results are consistent with what is observed in the estimates for males and females separately (Supplementary Figs. S12 and S15 [pp 82, 85]).

## Discussion

In this study, we estimated trends and disparities in age-standardised stomach cancer mortality by racial–ethnic group and county from 2000 to 2019. In 2000, mortality rates due to stomach cancer were highest among the Black and Asian populations and lowest in the White population at the national level. However, from 2000 to 2019, mortality declined among all racial–ethnic groups, with the most rapid declines occurring among the Asian and Black populations and more moderate declines observed in the AIAN, Latino, and White populations. Despite these improvements, disparities between minoritised racial–ethnic groups and the White population have persisted. Numerous variations on these trends emerged when analysing across racial–ethnic groups at a more localised level. Although even in a perfect world mortality may not be expected to be uniform across all counties or racial–ethnic groups, it is important to consider why it is not, and measuring this variation is crucial for better understanding the shifting landscape of stomach cancer burden across the USA.

The substantial decreases in national-level age-standardised mortality we observed are consistent with previous literature on both mortality and incidence of stomach cancer,^3^^,^^4^^,^^7^^,^^20^ although our mortality estimates tend to be slightly larger than others reported in the literature,^1^ due primarily to the redistribution of deaths with “garbage codes” (Supplementary Material pp 33–47) in this study, which increases the number of deaths attributed to stomach cancer and other causes. Previously published estimates may also have other methodological differences, such as the population standard used for age-standardisation.^33^ Previous studies have primarily attributed decreases in stomach cancer mortality to changes in exposure to risk factors associated with *Helicobacter pylori* (*H. pylori*) infection, as well as improvements in living conditions and diversity of diet.^34^, ^35^, ^36^, ^37^ Declining *H. pylori* prevalence has played an important role, but historical records indicate that declines in stomach cancer burden have coincided with improvements in sanitation and food access even as *H. pylori* prevalence remained high.^36^^,^^38^ Thus, stomach cancer mortality is thought to be associated with a web of often interconnected factors, and as such, the degree of a population's exposure to individual risk factors does not always correlate directly with declines in stomach cancer mortality or with narrowing disparities observed across racial–ethnic groups. For example, commercial tobacco smoking is associated with higher risk of stomach cancer,^39^ but smoking prevalence is generally lower among Asian and Latino populations than in the White population,^17^ whereas the prevalence of *H. pylori* infection is generally higher among minoritised populations.^40^ Additionally, it is not clear why stomach cancer mortality rates are high among the AIAN population in so many different regions. In Alaska, where rates of infection are high, this higher mortality may be at least partially attributable to *H. pylori*,^41^ but further research is needed on the leading drivers in the Southwest and Northern Plains.^42^ Across racial–ethnic groups, there are likely many other important county-level characteristics associated with stomach cancer, and future research could leverage these results to explore the association of stomach cancer with factors such as health insurance coverage or the proximity to a National Cancer Institute designated cancer centre.

Where stomach cancer mortality has less obvious direct causes, these patterns are likely due in part to various environmental factors driven by geographic inequities and systemic racism. For example, household crowding and low educational attainment are associated with *H. pylori* infection and reinfection, and many of these structural barriers are faced at higher rates among minoritised racial–ethnic populations.^11^^,^^12^^,^^43^ The American Community Survey found that the prevalence of household crowding (defined as >1 person per room) in 2019 was only 1.4% among the White population, but 3.6% among the Black population, 7.5% among the Asian population, 8.0% among the AIAN population, 11.4% among the Latino population, and 15% among the NHPI population.^44^ Dietary risk factors for stomach cancer, such as lack of access to fresh fruits and vegetables, present an even greater risk for individuals infected with *H. pylori*.^13^^,^^34^, ^35^, ^36^ Access to these foods may be affected by proximity to grocery stores, which is limited in many rural areas and neighbourhoods with high poverty rates or large minoritised populations.^14^ Additionally, although the literature does not clearly explain how the risk of developing this disease changes upon immigration from a location with high stomach cancer incidence to an area with low stomach cancer incidence, it has been shown that the USA-born children and grandchildren of immigrants will have a lower probability of *H. pylori* infection as well as lower stomach cancer incidence compared to first-generation immigrants.^11^^,^^15^^,^^45^^,^^46^

Advancing clinical research on this disease is also crucial. First, incidence of cardia stomach cancer is increasing in some high-income countries, and an analysis of Surveillance Epidemiology and End Results Program (SEER) data from 2000 to 2014 in the USA found that compared with White adults, incidence rate ratios were lower for cardia stomach cancer versus non-cardia stomach cancer among AIAN, Asian, Black, and Latino adults.^47^ Although the incidence of cardia stomach cancer is still substantially lower than that of non-cardia stomach cancer, the increasing rates among some populations are cause for concern. More research is needed to understand its pathogenesis, which is still largely unknown, although some studies indicate that incidence is associated with obesity and Barrett's oesophagus.^13^ Second, further investigation is needed to better understand factors that impact survival, which has remained persistently low compared to other cancer types in the USA.^10^ At present, differences in survival by racial–ethnic group are not well understood. A 2017 analysis of SEER data reported that adjustment for available patient-level characteristics (including anatomic site and stage of diagnosis) did not fully explain the differences in stomach cancer mortality between the White population and the six largest Asian ethnicities in the USA.^48^

Survival may also be improved through interventions that either prevent the occurrence of stomach cancer or detect it at an early stage. Studies from Asia indicate that eradication of *H. pylori* can lead to lower stomach cancer incidence and mortality.^49^ However, antibiotic resistant strains of *H. pylori* have proliferated over time, and a recent meta-analysis in the USA reported a 31.5% prevalence of *H. pylori* resistance to clarithromycin and 37.6% resistance to levofloxacin among the *H. pylori* samples studied.^50^ Thus, commonly used eradication therapies have become less effective over time, although the small number of studies in other countries exploring the effects of eradication on microbiome health and prevalence of drug-resistant *H. pylori* strains have yielded mixed results.^51^, ^52^, ^53^ Thus, screen-and-treat programs in the USA should be tailored to resistance rates observed in local populations and considered along with factors such as access to *H. pylori* screening sites. However, surveillance data for *H. pylori* are sparse in the USA,^50^ and there have been few studies on the efficacy of a widespread eradication program on improving stomach cancer outcomes.^12^^,^^54^ Additionally, questions around whether *H. pylori* needs to be eradicated among infected, but otherwise healthy, populations remain particularly understudied, though relevant for the USA. Thus, further research is necessary to understand how eradication could improve stomach cancer mortality among high-risk populations in the USA, including better surveillance of both *H. pylori* resistance and current treatment practices, such as what is tracked in an existing registry in Europe.^55^ Alongside scaled-up research efforts, care may be improved if clinicians are able to track the success of *H. pylori* eradication among their patient population and conduct resistance testing as needed to better serve the community.

This analysis is subject to several limitations that have been discussed in previously published work, including errors in the underlying data and covariates, the degree of smoothing in the model, and racial–ethnic misclassification corrections.^22^ Particularly pertinent to this analysis, some previous studies have indicated that racial–ethnic misclassification varies by cause of death; however, due to limited availability of the data necessary for producing cause-specific adjustments, this study uses misclassification ratios designed for all-cause mortality.^56^^,^^57^ Any errors in these misclassification corrections will impact the estimated mortality rates as well as the apparent size of the disparities. Second, although the racial–ethnic stratification presented in this analysis is more extensive than in previous studies, we do not include separate results for individuals who identify as multiracial (despite a growing population in the USA),^58^ and there may still be important within-group heterogeneity that is not reflected in our estimates. For instance, due to data constraints, we produced estimates for a combined Asian and NHPI group; however, numerous studies have shown that stomach cancer incidence, survival, and mortality vary within the Asian and NHPI population, with highest burden among Korean populations, followed by Japanese, Vietnamese, Chinese, and Native Hawaiian.^4^^,^^59^^,^^60^ Similar variation has been found within the Latino population, with the highest burden found among Mexican, followed by Puerto Rican, populations.^61^^,^^62^ Finally, as with other cross-sectional studies, these estimates represent a series of time-specific snapshots of stomach cancer mortality and should be interpreted cautiously, especially for counties with highly dynamic populations, such as those with large correctional facilities, military bases, universities, or a substantial amount of migration in or out of those geographic areas.

For the first time, this study provides estimates of stomach cancer mortality by racial–ethnic group at the county level over time, thus highlighting the large geographic variation in mortality both within and across racial–ethnic groups. By modelling spatiotemporally and by racial–ethnic group, these results provide novel, granular insights into how disparities have changed. The large degree of variation across these dimensions—even within the most populous counties for each racial–ethnic group—highlights the importance of tracking local-level data to support the elimination of persistent racial–ethnic disparities in stomach cancer mortality in the USA. Efforts should focus on additional research on clinical prevention strategies, as well as upstream causes of disparities, such as access to healthy food and opportunities for socioeconomic stability. These are crucial steps towards continuing the decreasing trend in stomach cancer mortality and its associated disparities, to better ensure that all people in the USA—regardless of racial–ethnic identity or place of residence—can live healthy, happy, and full lives.

## Contributors

L Dwyer-Lindgren, A H Mokdad, and C J L Murray were responsible for the study concept and design. Y O Kelly and M M Baumann extracted and processed the data inputs. P Kendrick, D O Sylte, and C Schmidt wrote the computer code and designed and carried out the statistical analyses with input from L Dwyer-Lindgren. P Kendrick, Y O Kelly, and M M Baumann prepared the tables and figures. P Kendrick wrote the first draft of the paper. All authors contributed to writing subsequent versions of the manuscript, critically reviewed the methods and results, and approved the final version of the manuscript. L Dwyer-Lindgren, A H Mokdad, B F Blacker, and F Daoud managed the project. All authors provided intellectual inputs into aspects of this study. Y O Kelly, M M Baumann, P Kendrick, and L Dwyer-Lindgren accessed and verified the data. L Dwyer-Lindgren, A H Mokdad, and C J L Murray had final responsibility for the decision to submit for publication.

## Data sharing statement

Estimates of stomach cancer mortality by county, racial–ethnic group, year, age, and sex are available for download from the Global Health Data Exchange (https://ghdx.healthdata.org/record/ihme-data/united-states-stomach-cancer-mortality-by-county-race-ethnicity-2000-2019) and via a user-friendly data visualisation: https://vizhub.healthdata.org/subnational/usa. Information about the underlying data sources is available in the Supplementary Material (pp 32, 48–49). The code used for this analysis is available on GitHub: https://github.com/ihmeuw/USHD.

## Editor note

The Lancet Group takes a neutral position with respect to territorial claims in published maps and institutional affiliations.

## Declaration of interests

K Compton reports support for the present manuscript from the Bill & Melinda Gates Foundation (grant #OPP1152504). L M Force reports grants or contracts from the Bill & Melinda Gates Foundation, Conquer Cancer Foundation, St. Jude Children's Research Hospital, St. Baldrick's Foundation, and the NIH Loan Repayment Program; and leadership or fiduciary role in other board, society, committee or advocacy group, paid or unpaid, with the Lancet Oncology International Advisory Board, all outside the submitted work.
