# Supplementary material for: The burden of stomach cancer mortality by county, race, and ethnicity in the USA, 2000–2019: a systematic analysis of health disparities
Source: Lancet Reg Health Am. 2023 Aug 4;24:100547. doi: 10.1016/j.lana.2023.100547 (PMC10435837; doi:10.1016/j.lana.2023.100547)
Supplement: Supplementary Figs. S1–S15 and Tables S1 and S2 [file mmc1.docx]

Supplemental Material: The burden of stomach cancer mortality by county, race, and ethnicity in the USA, 2000–2019: a systematic analysis of health disparities

Contents

[1 GATHER Checklist 4](#_Toc137560725)

[2 Supplemental Methods 5](#_Toc137560726)

[2.1 Deaths and population data processing 5](#_Toc137560727)

[2.2 Cause list, misdiagnosis correction, and garbage code redistribution 6](#_Toc137560728)

[2.3 Covariate imputation and smoothing 7](#_Toc137560729)

[2.4 Small area model specification 10](#_Toc137560730)

[County and racial–ethnic group model 10](#_Toc137560731)

[County-level model 16](#_Toc137560732)

[Hyperprior sensitivity analysis 18](#_Toc137560733)

[2.5 Small area model validation 18](#_Toc137560734)

[Approach 18](#_Toc137560735)

[Results 21](#_Toc137560736)

[2.6 Derivation of misclassification ratios by county, age, sex, and racial–ethnic group 22](#_Toc137560737)

[Extraction 22](#_Toc137560738)

[Combination 23](#_Toc137560739)

[Mortality rate adjustment 24](#_Toc137560740)

[2.7 Calibration 24](#_Toc137560741)

[Impact of misclassification adjustment and calibration 26](#_Toc137560742)

[2.8 References 28](#_Toc137560743)

[3 Supplemental Methods Tables 30](#_Toc137560744)

[3.1 Counties combined to create historically stable units of analysis 30](#_Toc137560745)

[3.2 Deaths and population data sources 32](#_Toc137560746)

[3.3 GBD cause list and associated ICD-10 codes 33](#_Toc137560747)

[3.4 Covariate data sources 48](#_Toc137560748)

[3.5 Population mask 51](#_Toc137560749)

[3.6 Hyperparameter posterior means and standard errors for stomach cancer model 53](#_Toc137560750)

[3.7 County and racial–ethnic groups in the all-cause mortality validation set 56](#_Toc137560751)

[3.8 All-cause mortality model validation results 61](#_Toc137560752)

[4 Supplemental Methods Figures 63](#_Toc137560753)

[4.1 Analysis flow chart 63](#_Toc137560754)

[4.2 Hyperprior sensitivity analysis results for selected Level 2 causes of death 64](#_Toc137560755)

[4.3 All-cause mortality model validation results 66](#_Toc137560756)

[4.4 Impact of misclassification adjustment on national age-standardised all-cause mortality 67](#_Toc137560757)

[4.5 Impact of misclassification adjustment on county age-standardised all-cause mortality estimates 68](#_Toc137560758)

[5 Supplemental Results Tables 69](#_Toc137560759)

[eTable 1: Change in absolute disparities in stomach cancer mortality among the AIAN, Asian, Black, and Latino populations compared with the White population in the same county, 2000 to 2019 69](#_Toc137560760)

[eTable 2: Change in relative disparities in stomach cancer mortality among the AIAN, Asian, Black, and Latino populations compared with the White population in the same county, 2000 to 2019 70](#_Toc137560761)

[6 Supplemental Results Figures 71](#_Toc137560762)

[eFigure 1: Age-standardised mortality rate, stomach cancer, 2000 71](#_Toc137560763)

[eFigure 2: Age-standardised mortality rate ratio compared to the White population in the same county, stomach cancer, 2000 72](#_Toc137560764)

[eFigure 3: Age-standardised absolute difference in mortality compared to the White population in the same county, stomach cancer, 2000 73](#_Toc137560765)

[eFigure 4: Age-standardised absolute difference in mortality compared to the White population in the same county, stomach cancer, 2019 74](#_Toc137560766)

[eFigure 5: Percent change in age-standardised mortality rate, stomach cancer, 2000-2010 75](#_Toc137560767)

[eFigure 6: Absolute change in age-standardised mortality rate, stomach cancer, 2000-2010 76](#_Toc137560768)

[eFigure 7: Percent change in age-standardised mortality rate, stomach cancer, 2010-2019 77](#_Toc137560769)

[eFigure 8: Absolute change in age-standardised mortality rate, stomach cancer, 2010-2019 78](#_Toc137560770)

[eFigure 9: Absolute change in age-standardised mortality rate, stomach cancer, 2000-2019 79](#_Toc137560771)

[eFigure 10: National-level age-standardised mortality rate, stomach cancer, 2019, Males 80](#_Toc137560772)

[eFigure 11: Age-standardised mortality rate, stomach cancer, 2019, Males 81](#_Toc137560773)

[eFigure 12: Age-standardised mortality rate ratio compared to the White population in the same county, stomach cancer, 2019, Males 82](#_Toc137560774)

[eFigure 13: National-level age-standardised mortality rate, stomach cancer, 2019, Females 83](#_Toc137560775)

[eFigure 14: Age-standardised mortality rate, stomach cancer, 2019, Females 84](#_Toc137560776)

[eFigure 15: Age-standardised mortality rate ratio compared to the White population in the same county, stomach cancer, 2019, Females 85](#_Toc137560777)

# 1 GATHER Checklist

| Item # | Checklist item | Description of Compliance |
| --- | --- | --- |
| Objectives and funding | | |
| 1 | Define the indicator(s), populations (including age, sex, and geographic entities), and time period(s) for which estimates were made. | Abstract, Introduction, Methods sections |
| 2 | List the funding sources for the work. | Article Information |
| Data Inputs | | |
| ***For all data inputs from multiple sources that are synthesised as part of the study:*** | | |
| 3 | Describe how the data were identified and how the data were accessed. | Methods section |
| 4 | Specify the inclusion and exclusion criteria. Identify all ad-hoc exclusions. | Methods section |
| 5 | Provide information on all included data sources and their main characteristics. For each data source used, report reference information or contact name/institution, population represented, data collection method, year(s) of data collection, sex and age range, diagnostic criteria or measurement method, and sample size, as relevant. | Methods section, Supplemental Methods, Supplemental Material sections 3.2–3.3 |
| 6 | Identify and describe any categories of input data that have potentially important biases (e.g., based on characteristics listed in item 5). | Methods section |
| ***For data inputs that contribute to the analysis but were not synthesised as part of the study:*** | | |
| 7 | Describe and give sources for any other data inputs. | N/A |
| ***For all data inputs:*** | | |
| 8 | Provide all data inputs in a file format from which data can be efficiently extracted (e.g., a spreadsheet rather than a PDF), including all relevant meta-data listed in item 5. For any data inputs that cannot be shared because of ethical or legal reasons, such as third-party ownership, provide a contact name or the name of the institution that retains the right to the data. | https://ghdx.healthdata.org/record/ihme-data/united-states-stomach-cancer-mortality-by-county-race-ethnicity-2000-2019 |
| Data analysis | | |
| 9 | Provide a conceptual overview of the data analysis method. A diagram may be helpful. | Methods section, Supplemental Material section 4.1 |
| 10 | Provide a detailed description of all steps of the analysis, including mathematical formulae. This description should cover, as relevant, data cleaning, data pre-processing, data adjustments and weighting of data sources, and mathematical or statistical model(s). | Methods section, Supplemental Material section 2 |
| 11 | Describe how candidate models were evaluated and how the final model(s) were selected. | Supplementary Material section 2 |
| 12 | Provide the results of an evaluation of model performance, if done, as well as the results of any relevant sensitivity analysis. | Supplemental Material section 2 |
| 13 | Describe methods for calculating uncertainty of the estimates. State which sources of uncertainty were, and were not, accounted for in the uncertainty analysis. | Methods section, Supplemental Material section 2 |
| 14 | State how analytic or statistical source code used to generate estimates can be accessed. | https://github.com/ihmeuw/USHD |
| Results and Discussion | | |
| 15 | Provide published estimates in a file format from which data can be efficiently extracted. | https://ghdx.healthdata.org/record/ihme-data/united-states-stomach-cancer-mortality-by-county-race-ethnicity-2000-2019 |
| 16 | Report a quantitative measure of the uncertainty of the estimates (e.g. uncertainty intervals). | Results section, https://ghdx.healthdata.org/record/ihme-data/united-states-stomach-cancer-mortality-by-county-race-ethnicity-2000-2019 |
| 17 | Interpret results in light of existing evidence. If updating a previous set of estimates, describe the reasons for changes in estimates. | Introduction, Discussion sections |
| 18 | Discuss limitations of the estimates. Include a discussion of any modelling assumptions or data limitations that affect interpretation of the estimates. | Discussion section |

Checklist template obtained from: <http://gather-statement.org/>

# 2 Supplemental Methods

The methods used for this analysis and described below are an extension of methods previously developed for estimating all-cause mortality and life expectancy by county and racial–ethnic group in the USA.^1^

## 2.1 Deaths and population data processing

We used de-identified death records from the National Vital Statistics System (NVSS) and population estimates from the National Center for Health Statistics (NCHS) for the years 2000–2019 for this analysis (section 3.2). This analysis uses the racial-ethnic groups as specified in the standards for federal data collection on race and ethnicity issued by the Office of Management and Budget (OMB) in 1977.^2^ These standards were updated in 1997 to require that federal data collection systems provide separate Asian and Native Hawaiian and other Pacific Islander (NHPI) groups, and to allow individuals to identify as two or more races.^3^ However, these changes were not implemented on death certificates in all states until 2017,^4^ and estimates of misclassification of racial–ethnic group on death certificates are currently only available using the 1977 categorisation.^5^ Additionally, it is not possible to entirely disaggregate the Asian and NHPI populations in death certificate data prior to 2011, owing to the use of a combined Other Asian and Pacific Islander residual category for individuals who are Asian or NHPI but not one of the specific nationalities listed on death certificates (eg, Chinese or Hawaiian).

Deaths were tabulated by county, age group (0, 1–4, 5–9, …, 80–84, 85+ years of age), sex, racial–ethnic group, year, and cause. In cases where age was missing (0.0177% of deaths), we calculated the proportion of deaths within each age group by state, year, sex, and racial–ethnic group among those entries with age information and then reapportioned deaths without age information using these proportions. This approach effectively assumes that age is missing at random. We are unable to verify this assumption; however, we believe it’s unlikely that violations of this assumption would substantively impact the results of our analysis, given the rarity of missing age in the deaths data. In cases where death records were missing information on race or where race was coded as “other” (0.63%), NCHS imputed a value. In cases where multiple race information was collected and decedents were identified as two or more races (0.29%), NVSS included an imputed or “bridged” race value that corresponds to the predicted “primary” race for each decedent.^4^ We used these imputed and bridged values for the present analysis. NCHS does not impute missing Latino ethnicity (0.29%); for the purposes of this analysis, deaths among individuals with unknown Latino ethnicity were redistributed in proportion to the size of the Latino and non-Latino populations in the same county, race, age, sex, and time period. To ensure stable ratios for this redistribution, we pooled data over time as needed to achieve a minimum population of 50. This threshold of 50 is somewhat arbitrary, but was selected in an attempt to balance the need for stable ratios with the desire to have ratios that reflect the distribution of the population within as specific a time-range as possible.

## 2.2 Cause list, misdiagnosis correction, and garbage code redistribution

We used the cause list developed for the Global Burden of Diseases, Injuries, and Risk Factors (GBD) 2021 study for this analysis. The GBD cause list is arranged hierarchically in four levels; within each level, the cause list is designed such that each death is assigned to exactly one cause. Level 1 includes three broad groups of causes (communicable, maternal, neonatal, and nutritional diseases; non-communicable diseases; and injuries), Level 2 includes 21 more detailed cause groupings, Level 3 includes 139 specific causes, and Level 4 is the most detailed with 226 causes (including 102 Level 3 causes that are not further disaggregated at Level 4). We utilised the map developed by the GBD study to translate ICD-10 codes into GBD causes (section 3.3).

Previous studies have identified two issues that require adjustments to the underlying cause of death codes recorded in death certificate data. First, trends in mortality due to Alzheimer's disease and other dementias, Parkinson’s disease, and atrial fibrillation and flutter based on death certificate data are often inconsistent with trends in prevalence and case-fatality from other data sources. This inconsistency is thought be the result of changes over time and differences by location in coding practices related to these causes. Second, many deaths are observed to have been assigned an underlying cause of death code that instead refers to an intermediate or immediate cause of death (eg, cardiopulmonary arrest) or that is insufficiently specific (eg, malignant neoplasm of other and ill-defined sites); these codes are referred to as “garbage codes.”^6^ Failure to address garbage codes may result in erroneous geographic and temporal patterns as the prevalence of garbage codes varies over time and by location, and may also lead to incorrect relative rankings among causes as the likelihood that a death is assigned a garbage code varies by true underlying cause of death.

We used an existing suite of methods developed by the GBD study to address these two issues.^7^ In the first case, this suite of methods operates by reassigning some proportion of deaths originally assigned to other causes to Alzheimer’s disease and other dementias, Parkinson’s disease, or atrial fibrillation and flutter to address under-coding; or by reassigning some proportion of deaths originally assigned to Alzheimer’s disease and other dementias to other causes to address over-coding (Parkinson’s disease and atrial fibrillation and flutter are assumed to only ever be under-coded). In the second case, this suite of methods operates by reassigning deaths originally assigned to garbage codes to likely true underlying causes of death. In both cases, the key details are determining the “target” causes for any deaths being reassigned and determining the proportion of these deaths to be reassigned to each specific target cause. A variety of methods are used to determine the target causes and the redistribution proportions, which are described elsewhere.^7^ For the purposes of this analysis, the same pairings of source and target codes and the associated redistribution proportions derived for the GBD study at the national or state level in the USA were applied to deaths recorded for each racial–ethnic group and for each county within a given state. This process ensures consistency in the data used for this analysis and for the GBD study, and does not alter the total number of deaths observed in any county, racial–ethnic group, age, sex, or year combination, but only effects the allocation of these deaths among different causes.

## 2.3 Covariate imputation and smoothing

The small area estimation models used in the present study to estimate mortality rates leverages observed relationships between the level of mortality and sociodemographic and socioeconomic factors (covariates) to improve estimates where populations are small and the observed number of deaths is an imprecise indicator of the underlying mortality rate. Among the five covariates used in this analysis, educational attainment (bachelor’s degree or higher), poverty rate, and proportion foreign-born were stratified by both county and race–ethnicity, whereas median household income and population density were stratified only by county and represent estimates for the total population in that county (section 3.4). The underlying data for the covariates stratified by both county and racial–ethnic group contained missing values and displayed instability and low precision for some strata with small populations. As covariate estimates are required for all location, year, and racial–ethnic group combinations to derive mortality predictions, small area imputation models were developed to derive smoothed covariate estimates in all years, counties, and racial–ethnic groups prior to including these covariates in the mortality models. Covariates that were not stratified by racial–ethnic group in addition to county were incorporated in the mortality models in their original (non-imputed) forms due to their larger sample sizes and complete spatial and temporal coverage.

Covariate data were derived primarily from the American Community Survey (ACS) and decennial population census. Racial classifications in the tabulated ACS and census data include separate groups for Asian and for Native Hawaiians and other Pacific Islander (NHPI) populations; covariate data for these groups were combined into a single group for consistency with the mortality models. Covariate data were also combined for merged counties to derive a stable location set matching that used in the mortality models (section 3.1). The ACS provides estimates of uncertainty as Margins of Error (MOE) at a confidence level of 90%, rather than providing variance estimates themselves. Per guidance from the Census Bureau,^8^ variance was calculated as:

$$\mathrm{Var}\left( X_{i} \right)=\left( \frac{\mathrm{MOE}\left( X_{i} \right)}{1.645} \right)^{2}$$

where $X_{i}$ is the reported estimate for group $i$. Also following Census guidance, variances for merged geographic and demographic entities were calculated as the sum of the variances over component groups, assuming independence:

$$\mathrm{Var}\left( X_{1}+X_{2} \right)=\mathrm{Var}\left( X_{1} \right)+\mathrm{Var}(X_{2})$$

As the ACS and decennial census data were derived from population samples but are reported as values scaled to total population sizes, their effective sample sizes were estimated to appropriately scale sampling variance in the imputation models, using a three-pronged strategy. The long-form decennial census questionnaires in 1990 and 2000, from which the education, poverty, and foreign-born data were derived for those years, were collected from a 20% and 17% population sample, respectively^9^. Effective sample sizes were therefore assumed to be 20% or 17% of the total population for each county, year, and racial–ethnic group combination in 1990 and 2000, respectively.

Effective sample sizes for ACS data, for which variances were calculated as above, were estimated by the relationship between effective sample size, $\hat{n}_{eff}$, estimates of the population proportion, $\hat{p}$, and its variance, $Var(\hat{p})$^10^:

$$\hat{n}_{eff}=\frac{\hat{p}(1-\hat{p})}{\mathrm{Var}(\hat{p})}$$

As observations with observed proportions of 0·0 or 1·0 yield effective sample sizes of 0·0, the reported population fractions ($\hat{p}$) for these county, year, and race–ethnicity combinations were transformed via an empirical logit transformation^11^ and then inverse-transformed by use of the standard inverse logit function before calculating the corresponding effective sample sizes:

$$\hat{p}_{emp}=\mathrm{logit}^{-1}\left( \log\left( \frac{\hat{p}+\frac{\varepsilon}{2}}{1-\hat{p}+\frac{\varepsilon}{2}} \right) \right)$$

where $\mathrm{logit}^{-1}$ is the standard inverse logit function and $\varepsilon$ is defined as the smallest non-zero proportion in the data set. Effective sample sizes were calculated by use of these transformed proportions and the original reported $Var(\hat{p})$. Counts of individuals with the modelled outcome (attainment of a bachelor’s degree or higher, living below the poverty line, or foreign-born) were then calculated for each row by multiplying their original reported proportions and estimated effective sample sizes.

Bayesian imputation models were fit in R-INLA^12^ v.20.09.25 in R v3.5.1^13^ by use of binomial likelihood models, and explicitly borrow strength over space, time, and racial–ethnic group in an approach analogous to the small area mortality models:

$$Y_{j,t,r} \sim Binomial(p_{j,t,r}, \hat{n}_{eff,j,t,r})$$

$$\mathrm{logit}\left( p_{j,t,r} \right)= \beta_{0}+\gamma_{1,j}+\gamma_{2,j,t,r}+\gamma_{3,j,t}+\gamma_{4,r}$$

where $Y_{j,t,r}$ is the estimated count of individuals in county *j*, year *t*, and racial–ethnic group *r* with the modelled outcome, among an effective sample size of $\hat{n}_{eff,j,t,r}$ with proportion $p_{i,t,r}$. Model terms consist of:

- $\beta_{0}$ is a global intercept with a Normal(0, 10) prior;
- $\gamma_{1,j}$ is a random intercept for county with a Besag-York-Mollie-type prior (BYM2) combining a conditional autoregressive distribution for spatial autocorrelation, based on county adjacency, with independent-and-identically-distributed (IID) Gaussian distribution;
- $\gamma_{2,j,t,r}$ is a random intercept for county, racial–ethnic group, and year combinations with a first-order autoregressive (AR1) temporal prior, grouped by county and racial–ethnic group;
- $\gamma_{3,j,t}$ is a random intercept for county and year combinations with an AR1 temporal prior grouped by county;
- and $\gamma_{4,r}$ is a random intercept for racial–ethnic group with an IID Gaussian prior.

Default INLA hyperpriors were used. The BYM2 parameterisation includes a parameter, $\varphi$, which indicates the contribution of the structured spatial effect to the marginal variance;$\mathrm{logit}(\varphi)$had a penalised complexity (PC) prior^14^ corresponding to $\Pr(\varphi< 0.5) = 0.5$, and the log precision of the BYM2 model had a PC prior corresponding to $\Pr(\sigma> 1.0) = 0.1$. The AR1 models had Gamma(shape = 1·0, inverse-scale = $5\cdot{10}^{-5}$) priors on the precision and Normal(mean = 0·0, precision = 0·15) priors on the logit of the 1-year lagged correlation ($\rho$). The race–ethnicity IID term had a Gamma(shape = 1·0, inverse-scale = ${5\cdot10}^{-5}$) prior on the precision. These default priors were used as we considered them suitably vague, in the absence of a priori information with which to establish more informative priors. In INLA, a Gaussian approximation strategy, an empirical Bayes integration strategy, and a step-length for hyperparameter gradient calculations of $1\cdot{10}^{-3}$ were utilised for model fitting. Due to numerical instability in the model for foreign-born proportion, a series of model fits were performed with iteratively decreasing values added to the diagonal of the joint precision matrix (100, 10, and 1, respectively) to derive starting parameter values for the final model run. Mean posterior predictions from the small area covariate models were used as covariate estimates in the small area mortality models.

## 2.4 Small area model specification

Although the focus of this paper is on stomach cancer, almost all causes of death in the GBD cause list were analysed concurrently. This is discussed in Section 2.7 (Calibration). Because of this, the following section includes descriptions of models that are not limited to stomach cancer.

### County and racial–ethnic group model

Separate models were fitted for each cause of death at each level in the GBD hierarchy. The two exceptions to this were Level 1 cause “injuries” and Level 2 cause “maternal and neonatal disorders.” In both cases, instead of modelling these causes directly, the estimates for each cause were constructed by summing the estimates from the separate models for component models. For Level 1 “injuries,” this approach was taken because of the effect of large shocks in the mortality trends due to large-scale disasters, such as Hurricane Katrina (included in Level 2 “unintentional injuries”) and the September 11, 2001 terrorist attacks (included in Level 2 “Self-harm and interpersonal violence”). These shocks—which vary especially drastically by county, age, and racial–ethnic group—were not well modelled using a higher level “injuries” model, and aggregating these child causes proved to be better at fitting these shocks. The estimates for Level 2 “maternal and neonatal disorders” were formed by aggregating the component Level 3 causes “maternal disorders” and “neonatal disorders.” This was done because the age groups for these two component causes are entirely distinct. For most causes, the following model was estimated separately for males and females:

$$D_{j,t,a,r}\sim\mathrm{Poisson}\left( m_{j,t,a,r}\cdot P_{j,t,a,r} \right)$$

$$\log\left( m_{j,t,a,r} \right)=\beta_{0}+\boldsymbol{\beta}_{\boldsymbol{1}}\boldsymbol{\cdot X}_{\boldsymbol{1,j,t}}+\left( \boldsymbol{\beta}_{\boldsymbol{2}}+\boldsymbol{\gamma}_{\boldsymbol{1,r}} \right)\cdot\boldsymbol{X}_{\boldsymbol{2,j,t,r}}+\gamma_{2,j}+\gamma_{3,t,a,r}+\sum_{t^{'}=1}^{k_{t}} \sum_{a^{'}=1}^{k_{a}} \left( \gamma_{4,j,t^{'},a^{'},r}\cdot S_{t^{'}}\left( t \right)\cdot S_{a^{'}}\left( a \right) \right)$$

Priors:

$\boldsymbol{\gamma}_{\boldsymbol{1}} \sim\mathrm{IID}(\boldsymbol{\sigma}_{\boldsymbol{1}})$

$\boldsymbol{\gamma}_{\boldsymbol{2}} \sim\mathrm{LCAR}(\rho_{2},\sigma_{2})$

$$\boldsymbol{\gamma}_{\boldsymbol{3}} \sim LCAR:LCAR:IID(\rho_{3,t},\rho_{3,a},\sigma_{3})$$

$\boldsymbol{\gamma}_{\boldsymbol{4}} \sim LCAR:LCAR:LCAR:IID(\rho_{4,a^{'}}$,$\rho_{4,t^{'}}$,$\rho_{4,j}, \boldsymbol{\sigma}_{\boldsymbol{4}})$

Hyperpriors:

$$\sigma^{-2}\sim PC\left( \sigma_{0}=5, \alpha=0.05 \right)\to\Pr\left( \sigma>5 \right)=0.05$$

$$\mathrm{logit}\left( \rho\right)\sim\mathrm{Normal}\left( 0, 1.5 \right)$$

where

- $j$, $t$, $a$, and $r$ are indices for the county, calendar year (2000–2019, recoded sequentially from 0 to 19), age group (0, 1–4, 5–9, …, 80–84, and 85+ years old [or a subset, for causes that do not affect all age groups], recoded sequentially from 0 to 18), and racial–ethnic group (Latino, Black, White, AIAN, and Asian, recoded in that order from 0 to 4), respectively;
- $D_{j,t,a,r}$ and $P_{j,t,a,r}$ are the observed number of deaths and the population count, respectively, in county $j$, year $t$, age group $a$, and racial–ethnic group $r$;
- $m_{j,t,a,r}$ is the underlying mortality rate in county $j$, year $t$, age group $a$, and racial–ethnic group $r$;
- $\boldsymbol{X}_{\boldsymbol{1,j,t}}$ is a vector of covariates for county $j$ and year $t$, and $\boldsymbol{\beta}_{\boldsymbol{1}}$ is the associated vector of regression coefficients;
- $\boldsymbol{X}_{\boldsymbol{2,j,t,r}}$ is a vector of covariates for county $j$, year $t$, and racial–ethnic group $r$, and $\boldsymbol{\beta}_{\boldsymbol{2}}+\boldsymbol{\gamma}_{\boldsymbol{1,r}}$ is the associated vector of regression coefficients, made up of a fixed component ($\boldsymbol{\beta}_{\boldsymbol{2}}$) shared by all racial–ethnic groups, and a random slope component ($\boldsymbol{\gamma}_{\boldsymbol{1,r}}$) that varies by racial–ethnic group;
- $\gamma_{2,j}$ is a random intercept for county;
- $\gamma_{3,a,t,r}$ is a random intercept for age group, year, and racial–ethnic group;
- $\gamma_{4,j,t^{'},a^{'},r}$ is a random intercept for county, racial–ethnic group, year spline basis, and age spline basis combinations;
- $k_{t}$ is the number of time knots (four knots, evenly spaced from 2000–2019) and $t'$ is the corresponding index;
- $k_{a}$ is the number of age knots, which varies by cause due to age restrictions (for causes with all age groups included, there are five knots at age groups 0, 25–29, 45–49, 65–69, and 85+ years old, but if the minimum age is less than 25 or maximum age is less than 85+, the initial and final knots are adjusted to start and end at these values, although adjacent knots such as 40–44 and 45–49 are avoided by choosing a different second knot, for example 55–59; an exception is given for neonatal disorders, which has knots at ages 0 and 1; maternal disorders has only two age knots at 10 and 50 due to challenges modelling small numbers) and $a'$ is the corresponding index;
- $S_{t^{'}}\left( t \right)$ is the value of spline basis $t^{'}$ for a linear spline on year, evaluated at year $t$;
- and $S_{a^{'}}\left( a \right)$ is the value of spline basis $a'$ for a linear spline on age, evaluated at age group $a$

Prior distributions were assigned for each random component:

- Each element of $\boldsymbol{\gamma}_{\boldsymbol{1}}$—corresponding to each covariate in $\boldsymbol{X}_{\boldsymbol{1}}$—was assumed to follow an independent and identically distributed (IID) mean-0 Normal distribution. $\boldsymbol{\gamma}_{\boldsymbol{1}}$ is associated with three hyperparameters ($\boldsymbol{\sigma}_{\boldsymbol{1}}^{\boldsymbol{2}}$), corresponding to the variance of this random intercept for each covariate.
- $\boldsymbol{\gamma}_{\boldsymbol{2}}$ was assumed to follow a conditional autoregressive distribution of the form described by Leroux, Lei, and Breslow,^15^ which corresponds to the following full conditional distribution for each individual element of $\boldsymbol{\gamma}_{\boldsymbol{2}}$:

$$\gamma_{j}|\gamma_{k\sim j},\sigma^{2},\rho\sim\mathrm{Normal}\left( \frac{\rho\cdot\sum_{k\sim j} \gamma_{k}}{n_{j}\cdot\rho+1-\rho},\frac{\sigma^{2}}{n_{j}\cdot\rho+1-\rho} \right)$$

where $k \sim j$ indicates the set of counties that are adjacent to county $j$ and $n_{j}$ is the number of counties in $k \sim j$. In this distribution, the $\sigma^{2}$ parameter controls the degree of spatial variation and the $\rho$ parameter, which varies between 0 and 1, determines the degree of spatial smoothness.

- $\boldsymbol{\gamma}_{\boldsymbol{3}}$ was assumed to follow a mean-0, multivariate Normal distribution with a separable covariance structure defined via the Kronecker product of the covariance matrix of three distributions:^16,17^ a conditional autoregressive distribution as in $\gamma_{2}$ but defined for age groups rather than counties; a second conditional autoregressive distribution as in $\gamma_{2}$ but defined for years rather than counties; and a multivariate Normal distribution with a diagonal covariance matrix. This random intercept is associated with three hyperparameters: $\rho_{3,a}$ and $\rho_{3,t}$, which control the correlation across age groups and time, respectively; and $\sigma_{3}^{2}$ which controls the degree of variation.
- $\boldsymbol{\gamma}_{\boldsymbol{4}}$ was also assumed to follow a mean-0, multivariate Normal distribution, in this case with a separable covariance structure defined via the Kronecker product of the covariance matrix of four distributions: three conditional autoregressive distributions as described above, for county, age spline basis, and year spline basis; and an IID Normal distribution for racial–ethnic group. For this random intercept, there are two sets of hyperparameters: $\rho_{4,a^{'}}$,$\rho_{4,t^{'}}$, and $\rho_{4,j}$, which control the smoothness over the age spline, year spline, and county, respectively; and $\sigma_{4}^{2}$ which controls the degree of variation.

Finally, hyperpriors were defined for the standard deviation ($\sigma$) and, where applicable, autocorrelation ($\rho$) hyperparameters:

- Penalised complexity (PC) priors were specified for the inverse variance ($1/{\sigma^{2}}$) of each random effect.^14^ PC priors shrink toward a base model, which in this case is where the marginal variance is 0. They are specified by setting the tail probability on each hyperparameter. We followed the recommendation by Fuglstad et al.,^14^ selecting priors that satisfy$Pr(\sigma>\sigma_{0}) = 0.05$ where $\sigma_{0}$ is between 2.5 and 40 times the expected true marginal standard deviation. Specifically, we set $\sigma_{0}=5; Pr(\sigma>\sigma_{0}) = 0.05$.
- Normal(0, 1.5) priors were specified for the logit-transform of the correlation parameters ($\rho$).

Posterior means and standard errors for these hyperparameters are provided in section 3.6.

Descriptively, this model specifies the log of the underlying mortality rate ($m_{j,t,a,r}$) as a function of covariates and additional variation by county, year, age, and racial–ethnic group. The covariates we included in this model—educational attainment, poverty rate, proportion foreign-born, median household income, and population density—were selected based on data availability and previously observed relationships with all-cause mortality. The fixed effects ($\boldsymbol{\beta}_{\boldsymbol{1}}$**,** $\boldsymbol{\beta}_{\boldsymbol{2}}$) on covariates at the county level and covariates specific to county and racial–ethnic group combinations($\boldsymbol{X}_{\boldsymbol{1,j,t}}\boldsymbol{,}\boldsymbol{X}_{\boldsymbol{2,j,t,r}}$) capture the relationships between each covariate and mortality. For the covariates by racial–ethnic group, we additionally include random slopes, $\boldsymbol{\gamma}_{\boldsymbol{1}}$, to allow for the relationship between these variables and mortality to vary by racial–ethnic group. The covariates do not explain all variation in mortality across time, age, geography, and racial–ethnic group, so further random intercepts are included in the model to capture additional variation. The random intercept $\boldsymbol{\gamma}_{\boldsymbol{2}}$ allows for spatial (ie, between-county) variation in the level of mortality, shared across age, year, and racial–ethnic group. The random intercept $\boldsymbol{\gamma}_{\boldsymbol{3}}$ allows for variation in mortality by age, time, and racial–ethnic group, shared across all counties. Finally, the random intercept $\boldsymbol{\gamma}_{\boldsymbol{4}}$ was included to allow for county-specific deviations in the mortality patterns by age, time, and racial–ethnic group, compared with the general pattern captured by $\boldsymbol{\gamma}_{\boldsymbol{3}}$. This random intercept incorporates a linear spline in the age and time dimensions to reduce computational complexity: the equivalent model for all age groups and years was found to be computationally infeasible. Although the splines in this random intercept are linear, we are not assuming that the time or age trends for $\log\left( m_{j,t,a,r} \right)$ are linear, as both the contributions from the covariates as well as $\boldsymbol{\gamma}_{\boldsymbol{3}}$ allow for non-linear variation. The number of age and year knots were chosen to maximise flexibility while maintaining a reasonable runtime.

We used the Template Model Builder (TMB) package^18^ in R version 3.6.1^13^ to fit these models using an empirical Bayes approach. TMB calculates analytic approximations to the posterior distribution based on Laplace approximations. We use TMB for fitting these models rather than INLA—another common alternative to classic Markov chain Monte Carlo (MCMC) methods and the tool that we used for the covariate imputation and smoothing models (section 2.3)—as TMB is substantially more flexible with respect to the model specification.^19^ Of particular importance for this analysis: random effects in INLA are restricted to two-way interactions, whereas TMB allows us to incorporate higher-order interactions ($\boldsymbol{\gamma}_{\boldsymbol{3}}$ and $\boldsymbol{\gamma}_{\boldsymbol{4}}$) in our modelling approach.

In addition to the more formal model validation we describe below, we assessed model fit by inspecting plots comparing the estimated time and age trends in mortality to the observed data at the national, state, and (in selected counties) county level. Additionally, for the all-cause mortality model we used graphical posterior predictive checks^20^ to assess if the observed data are over-dispersed or zero-inflated relative to our model; we found no evidence that this is the case. Finally, we examined plots of the binned residuals compared with each covariate to assess the assumption that the relationship between log mortality and each covariate is linear; we found no evidence of bias in our estimates as a result of non-linearity in these relationships.

#### Cause-specific model exceptions

The cause-specific models described above tend to smooth mortality over time and thus can perform poorly in cases where there are sudden substantial changes in mortality, particularly when those changes are short-term. In particular, we find that this model performed poorly when modelling intentional injuries, due to the pronounced increase in observed deaths as a consequence of the September 11, 2001 terrorists attacks, and when modelling unintentional injuries, due to the large increase in observed deaths as consequence of Hurricane Katrina in 2005.

In order to better reflect the impact of these events in our mortality estimates for intentional injuries and unintentional injuries, we added two additional random slopes (one by county, and one by age) to the models for these two causes, both of which are multiplied by an indicator variable $I$ that is 1 for counties where there was at least one observed death due to the event in question, and 0 otherwise. For self-harm and interpersonal violence, ICD code U01.1 was used to identify deaths in 2001 that were due to 9/11. For unintentional injuries, ICD code X37.0 is used to identify deaths in 2005 that were likely due to Hurricane Katrina. With these additional random slopes, the model is specified as follows:

$$D_{j,t,a,r}\sim\mathrm{Poisson}\left( m_{j,t,a,r}\cdot P_{j,t,a,r} \right)$$

$$\log\left( m_{j,t,a,r} \right)=\beta_{0}+\boldsymbol{\beta}_{\boldsymbol{1}}\boldsymbol{\cdot X}_{\boldsymbol{1,j,t}}+\left( \boldsymbol{\beta}_{\boldsymbol{2}}+\boldsymbol{\gamma}_{\boldsymbol{1,r}} \right)\cdot\boldsymbol{X}_{\boldsymbol{2,j,t,r}}+\gamma_{2,j}+\gamma_{3,t,a,r}+\sum_{t^{'}=1}^{k_{t}} \sum_{a^{'}=1}^{k_{a}} \left( \gamma_{4,j,t^{'},a^{'},r}\cdot S_{t^{'}}\left( t \right)\cdot S_{a^{'}}\left( a \right) \right)+\gamma_{5,j}\cdot I_{j,t}+\gamma_{6,a}\cdot I_{j,t}$$

where:

$$\boldsymbol{\gamma}_{\boldsymbol{5}} \sim\mathrm{IID}(\sigma_{5})$$

$$\sigma_{5}^{-2}\sim\mathrm{PC}\left( \sigma_{0}=5, \alpha=0.05 \right)\to\Pr\left( \sigma>5 \right)=0.05$$

$$\boldsymbol{\gamma}_{\boldsymbol{6}} \sim\mathrm{IID}(\sigma_{6})$$

$$\sigma_{6}^{-2}\sim\mathrm{PC}\left( \sigma_{0}=5, \alpha=0.05 \right)\to\Pr\left( \sigma>5 \right)=0.05$$

Additionally, in order to better reflect these sharp increases in mortality throughout the cause hierarchy, we added random slopes for both of these events to the all-cause model, which was thus specified as follows:

$$D_{j,t,a,r}\sim\mathrm{Poisson}\left( m_{j,t,a,r}\cdot P_{j,t,a,r} \right)$$

$$\log\left( m_{j,t,a,r} \right)=\beta_{0}+\boldsymbol{\beta}_{\boldsymbol{1}}\boldsymbol{\cdot X}_{\boldsymbol{1,j,t}}+\left( \boldsymbol{\beta}_{\boldsymbol{2}}+\boldsymbol{\gamma}_{\boldsymbol{1,r}} \right)\cdot\boldsymbol{X}_{\boldsymbol{2,j,t,r}}+\gamma_{2,j}+\gamma_{3,t,a,r}+\sum_{t^{'}=1}^{k_{t}} \sum_{a^{'}=1}^{k_{a}} \left( \gamma_{4,j,t^{'},a^{'},r}\cdot S_{t^{'}}\left( t \right)\cdot S_{a^{'}}\left( a \right) \right)+\gamma_{5,j}\cdot I_{j,t(2001)} +\gamma_{6,j}\cdot I_{j,t(2005)}+ \gamma_{7,a}\cdot I_{j,t(2001)}+ \gamma_{8,a}\cdot I_{j,t(2005)}$$

where:

$$\boldsymbol{\gamma}_{\boldsymbol{5}} \sim\mathrm{IID}(\sigma_{5})$$

$$\sigma_{5}^{-2}\sim\mathrm{PC}\left( \sigma_{0}=5, \alpha=0.05 \right)\to\Pr\left( \sigma>5 \right)=0.05$$

$$\boldsymbol{\gamma}_{\boldsymbol{6}} \sim\mathrm{IID}(\sigma_{6})$$

$$\sigma_{6}^{-2}\sim\mathrm{PC}\left( \sigma_{0}=5, \alpha=0.05 \right)\to\Pr\left( \sigma>5 \right)=0.05$$

$$\boldsymbol{\gamma}_{\boldsymbol{7}} \sim\mathrm{IID}(\sigma_{7})$$

$$\sigma_{7}^{-2}\sim\mathrm{PC}\left( \sigma_{0}=5, \alpha=0.05 \right)\to\Pr\left( \sigma>5 \right)=0.05$$

$$\boldsymbol{\gamma}_{\boldsymbol{8}} \sim\mathrm{IID}(\sigma_{8})$$

$$\sigma_{8}^{-2}\sim\mathrm{PC}\left( \sigma_{0}=5, \alpha=0.05 \right)\to\Pr\left( \sigma>5 \right)=0.05$$

### County-level model

Similar models were estimated for each cause for all racial–ethnic groups combined. These models were specified as follows, with all terms defined as described above:

$$D_{j,t,a}\sim\mathrm{Poisson}\left( m_{j,t,a}\cdot P_{j,t,a} \right)$$

$$\log\left( m_{j,t,a} \right)=\beta_{0}+\boldsymbol{\beta}_{\boldsymbol{1}}\boldsymbol{\cdot X}_{\boldsymbol{1,j,t}}+\gamma_{2,j}+\gamma_{3,t,a}+\sum_{t^{'}=1}^{k_{t}} \sum_{a^{'}=1}^{k_{a}} \left( \gamma_{4,j,t^{'},a^{'}}\cdot S_{t^{'}}\left( t \right)\cdot S_{a^{'}}\left( a \right) \right)$$

Priors:

$\boldsymbol{\gamma}_{\boldsymbol{2}} \sim\mathrm{LCAR}(\rho_{2},\sigma_{2})$

$$\boldsymbol{\gamma}_{\boldsymbol{3}} \sim LCAR:LCAR(\rho_{3,t},\rho_{3,a},\sigma_{3})$$

$\boldsymbol{\gamma}_{\boldsymbol{4}} \sim LCAR:LCAR:LCAR(\rho_{4,a^{'}}$,$\rho_{4,t^{'}}$,$\rho_{4,j}, \sigma_{4})$

Hyperpriors:

$$\sigma^{-2}\sim\mathrm{PC}\left( \sigma_{0}=5, \alpha=0.05 \right)\to\Pr\left( \sigma>5 \right)=0.05$$

$$\mathrm{logit}\left( \rho\right)\sim\mathrm{Normal}\left( 0, 1.5 \right)$$

#### Cause-specific model exceptions

Similar to the model specific to county and racial–ethnic group, an additional random slope was included for the models of unintentional injuries and self-harm and interpersonal violence. The indicators were created in the same way. Thus, the model is as follows:

$$D_{j,t,a,r}\sim\mathrm{Poisson}\left( m_{j,t,a,r}\cdot P_{j,t,a,r} \right)$$

$$\log\left( m_{j,t,a,r} \right)=\beta_{0}+\boldsymbol{\beta}_{\boldsymbol{1}}\boldsymbol{\cdot X}_{\boldsymbol{1,j,t}}+\left( \boldsymbol{\beta}_{\boldsymbol{2}}+\boldsymbol{\gamma}_{\boldsymbol{1,r}} \right)\cdot\boldsymbol{X}_{\boldsymbol{2,j,t,r}}+\gamma_{2,j}+\gamma_{3,t,a,r}+\sum_{t^{'}=1}^{k_{t}} \sum_{a^{'}=1}^{k_{a}} \left( \gamma_{4,j,t^{'},a^{'},r}\cdot S_{t^{'}}\left( t \right)\cdot S_{a^{'}}\left( a \right) \right)+\gamma_{5,j}\cdot I_{j,t}+\gamma_{6,a}\cdot I_{j,t}$$

where:

$$\boldsymbol{\gamma}_{\boldsymbol{5}} \sim\mathrm{IID}(\sigma_{5})$$

$$\sigma_{5}^{-2}\sim PC\left( \sigma_{0}=5, \alpha=0.05 \right)\to\Pr\left( \sigma>5 \right)=0.05$$

$$\boldsymbol{\gamma}_{\boldsymbol{6}} \sim\mathrm{IID}(\sigma_{6})$$

$$\sigma_{6}^{-2}\sim\mathrm{PC}\left( \sigma_{0}=5, \alpha=0.05 \right)\to\Pr\left( \sigma>5 \right)=0.05$$

Additionally, the all-cause model was constructed similarly:

$$D_{j,t,a,r}\sim\mathrm{Poisson}\left( m_{j,t,a,r}\cdot P_{j,t,a,r} \right)$$

$$\log\left( m_{j,t,a,r} \right)=\beta_{0}+\boldsymbol{\beta}_{\boldsymbol{1}}\boldsymbol{\cdot X}_{\boldsymbol{1,j,t}}+\left( \boldsymbol{\beta}_{\boldsymbol{2}}+\boldsymbol{\gamma}_{\boldsymbol{1,r}} \right)\cdot\boldsymbol{X}_{\boldsymbol{2,j,t,r}}+\gamma_{2,j}+\gamma_{3,t,a,r}+\sum_{t^{'}=1}^{k_{t}} \sum_{a^{'}=1}^{k_{a}} \left( \gamma_{4,j,t^{'},a^{'},r}\cdot S_{t^{'}}\left( t \right)\cdot S_{a^{'}}\left( a \right) \right)+\gamma_{5,j}\cdot I_{j,t(2001)} +\gamma_{6,j}\cdot I_{j,t(2005)}+ \gamma_{7,a}\cdot I_{j,t(2001)}+ \gamma_{8,a}\cdot I_{j,t(2005)}$$

where:

$$\boldsymbol{\gamma}_{\boldsymbol{5}} \sim\mathrm{IID}(\sigma_{5})$$

$$\sigma_{5}^{-2}\sim\mathrm{PC}\left( \sigma_{0}=5, \alpha=0.05 \right)\to\Pr\left( \sigma>5 \right)=0.05$$

$$\boldsymbol{\gamma}_{\boldsymbol{6}} \sim\mathrm{IID}(\sigma_{6})$$

$$\sigma_{6}^{-2}\sim\mathrm{PC}\left( \sigma_{0}=5, \alpha=0.05 \right)\to\Pr\left( \sigma>5 \right)=0.05$$

$$\boldsymbol{\gamma}_{\boldsymbol{7}} \sim\mathrm{IID}(\sigma_{7})$$

$$\sigma_{7}^{-2}\sim\mathrm{PC}\left( \sigma_{0}=5, \alpha=0.05 \right)\to\Pr\left( \sigma>5 \right)=0.05$$

$$\boldsymbol{\gamma}_{\boldsymbol{8}} \sim\mathrm{IID}(\sigma_{8})$$

$$\sigma_{8}^{-2}\sim\mathrm{PC}\left( \sigma_{0}=5, \alpha=0.05 \right)\to\Pr\left( \sigma>5 \right)=0.05$$

### Hyperprior sensitivity analysis

In previous work on all-cause mortality and life expectancy,^1^ we tested a wide range of alternative hyperparameter specifications. Because testing all of these combinations across all causes would be computationally intractable, we chose a subset of both hyperparameter specifications and causes for the purposes of testing the effect of these by cause. Thus, a variety of penalised complexity (PC) specifications were chosen:

- $\sigma_{0} = 1; \Pr\left( \sigma>\sigma_{0} \right)= 0.02,0.05, 0.1$
- $\sigma_{0} = 5; \Pr\left( \sigma>\sigma_{0} \right)=0.1$

To test a range of models with varying burden, age restrictions, sex restrictions, and mortality patterns, the following Level 2 causes were modelled by use of each of the above hyperparameter combinations:

- Enteric infections
- HIV/AIDS & STIs
- Maternal disorders
- Neoplasms
- Substance use

The model results were very similar across parameterisations. The figure in section 4.2 shows the difference in the estimates derived from models using these alternate prior specifications compared with our main model. These differences are generally very small across all parameterisations and prior specifications.

## 2.5 Small area model validation

### Approach

The framework used to evaluate the performance of the models in this analysis is an extension of a previously proposed^21^ and extensively used framework designed specifically for county-level models in the USA. Previously,^1^ this was modified to allow for evaluation of performance with respect to generating estimates of life expectancy by county and racial–ethnic group. Here, we use this framework to evaluate performance of the same model with respect to producing estimates of age-standardised mortality and to compare performance of two simplified versions of this model.

First, a “validation set” of county and racial–ethnic group pairs was identified. The validation set is a collection of county and racial–ethnic group pairs for which directly calculated mortality rates are a good representation of the underlying mortality rate—ie, where the population and corresponding number of deaths is sufficiently large to generate stable direct estimates. To increase the number of county and racial–ethnic group combinations that were retained in the validation set, deaths and population data were pooled across time by use of a moving window of three years for the purposes of defining the validation set. A series of criteria were used to remove county and racial–ethnic group combinations from the set of all county and racial–ethnic group combinations, with the remainder forming the validation set. First, any county and racial–ethnic group combination with zero pooled deaths in any age, sex, and year (window) combination were removed. Second, among the remaining county and racial–ethnic group combinations, those where the median (across all years, sexes, and age groups) coefficient of variation was greater than 20% for the age-specific mortality rates or greater than 5% for the age-standardised and crude mortality rates were removed. In order to calculate the coefficient of variation, 1000 draws of death counts were simulated for each age group, sex, and year, assuming a Poisson distribution with rate and size equal to the observed mortality rate and population size, respectively; age-specific, crude (all-ages), and age-standardised mortality rates were then calculated for each draw; and finally the coefficient of variation was calculated as the standard deviation of the draws divided by their mean. The validation set has 138 county and racial–ethnic group pairs, composed of 96 counties and four racial–ethnic groups (section 3.7). The AIAN group was not represented in the validation set. These pooled mortality rates were used as a “gold standard” against which to compare model predictions.

Next, “validation datasets” were constructed. A total of 40 validation sets were used: 5 iterations each of simulated datasets with reference population sizes of 10, 100, 1000, 3000, 5000, 10 000, 25 000, and 100 000. Additionally, a sixth iteration was prepared for each of these population sizes because, rarely, models for some iterations failed to converge; this sixth iteration was only used if a model failed to converge. The sizes 1000–100 000 were chosen because they correspond roughly to the 1st, 5th, 10th, 25th, 50th, and 80th percentiles for the total population (all racial–ethnic groups combined) across all counties and years. Sizes 10 and 100 were added because similar population sizes are common for specific racial–ethnic groups at the county level. To make these validation data sets, a population of the specified size was sampled for each county and racial–ethnic group in the validation set, with the probability of sampling an individual of a given age and sex proportional to the observed population structure for that county and racial–ethnic group. Then, for each age and sex within each county and racial–ethnic group, deaths were simulated from a Poisson distribution with mean equal to the observed mortality rate times the sampled population for that age group and sex. The data for all county and racial–ethnic group pairs that were not in the validation set were included without modification.

Finally, small area models were fit and predictions generated as described above to each of the 40 validation datasets. To evaluate the performance, the predictions based on each validation data set were compiled and compared against the gold standard mortality rates. First, errors were calculated as $m_{j,t,r}^{gs}-m_{j,t,a,r}^{pred}$ where $m_{j,t,r}^{gs}$ is the gold standard age-standardised mortality rate per 100 000, and $m_{j,t,r}^{pred}$ is the predicted age-standardised mortality rate per 100 000. The mean error and the mean absolute error—measures of bias and precision, respectively—were calculated across all county and racial–ethnic group combinations in the validation set and all iterations at each population level. Coverage (ie, the percentage of county, racial–ethnic group, and year combinations where the gold standard estimate was between the lower and upper uncertainty intervals for the modelled estimate) was also recorded.

This validation procedure was used to assess and compare model performance of three models:

1. The model for all-cause mortality used previously to estimate life expectancy:

$$D_{j,t,a,r}\sim\mathrm{Poisson}\left( m_{j,t,a,r}\cdot P_{j,t,a,r} \right)$$

$$\log\left( m_{j,t,a,r} \right)=\beta_{0}+\boldsymbol{\beta}_{\boldsymbol{1}}\boldsymbol{\cdot X}_{\boldsymbol{1,j,t}}+\left( \boldsymbol{\beta}_{\boldsymbol{2}}+\boldsymbol{\gamma}_{\boldsymbol{1,r}} \right)\cdot\boldsymbol{X}_{\boldsymbol{2,j,t,r}}+\gamma_{2,j}+\gamma_{3,t,a,r}+\sum_{t^{'}=1}^{k_{t}} \sum_{a^{'}=1}^{k_{a}} \left( \gamma_{4,j,t^{'},a^{'},r}\cdot S_{t^{'}}\left( t \right)\cdot S_{a^{'}}\left( a \right) \right)$$

where:

$\boldsymbol{\gamma}_{\boldsymbol{4}} \sim LCAR:LCAR:LCAR:MVN(\rho_{4,a^{'}}$,$\rho_{4,t^{'}}$,$\rho_{4,j}, \boldsymbol{\sigma}_{\boldsymbol{4}},\boldsymbol{\theta})$

1. The model described above for most causes (except all-cause mortality, unintentional injuries, and self-harm and interpersonal violence, which have additional components to specifically model increases in mortality due to the 9/11 terrorist attacks and Hurricane Katrina):

$$D_{j,t,a,r}\sim\mathrm{Poisson}\left( m_{j,t,a,r}\cdot P_{j,t,a,r} \right)$$

$$\log\left( m_{j,t,a,r} \right)=\beta_{0}+\boldsymbol{\beta}_{\boldsymbol{1}}\boldsymbol{\cdot X}_{\boldsymbol{1,j,t}}+\left( \boldsymbol{\beta}_{\boldsymbol{2}}+\boldsymbol{\gamma}_{\boldsymbol{1,r}} \right)\cdot\boldsymbol{X}_{\boldsymbol{2,j,t,r}}+\gamma_{2,j}+\gamma_{3,t,a,r}+\sum_{t^{'}=1}^{k_{t}} \sum_{a^{'}=1}^{k_{a}} \left( \gamma_{4,j,t^{'},a^{'},r}\cdot S_{t^{'}}\left( t \right)\cdot S_{a^{'}}\left( a \right) \right)$$

Where:

$\boldsymbol{\gamma}_{\boldsymbol{4}} \sim LCAR:LCAR:LCAR:IID(\rho_{4,a^{'}}$,$\rho_{4,t^{'}}$,$\rho_{4,j}, \sigma_{4})$

1. The same model as (2), but with a $\boldsymbol{\sigma}_{\boldsymbol{4}}^{2}$ (length = 5) term that controls the variation for each racial–ethnic group separately, thus allowing further variation across racial–ethnic groups.

$$D_{j,t,a,r}\sim\mathrm{Poisson}\left( m_{j,t,a,r}\cdot P_{j,t,a,r} \right)$$

$$\log\left( m_{j,t,a,r} \right)=\beta_{0}+\boldsymbol{\beta}_{\boldsymbol{1}}\boldsymbol{\cdot X}_{\boldsymbol{1,j,t}}+\left( \boldsymbol{\beta}_{\boldsymbol{2}}+\boldsymbol{\gamma}_{\boldsymbol{1,r}} \right)\cdot\boldsymbol{X}_{\boldsymbol{2,j,t,r}}+\gamma_{2,j}+\gamma_{3,t,a,r}+\sum_{t^{'}=1}^{k_{t}} \sum_{a^{'}=1}^{k_{a}} \left( \gamma_{4,j,t^{'},a^{'},r}\cdot S_{t^{'}}\left( t \right)\cdot S_{a^{'}}\left( a \right) \right)$$

Where:

$\boldsymbol{\gamma}_{\boldsymbol{4}} \sim LCAR:LCAR:LCAR:IID(\rho_{4,a^{'}}$,$\rho_{4,t^{'}}$,$\rho_{4,j}, \boldsymbol{\sigma}_{\boldsymbol{4}})$

$$\boldsymbol{\sigma}_{\boldsymbol{4}\boldsymbol{i}}^{\boldsymbol{-2}}\boldsymbol{\sim}\mathrm{PC}\left( \sigma_{0}=5, \alpha=0.05 \right)\to\Pr\left( \sigma>5 \right)=0.05$$

The variant of the model that reduces the racial–ethnic group component of $\boldsymbol{\gamma}_{\boldsymbol{4}}$ to IID (model 2) was included to assess the performance of the model that was most computational feasible to run for all causes. The variant of the model with an identically (but not independent) distributed $\boldsymbol{\gamma}_{\boldsymbol{4}}$ racial–ethnic group intercept (model 3) was included to assess the importance of separate variance terms by racial–ethnic group.

### Results

The mean relative error, mean absolute relative error, and coverage for each model are provided in sections 3.8 (table) and 4.2 (figure). The mean relative error is a measure of relative bias—ie, it indicates if the estimates from the models were systematically higher or lower than the gold standard values and is measured as a percent difference compared with the gold standard. As expected, mean relative error tended to improve (was closer to 0%) as the simulated population size increased for all models and all racial–ethnic groups. In model 1, there was evidence of bias for most racial–ethnic groups in populations < 10 000, although the mean relative errors were minimal at the two larger population sizes. At size 10, the model overestimated the age-standardised mortality rate for the Asian, Black, and Latino groups by 8.67%, 2.9%, and 2.57%, respectively. However, it underestimated for the White group by 1.07%. Both model 2 and 3 performed better than model 1 in terms of mean relative error at all population sizes except 100 000. However, the mean relative error was still quite large for the Asian group; at size 10, model 2 and model 3 overestimated by 7.77% and 6.98%, respectively. However, the Asian group only had four counties in the validation set, so it is difficult to draw general conclusions about bias for this racial–ethnic group. Across all racial–ethnic groups, the mean relative errors for model 2 were smaller in magnitude compared with the other models for about 70% of racial–ethnic groups and population sizes, but they were similar to those from model 3.

The mean absolute relative error is a measure of overall error—ie, how much the model estimates differ from true estimates irrespective of direction. It is also measured as a percent difference compared with the gold standard. As expected, mean absolute relative errors tended to improve as size increased for all models and all racial–ethnic groups. All three models performed similarly across population sizes, although model 1 performed slightly better for about 65% of the population sizes and racial–ethnic groups. Except for the estimates for the White population, model 1 did not perform as well at population sizes 10 and 100.

Coverage is a measure of the calibration of the uncertainty intervals. Ideally, coverage should be close to 95% because that means that the 95% uncertainty intervals were an appropriate reflection of an estimate’s uncertainty. Coverage was similarly close to 95% in all models: across all racial–ethnic groups, model 1 had three coverage values within +/- 1 percentage point of 95, whereas models 2 and 3 had two values within this range. Across models, coverage varied similarly across racial–ethnic groups, resulting in a similar range of values. Coverage tended to be highest at the smallest population sizes, and then decreased as population size increased for most models and racial–ethnic groups. This is likely due to the wider uncertainty intervals at smaller population sizes, making it more likely for the gold standard to be included within the uncertainty interval.

## 2.6 Derivation of misclassification ratios by county, age, sex, and racial–ethnic group

### Extraction

Overall misclassification ratios, as well as misclassification ratios by age and sex, census region, and co-ethnic density were extracted from Arias et al.^5^ for five racial–ethnic groups: AIAN, Asian, Black, Latino, and White. Specifically, data were extracted from the following numbered tables in Arias et al.:

- Overall misclassification ratios: Table 2, “Total” column for both sexes.
- Age-sex misclassification ratios: Table 2, age-specific columns for each sex separately.
- Census region misclassification ratios: Table 3, results by “Region”
- Co-ethnic density misclassification ratios: Table 3, results by “Coethnic concentration”; AIAN ratios extracted from the results shown for all AIAN because these were not available specifically for non-Latino AIAN.

The co-ethnic density misclassification ratios were assigned to a county by the same method outlined in Arias et al.^5^ for the AIAN population, counties in Contract Health Service Delivery Areas (CHSDAs) were considered to have high co-ethnic density; for the Latino population, counties with high co-ethnic density were those within the first 50^th^ percentile of ranked deaths by county between 1999 and 2011. This method assumes that the relative increase or decrease in misclassification for counties with low or high co-ethnic density compared with the total misclassification ratio for AIAN (including both Latino and non-Latino AIAN) is representative of that for non-Latino AIAN. We are not aware of any studies on this topic, but we make this assumption to include adjustment by co-ethnic density given the evidence that this form of misclassification is especially important with respect to the AIAN population.^22–24^

### Combination

1000 draws of each misclassification ratio were generated assuming these ratios are log-Normally distributed. We selected a log-Normal distribution as it is restricted to positive numbers, which is appropriate for this ratio; however, we acknowledge that this selection is somewhat arbitrary and is at best an approximation of the true ratio distribution. The draws of misclassification were then combined (without ordering) by the following approach:

$${ratio}_{final}={ratio}_{overall}\cdot\frac{{ratio}_{age,sex}}{{ratio}_{overall}}\cdot\frac{{ratio}_{region}}{{ratio}_{overall}}\cdot\frac{{ratio}_{co-ethnic density}}{{ratio}_{overall}}$$

For the Asian, Black and White populations, ${ratio}_{co-ethnic density}$ is equal to ${ratio}_{overall}$, so the last term is equal to 1 (ie, no adjustment by co-ethnic density). For the AIAN population, the ${ratio}_{overall}$ in the denominator of the final term was the overall misclassification ratio for both Latino and non-Latino AIAN populations combined, to match the numerator, which was available only for Latino and non-Latino AIAN populations combined; in all other terms, the values for the non-Latino AIAN population alone were used.

This approach assumes that the degree of misclassification is independent across each dimension. Thus, the degree of misclassification reported for a particular facet (eg, by age or sex) is the same across all other facets (eg, region and co-ethnic density). For example, the age or sex pattern of misclassification for a given racial–ethnic group will be the same regardless of region.

### Mortality rate adjustment

The 1000 draws of misclassification ratios were merged onto the 1000 posterior draws of the mortality rate by age, sex, county (which reflected both region and co-ethnic density), and racial–ethnic group. The merge by age was conducted by aligning the age groups in the model (0, 1–4, 5–9, …, 80–84, and 85+ years old) with the age bin to which each of the modelled ages belongs (ie, age groups 0, 1–4, 5–9, 10–14, 15–19, and 20–24 were merged onto the age bin 0–24). These draws were not ordered, thus assuming independence between the mortality rate and the misclassification ratio. Mortality rate draws were adjusted by multiplying by the corresponding misclassification ratio draw.

## 2.7 Calibration

After the modelling steps, results were calibrated to ensure consistency across three dimensions: cause hierarchy, racial–ethnic group, and geography. Adjustments by cause were needed for two reasons. First, SAE models were run independently for all-cause mortality and for stomach cancer. Thus, the cause-specific model outputs do not necessarily nest as they should; that is, if all other Level 3 causes of death in the GBD hierarchy were modelled, then adding mortality across all Level 3 causes may not add up to all-cause mortality, although generally it would be close.

Second, adjustments were needed to ensure that the county and racial–ethnic group combination results were consistent with the directly modelled county-level estimates. This was important because the joint effect of modelling by racial–ethnic group and applying the misclassification ratios can lead to results that do not align precisely with the county-level model. Without this calibration step, the misclassification adjustment in particular could lead to inaccurate results given the assumption of independence between the various misclassification ratios (section 2.6). This is in addition to any differences that county-level estimates will have with county and racial–ethnic group estimates that have been population weighted to the county level.

To accomplish these goals, an algorithm known as raking (also called iterative proportional fitting) was utilised. This process has been described in detail previously.^25^ Given an N-dimensional table whose internal entries denote uncertain values and whose marginal entries denote aggregated, more certain values, raking provides a means of fitting the internal entries to the margins while preserving the internal relationships of the table. This is accomplished simply by proportionately scaling the rows to add up to their marginal totals, then scaling the columns the same way, and repeating until the entries in the table stabilise (ie, converge). As such, it is necessary to concurrently model the other causes of death, not just stomach cancer. Due to model instability in very small causes of death, we model causes with at least 10 000 deaths in total over the study period and at least 1000 deaths each among males and females separately.

Raking has been shown to converge if the sum of the margins are equal and there are no zeros or negative numbers in the margins.^26^ Raking works on tables of arbitrary dimensionality and requires only a single iteration for a one-dimensional table. It should be noted that this algorithm was applied to mortality rates rather than death counts, and as such, an extra population-weighting step was required when raking to state estimates.

To rake county-level estimates across causes, one-dimensional raking was applied to fit county-level estimates of lower-level causes to higher-level cause estimates at each level of the cause hierarchy. Because we only model causes with more than 10 000 deaths in total and 1000 deaths among males and females over 2000–2019, we must account for the non-modelled causes before raking. Thus, we assume that any difference between mortality aggregated across child causes compared to the parent cause should be attributed to unmodelled causes in that branch of the cause hierarchy. Specifically, to make this adjustment by county, year, age, sex, and parent cause, we calculated the mean (ie, across draws) mortality rate aggregated across child causes and compared it to the mean mortality rate of the raked parent cause. If this aggregated value was larger than the parent cause value, we assumed that mortality attributed to the unmodelled causes was zero. Otherwise, we divided the aggregated mortality rate by the parent cause mortality rate to get the cause fraction of modelled causes. Finally, we multiplied the parent cause mortality rate (on a per draw basis) by 1 minus this cause fraction before raking the child causes.

To rake the county and racial–ethnic group estimates to the newly raked county-level estimates, first one-dimensional raking was applied to fit the county and racial–ethnic group all-cause mortality estimates to the raked county-level all-cause mortality estimates. Second, two-dimensional raking (across racial–ethnic group and cause) was applied at each level of the cause hierarchy.

### Impact of misclassification adjustment and calibration

Both misclassification adjustment and model calibration play important roles in creating more accurate mortality estimates. First, misclassification adjustment accounts for under-reporting or over-reporting of certain racial–ethnic groups on death certificates. However, because this adjustment is done independently for each racial–ethnic group, it can change the overall level of mortality estimated for a given county. The primary purpose of our calibration procedure is to constrain the adjusted estimates such that the overall level of mortality implied by the racial–ethnic group-specific estimates is the same as when estimating at the county level. Because these two processes are linked, we show here the cumulative impact of both adjustment for misclassification and calibration for all-cause mortality.

The figure in section 4.4 demonstrates the impact of these calculations on all-cause mortality at the national level. The estimate of total mortality is not impacted by calibration, as all other estimates are raked to these mortality rates. The estimates for each racial–ethnic group are impacted by both adjustment for misclassification and the calibration procedure. The combined effect is small for the White and Black populations, as expected given the small size of the misclassification adjustments for these two groups. There are moderate increases in mortality for the Asian and Latino populations, as well as increases in the uncertainty of those estimates, reflecting the somewhat larger misclassification adjustments as well as increased uncertainty from these adjustments. These processes have the largest effect on mortality among the AIAN population: there are large increases in mortality once adjusted and calibrated in addition to a large increase in uncertainty.

The maps in section 4.5 show the corresponding impact at the county level. Again, for the total population, there are no changes due to calibration or adjustment. The more substantial changes in mortality are noted at the national level for the AIAN, Asian, and Latino populations are again noted here, with changes at the county level generally in the same direction (ie, increases in mortality). Perhaps counterintuitively, there are instances where for a particular county and racial–ethnic group, the change in mortality after misclassification adjustment and calibration is not in the same direction as at the national level. This is caused by the interaction between adjustment and calibration. For example, in Bethel Census Area, Alaska, misclassification adjustment alone caused an increase in mortality, but calibration caused mortality to decrease because, in this county, the unadjusted estimates aggregated across racial–ethnic groups were higher than the estimated mortality rates produced by the county-level model. Another example is Bennett County, South Dakota, where age-standardised mortality for the AIAN population is lower once adjusted and calibrated. In this case, this is not due to the unadjusted, aggregated results specific to racial–ethnic group being higher than the estimates modelled with the county-level model in this county. Instead, this is due to misclassification adjustment. This county—along with many of the other counties with unmasked estimates in South Dakota—is a Contract Health Service Delivery Area (CHSDA), and therefore has a lower misclassification adjustment. This adjustment is 1.17, and the denominator is 1.4 (the total AIAN misclassification ratio for both Latino and non-Latino AIAN). Thus, the ratios of ratios is less than 1. This is combined with the region misclassification ratio, which is 1.12 and has a denominator of 1.33 (the total non-Latino AIAN misclassification ratio). Thus, the contribution from the region misclassification ratio is also less than 1. This means that the combined misclassification ratios are less than 1 for most ages and sexes in this county, thus leading to lower mortality. In contrast, Brown County, South Dakota exhibits increases in mortality due to raking and calibration. This is because Brown County is not a CHSDA, and thus its associated adjustment is larger than 1.

## 2.8 References

1 Dwyer-Lindgren L, Kendrick P, Kelly YO, *et al.* Life expectancy by county, race, and ethnicity in the USA, 2000–19: a systematic analysis of health disparities. *The Lancet* 2022; **0**. DOI:10.1016/S0140-6736(22)00876-5.

2 OMB DIRECTIVE 15: RACE AND ETHNIC STANDARDS FOR FEDERAL STATISTICS AND ADMINISTRATIVE REPORTING. https://wonder.cdc.gov/wonder/help/populations/bridged-race/directive15.html (accessed July 6, 2022).

3 Office of Management and Budget. Revisions to the Standards for the Classification of Federal Data on Race and Ethnicity. *Fed Regist* 1997; **62**: 58782–90.

4 Kochanek KD, Murphy SL, Xu J, Arias E. Deaths: final data for 2017. *Natl Vital Stat Rep* 2019; **68**. https://www.cdc.gov/nchs/data/nvsr/nvsr68/nvsr68_09-508.pdf (accessed Dec 23, 2019).

5 Arias E, Heron M, National Center for Health Statistics, Hakes J, US Census Bureau. The Validity of Race and Hispanic-origin Reporting on Death Certificates in the United States: An Update. *Vital Health Stat 2* 2016; : 1–21.

6 Naghavi M, Makela S, Foreman K, O’Brien J, Pourmalek F, Lozano R. Algorithms for enhancing public health utility of national causes-of-death data. *Popul Health Metr* 2010; **8**: 9.

7 Vos T, Lim SS, Abbafati C, *et al.* Global burden of 369 diseases and injuries in 204 countries and territories, 1990–2019: a systematic analysis for the Global Burden of Disease Study 2019. *The Lancet* 2020; **396**: 1204–22.

8 US Census Bureau. Instructions for applying statistical testing to American Community Survey data. 2019. https://www2.census.gov/programs-surveys/acs/tech_docs/statistical_testing/2019_Instructions_for_Stat_Testing_ACS.pdf (accessed Dec 17, 2020).

9 US Census Bureau CHS. Overview - History - U.S. Census Bureau. https://www.census.gov/history/www/through_the_decades/overview/ (accessed July 1, 2022).

10 Comparative Study of Confidence Intervals for Proportions in Complex Sample Surveys - PubMed. https://pubmed-ncbi-nlm-nih-gov.offcampus.lib.washington.edu/31428658/ (accessed July 1, 2022).

11 The arcsine is asinine: the analysis of proportions in ecology - Warton - 2011 - Ecology - Wiley Online Library. https://esajournals.onlinelibrary.wiley.com/doi/10.1890/10-0340.1 (accessed July 1, 2022).

12 Rue H, Martino S, Chopin N. Approximate Bayesian inference for latent Gaussian models by using integrated nested Laplace approximations. *J R Stat Soc Ser B Stat Methodol* 2009; **71**: 319–92.

13 R Core Team. R: A language and environment for statistical computing. 2019. https://www.R-project.org/.

14 Fuglstad G-A, Simpson D, Lindgren F, Rue H. Constructing Priors that Penalize the Complexity of Gaussian Random Fields. 2017; published online Nov 27. DOI:10.48550/arXiv.1503.00256.

15 Estimation of Disease Rates in Small Areas: A new Mixed Model for Spatial Dependence | SpringerLink. https://link.springer.com/chapter/10.1007/978-1-4612-1284-3_4 (accessed July 1, 2022).

16 Markov Chain Monte Carlo in Practice. Routledge CRC Press. https://www.routledge.com/Markov-Chain-Monte-Carlo-in-Practice/Gilks-Richardson-Spiegelhalter/p/book/9780412055515 (accessed July 1, 2022).

17 Knorr-Held L. Bayesian modelling of inseparable space-time variation in disease risk. *Stat Med* 2000; **19**: 2555–67.

18 Kristensen K, Nielsen A, Berg CW, Skaug H, Bell BM. TMB: Automatic Differentiation and Laplace Approximation. *J Stat Softw* 2016; **70**: 1–21.

19 Osgood-Zimmerman A, Wakefield J. A Statistical Introduction to Template Model Builder: A Flexible Tool for Spatial Modeling. 2021; published online March 17. DOI:10.48550/arXiv.2103.09929.

20 Bayesian Data Analysis. Routledge CRC Press. https://www.routledge.com/Bayesian-Data-Analysis/Gelman-Carlin-Stern-Dunson-Vehtari-Rubin/p/book/9781439840955 (accessed July 1, 2022).

21 Srebotnjak T, Mokdad AH, Murray CJ. A novel framework for validating and applying standardized small area measurement strategies. *Popul Health Metr* 2010; **8**: 26.

22 Dankovchik J, Hoopes MJ, Warren-Mears V, Knaster E. Disparities in Life Expectancy of Pacific Northwest American Indians and Alaska Natives: Analysis of Linkage-Corrected Life Tables. *Public Health Rep* 2015; **130**: 71–80.

23 Joshi S, Weiser T, Warren-Mears V. Drug, Opioid-Involved, and Heroin-Involved Overdose Deaths Among American Indians and Alaska Natives--Washington, 1999-2015. *Morb Mortal Wkly Rep* 2018; **67**: 1384–8.

24 Arias E, National Center for Health Statistics (U.S.), editors. The validity of race and Hispanic origin reporting on death certificates in the United States: data evaluation and methods research. Hyattsville, MD: U.S. Department of Health and Human Services/Centers for Disease Control and Prevention/National Center for Health Statistics, 2008.

25 Dwyer-Lindgren L, Bertozzi-Villa A, Stubbs RW, *et al.* US County-Level Trends in Mortality Rates for Major Causes of Death, 1980-2014. *JAMA* 2016; **316**: 2385–401.

26 Fienberg SE. An Iterative Procedure for Estimation in Contingency Tables. *Ann Math Stat* 1970; **41**: 907–17.

# 3 Supplemental Methods Tables

## 3.1 Counties combined to create historically stable units of analysis

| **State** | **Group** | **Counties (FIPS)** |
| --- | --- | --- |
| Alaska | 1 | Chugach Census Area (2063), Copper River Census Area (2066), Valdez-Cordova Census Area (2261)* |
| Alaska | 2 | Kusilvak Census Area (2158), Wade Hampton Census Area (2270)* |
|  | 3 | Kobuk Census Area (2140)*, Northwest Arctic Borough (2188) |
|  | 4 | Aleutian Islands Census Area (2010)*, Aleutians East Borough (2013), Aleutians West Census Area (2016) |
|  | 5 | Dillingham Census Area (2070), Lake and Peninsula Borough (2164) |
|  | 6 | Denali Borough (2068), Yukon-Koyukuk Census Area (2290) |
|  | 7 | Hoonah-Angoon Census Area (2105), Skagway Municipality (2230), Skagway-Yakutat-Angoon Census Area (2231)*, Skagway-Hoonah-Angoon Census Area (2232)*, Yakutat City and Borough (2282) |
|  | 8 | Ketchikan Gateway Borough (2130), Petersburg Borough (2195), Prince of Wales-Hyder Census Area (2198), Prince of Wales-Outer Ketchikan Census Area (2201)*, Wrangell City and Borough (2275), Wrangell-Petersburg Census Area (2280)* |
| Arizona | 1 | La Paz County (4012), Yuma County (4027) |
| Colorado | 1 | Adams County (8001), Arapahoe County (8005), Boulder County (8013), Broomfield County (8014), Denver County (8031), Jefferson County (8059), Weld County (8123) |
| Florida | 1 | Dade County (12025)*, Miami-Dade County (12086) |
| Hawaii | 1 | Kalawao County (15005), Maui County (15009) |
| Maryland | 1 | Montgomery County (24031), Prince George's County (24033) |
| Montana | 1 | Park County (30067), Yellowstone National Park (30113)* |
| New Mexico | 1 | Cibola County (35006), Valencia County (35061) |
| South Dakota | 1 | Oglala Lakota County (46102), Shannon County (46113)* |
|  | 2 | Jackson County (46071), Washabaugh County (46131)* |
| Virginia | 1 | Fairfax County (51059), Fairfax City (51600) |
|  | 2 | Rockingham County (51165), Harrisonburg City (51660) |
|  | 3 | James City County (51095), Williamsburg City (51830) |
|  | 4 | Prince William County (51153), Manassas City (51683), Manassas Park City (51685) |
|  | 5 | Rockbridge County (51163), Buena Vista City (51530) |
|  | 6 | Spotsylvania County (51177), Fredericksburg City (51630) |
|  | 7 | Augusta County (51015), Staunton City (51790), Waynesboro City (51820) |
|  | 8 | Pittsylvania County (51143), Danville City (51590) |
|  | 9 | Greensville County (51081), Emporia City (51595) |
|  | 10 | Albemarle County (51003), Charlottesville City (51540) |
|  | 11 | Bedford County (51019), Bedford City (51515)* |
|  | 12 | Halifax County (51083), South Boston City (51780)* |
|  | 13 | Southampton County (51175), Franklin City (51620) |
|  | 14 | Alleghany County (51005), Clifton Forge City (51560)* |
|  | 15 | York County (51199), Newport News City (51700) |

*County no longer exists due to boundary or name change.

## 3.2 Deaths and population data sources

| **Data type** | **Data source and citation** |
| --- | --- |
| Deaths | National Center for Health Statistics. National Vital Statistics System: Mortality Multiple Cause – All Counties Files, 2000-2019. Hyattsville, MD: National Center for Health Statistics, 2021. <https://www.cdc.gov/nchs/nvss/nvss-restricted-data.htm> |
| Population | National Center for Health Statistics, Centers for Disease Control and Prevention, US Census Bureau. United States Bridged-Race Intercensal Population Estimates 2000-2009. Hyattsville, United States: National Center for Health Statistics, Centers for Disease Control and Prevention, 2012. <https://www.cdc.gov/nchs/nvss/bridged_race.htm>. Accessed October 30, 2012.  National Center for Health Statistics, Centers for Disease Control and Prevention, US Census Bureau. United States Vintage 2020 Bridged-Race Postcensal Population Estimates 2010-2020. Hyattsville, United States: National Center for Health Statistics, Centers for Disease Control and Prevention, 2020. [https://www.cdc.gov/nchs/nvss/bridged_race.htm. Accessed February 17](https://www.cdc.gov/nchs/nvss/bridged_race.htm.%20Accessed%20February%2017), 2022. |

## 3.3 GBD cause list and associated ICD-10 codes

| **Cause Level** | **Cause** | **ICD-10 Codes** |
| --- | --- | --- |
| 1 | Communicable, maternal, neonatal, and nutritional diseases | A00–A00.9, A01.0–A14, A15–A28.9, A32–A39.9, A48.1–A48.2, A48.4–A48.5, A50–A58, A60–A60.9, A63–A63.8, A65–A65.0, A68–A70, A74, A74.8–A75.9, A77–A96.9, A98–A98.8, B00–B06.9, B10–B10.8, B15–B16.2, B17.0, B17.2, B19.1, B20–B27.9, B29.4, B33–B33.1, B33.3–B33.8, B34.2, B47–B48.8, B50–B53.8, B55.0, B56–B57.5, B60–B60.8, B63, B65–B67.9, B69–B72.0, B74.3–B75, B77–B77.9, B83–B83.8, B90–B91, B94.1, B95–B95.5, B97.2, B97.4–B97.6, C58–C58.0, D50.1–D50.8, D51–D52.0, D52.8–D53.9, D70.3, D89.3, E00–E02, E40–E46.9, E51–E61.9, E63–E64.0, E64.2–E64.9, F02.1, F02.4, F07.1, G00.0–G00.8, G03–G03.8, G04–G05.8, G14–G14.6, G21.3, H70–H70.9, I00, I02, I02.9, I98.0–I98.1, J00–J02.8, J03–J03.8, J04–J04.2, J05–J05.1, J06.0–J06.8, J09–J15.8, J16–J16.9, J20–J21.9, J36–J36.0, J91.0, K52.1–K52.3, K67.0–K67.8, K75.3, K76.3, K77.0, K93.0–K93.1, M03.1, M12.1, M49.0–M49.1, M73.0–M73.1, M89.6, N74.1, N96, N98–N98.9, O00–O07.9, O09–O16.9, O20–O26.9, O28–O36.9, O40–O48.1, O60–O77.9, O80–O92.7, O96–O98.6, O98.8–P04.2, P04.5–P05.9, P07–P15.9, P19–P22.9, P23.0–P23.4, P24–P29.9, P35–P37.2, P37.5–P39.9, P50–P61.9, P70–P70.1, P70.3–P72.9, P74–P78.9, P80–P81.9, P83–P84, P90–P92.9, P94–P94.9, P96, P96.3–P96.4, P96.8, R19.7, U04–U04.9, U06–U07.2, U82–U89, Z16–Z16.3 |
| 2 | HIV/AIDS and sexually transmitted infections | A50–A58, A60–A60.9, A63–A63.8, B20–B24.9, B63, F02.4, I98.0, K67.0–K67.2, M03.1, M73.0–M73.1 |
| 3 | HIV/AIDS | B20–B24.9, F02.4 |
| 4 | HIV/AIDS–Drug-susceptible Tuberculosis | B20.0 |
| 4 | HIV/AIDS resulting in other diseases | B20, B20.1–B24.9, F02.4 |
| 3 | Sexually transmitted infections excluding HIV | A50–A58, A60–A60.9, A63–A63.8, B63, I98.0, K67.0–K67.2, M03.1, M73.0–M73.1 |
| 4 | Syphilis | A50–A53.9, I98.0, K67.2, M03.1, M73.1 |
| 4 | Chlamydial infection | A55–A56.8, K67.0 |
| 4 | Gonorrhoea | A54–A54.9, K67.1, M73.0 |
| 4 | Other sexually transmitted infections | A57–A58, A63–A63.8, B63 |
| 2 | Respiratory infections and tuberculosis | A10–A14, A15–A19.9, A48.1, A70, B34.2, B90–B90.9, B97.2, B97.4–B97.6, H70–H70.9, J00–J02.8, J03–J03.8, J04–J04.2, J05–J05.1, J06.0–J06.8, J09–J15.8, J16–J16.9, J20–J21.9, J36–J36.0, J91.0, K67.3, K93.0, M49.0, N74.1, P23.0–P23.4, P37.0, U04–U04.9, U07–U07.2, U84.3 |
| 3 | Tuberculosis | A10–A14, A15–A19.9, B90–B90.9, K67.3, K93.0, M49.0, N74.1, P37.0, U84.3 |
| 4 | Drug-susceptible tuberculosis | A10–A14, A15–A19.9, B90–B90.9, K67.3, K93.0, M49.0, N74.1, P37.0 |
| 4 | Multidrug-resistant tuberculosis without extensive drug resistance | U84.3 |
| 3 | Lower respiratory infections | A48.1, A70, B34.2, B97.2, B97.4-B97.6, J09–J15.8, J16–J16.9, J20–J21.9, J91.0, P23.0–P23.4, U04–U04.9 |
| 4 | Influenza | J09–J11.8, U04–U04.9 |
| 4 | Pneumococcal pneumonia | J13–J13.9, J15.3–J15.4, J15.6 |
| 4 | H influenzae type B pneumonia | J14–J14.0 |
| 4 | Respiratory syncytial virus pneumonia | B97.4–B97.6, J12.1 |
| 4 | Other lower respiratory infections | A48.1, A70, B34.2, B97.2, J12–J12.0, J12.2–J12.9, J15–J15.2, J15.5, J15.7–J15.8, J16–J16.9, J20–J21.9, J91.0, P23.0–P23.4 |
| 3 | Upper respiratory infections | J00–J02.8, J03–J03.8, J04–J04.2, J05–J05.1, J06.0–J06.8, J36–J36.0 |
| 3 | Otitis media | H70–H70.9 |
| 3 | COVID-19 | U07–U07.2 |
| 2 | Enteric infections | A00–A00.9, A01.0–A09.9, A80–A80.9, K52.1–K52.3, R19.7 |
| 3 | Diarrhoeal diseases | A00–A00.9, A02–A02.0, A02.8–A07, A07.2–A07.4, A08–A09.9, K52.1–K52.3, R19.7 |
| 3 | Typhoid and paratyphoid | A01.0–A01.4 |
| 3 | Leprosy | A30–A30.9 |
| 4 | Typhoid fever | A01.0 |
| 4 | Paratyphoid fever | A01.1–A01.4 |
| 3 | Invasive Non-typhoidal Salmonella (iNTS) | A02.1–A02.2 |
| 3 | Other intestinal infectious diseases | A07.0–A07.1, A07.8–A07.9, A80–A80.9 |
| 2 | Neglected tropical diseases and malaria | A68–A68.9, A69.2–A69.9, A75–A75.9, A77–A79.9, A82–A82.9, A90–A96.9, A98–A98.8, B33.0–B33.1, B50–B53.8, B55.0, B56–B57.5, B60–B60.8, B65–B67.9, B69–B72.0, B74.3–B75, B77–B77.9, B83–B83.8, K93.1, P37.1, U06–U06.9 |
| 3 | Malaria | B50–B53.8 |
| 3 | Chagas disease | B57–B57.5, K93.1 |
| 3 | Leishmaniasis | B55.0 |
| 4 | Visceral leishmaniasis | B55.0 |
| 3 | African trypanosomiasis | B56–B56.9 |
| 3 | Schistosomiasis | B65–B65.9 |
| 3 | Cysticercosis | B69–B69.9 |
| 3 | Cystic echinococcosis | B67–B67.4, B67.8–B67.9 |
| 3 | Dengue | A90–A91.9 |
| 3 | Yellow fever | A95–A95.9 |
| 3 | Rabies | A82–A82.9 |
| 3 | Intestinal nematode infections | B77–B77.9 |
| 4 | Ascariasis | B77–B77.9 |
| 3 | Ebola | A98.4 |
| 3 | Zika virus | U06–U06.9 |
| 3 | Other neglected tropical diseases | A68–A68.9, A69.2–A69.9, A75–A75.9, A77–A79.9, A92–A94.0, A96–A96.9, A98–A98.3, A98.5–A98.8, B33.0–B33.1, B60–B60.8, B67.5–B67.7, B70–B71.9, B74.3–B75, B83–B83.8, P37.1 |
| 2 | Other infectious diseases | A20–A28.9, A32–A39.9, A48.2, A48.4–A48.5, A65–A65.0, A69–A69.1, A74, A74.8–A74.9, A81–A81.9, A83–A89.9, B00–B06.9, B10–B10.8, B15–B16.2, B17.0, B17.2, B19.1, B25–B27.9, B29.4, B33, B33.3–B33.8, B47–B48.8, B91, B94.1, B95–B95.5, D70.3, D89.3, F02.1, F07.1, G00.0–G00.8, G03–G03.8, G04–G05.8, G14–G14.6, G21.3, I00, I02, I02.9, I98.1, K67.8, K75.3, K76.3, K77.0, M49.1, M89.6, P35–P35.9, P37, P37.2, P37.5–P37.9, U82–U84, U85–U89, Z16–Z16.3 |
| 3 | Meningitis | A39–A39.9, A87–A87.9, G00.0–G00.8, G03–G03.8 |
| 3 | Encephalitis | A83–A86.4, B94.1, F07.1, G04–G05.8, G21.3 |
| 3 | Diphtheria | A36–A36.9 |
| 3 | Pertussis | A37–A37.9 |
| 3 | Tetanus | A33–A35.0 |
| 3 | Measles | B05–B05.9 |
| 3 | Varicella and herpes zoster | B01–B02.9, P35.8 |
| 3 | Acute hepatitis | B15–B16.2, B17.0, B17.2, B19.1, P35.3 |
| 4 | Acute hepatitis A | B15–B15.9 |
| 4 | Acute hepatitis B | B16–B16.2, B17.0, B19.1, P35.3 |
| 4 | Acute hepatitis E | B17.2 |
| 3 | Other unspecified infectious diseases | A20–A28.9, A32–A32.9, A38–A38.9, A48.2, A48.4–A48.5, A65–A65.0, A69–A69.1, A74, A74.8–A74.9, A81–A81.9, A88–A89.9, B00–B00.9, B03–B04, B06–B06.9, B10–B10.8, B25–B27.9, B29.4, B33, B33.3–B33.8, B47–B48.8, B91, B95–B95.5, D70.3, D89.3, F02.1, G14–G14.6, I00, I02, I02.9, I98.1, K67.8, K75.3, K76.3, K77.0, M49.1, M89.6, P35–P35.2, P35.9, P37, P37.2, P37.5–P37.9, U82–U84, U85–U89, Z16–Z16.3 |
| 2 | Maternal and neonatal disorders | C58–C58.0, N96, N98–N98.9, O00–O07.9, O09–O16.9, O20–O26.9, O28–O36.9, O40–O48.1, O60–O77.9, O80–O92.7, O96–O98.6, O98.8–P04.2, P04.5–P05.9, P07–P15.9, P19–P22.9, P24–P29.9, P36–P36.9, P38–P39.9, P50–P61.9, P70–P70.1, P70.3–P72.9, P74–P78.9, P80–P81.9, P83–P84, P90–P92.9, P94–P94.9, P96, P96.3–P96.4, P96.8 |
| 3 | Maternal disorders | C58–C58.0, N96, N98–N98.9, O00–O07.9, O09–O16.9, O20–O26.9, O28–O36.9, O40–O48.1, O60–O77.9, O80–O92.7, O96–O98.6, O98.8–O99.9 |
| 4 | Maternal haemorrhage | O20–O20.9, O43.2, O44–O46.9, O62–O62.9, O67–O67.9, O70, O72–O72.3 |
| 4 | Maternal sepsis and other maternal infections | O23–O23.9, O85–O86.8, O91–O91.2 |
| 4 | Maternal hypertensive disorders | O10–O16.9 |
| 4 | Maternal obstructed labour and uterine rupture | O32–O33.9, O64–O66.9, O71–O71.9 |
| 4 | Maternal abortion and miscarriage | N96, O01–O07.9 |
| 4 | Ectopic pregnancy | O00–O00.9 |
| 4 | Indirect maternal deaths | O24–O25.3, O98–O98.6, O98.8–O99.9 |
| 4 | Late maternal deaths | O96–O97.9 |
| 4 | Other direct maternal disorders | C58–C58.0, N98–N98.9, O09–O09.9, O21–O22.9, O26–O26.9, O28–O31.8, O34–O36.9, O40–O43.1, O43.8–O43.9, O47–O48.1, O60–O61.9, O63–O63.9, O68–O69.9, O70.0–O70.9, O73–O77.9, O80–O84.9, O87–O90.9, O92–O92.7 |
| 3 | Neonatal disorders | P00–P04.2, P04.5–P05.9, P07–P15.9, P19–P22.9, P24–P29.9, P36–P36.9, P38–P39.9, P50–P61.9, P70–P70.1, P70.3–P72.9, P74–P78.9, P80–P81.9, P83–P84, P90–P92.9, P94–P94.9, P96, P96.3–P96.4, P96.8 |
| 4 | Neonatal preterm birth | P01.0–P01.1, P05–P05.9, P07–P07.3, P22–P22.9, P25–P28.9, P52–P52.9, P61.2, P77–P77.9, P78.0–P78.9 |
| 4 | Neonatal encephalopathy due to birth asphyxia and trauma | P01.7, P02–P03.9, P10–P15.9, P20–P21.9, P24–P24.9, P90–P91.9 |
| 4 | Neonatal sepsis and other neonatal infections | P36–P36.9, P38–P39.9 |
| 4 | Haemolytic disease and other neonatal jaundice | P55–P59.9 |
| 4 | Other neonatal disorders | P00–P01, P01.2–P01.6, P01.8–P01.9, P04–P04.2, P04.5–P04.9, P08–P09, P19–P19.9, P29–P29.9, P50–P51.9, P53–P54.9, P60–P61.1, P61.3–P61.9, P70–P70.1, P70.3–P72.9, P74–P76.9, P78, P80–P81.9, P83–P84, P92–P92.9, P94–P94.9, P96, P96.3–P96.4, P96.8 |
| 2 | Nutritional deficiencies | D50.1–D50.8, D51–D52.0, D52.8–D53.9, E00–E02, E40–E46.9, E51–E61.9, E63–E64.0, E64.2–E64.9, M12.1 |
| 3 | Protein-energy malnutrition | E40–E46.9, E64.0 |
| 3 | Other nutritional deficiencies | D51–D52.0, D52.8–D53.9, E00–E02, E51–E61.9, E63–E64, E64.2–E64.9, M12.1 |
| 1 | Non-communicable diseases | A46–A46.0, A66–A67.9, B18–B18.9, B33.2, B86, C00–C13.9, C15–C22.8, C23–C25.9, C30–C34.9, C37–C38.8, C40–C41.9, C43–C45.9, C47–C54.9, C56–C57.8, C60–C63.8, C64–C67.9, C68.0–C68.8, C69.0–C69.8, C70–C73.9, C75–C75.8, C81–C82.9, C83.0–C83.8, C84–C85.0, C85.2–C85.8, C86–C86.6, C88–C91.0, C91.2–C91.3, C91.6, C92–C92.6, C93–C93.1, C93.3, C93.8, C94–C94.5, C94.7–C96.9, D00.1–D00.2, D01.0–D01.3, D02.0–D02.3, D03–D06.9, D07.0–D07.2, D07.4–D07.5, D09.0, D09.2–D09.3, D09.8, D10.0–D10.7, D11–D12.9, D13.0–D13.7, D14.0–D14.3, D15–D16.9, D22–D27.9, D28.0–D28.7, D29.0–D29.8, D30.0–D30.8, D31–D36, D36.1–D36.7, D37.1–D37.5, D38.0–D38.5, D39.1–D39.2, D39.8, D40.0–D40.8, D41.0–D41.8, D42–D43.9, D44.0–D44.8, D45–D47.9, D48.0–D48.6, D49.2–D49.4, D49.6, D55–D58.9, D59.1, D59.3, D59.5, D60–D61.9, D63.1, D64.0, D66–D67, D68.0–D69.4, D69.6–D69.8, D70–D70.0, D70.4–D75.8, D76–D77, D86–D86.9, D89–D89.2, E03–E03.1, E03.3–E06.3, E06.5–E07.1, E10–E11.9, E16.1–E16.9, E20–E23.0, E23.2–E24.1, E24.3–E27.2, E27.4–E34, E34.1–E34.8, E65–E66.0, E66.2–E68, E70–E85.2, E88–E88.2, E88.4–E88.9, F00–F02.0, F02.2–F02.3, F02.8–F03.9, F10–F16.9, F18–F18.9, F24, F50.0–F50.5, G10–G13.8, G20–G20.9, G23–G24, G24.1–G25.0, G25.2–G25.3, G25.5, G25.8–G26.0, G30–G31.9, G35–G37.9, G40–G41.9, G45–G46.8, G47.3, G61–G61.9, G62.1, G70–G72, G72.1–G73.7, G90–G90.9, G95–G95.9, H05.0–H05.1, I01–I01.9, I02.0, I05–I09.9, I11–I13.9, I20–I25.9, I27.0–I27.2, I28–I28.9, I30–I31.1, I31.8–I37.8, I38–I41.9, I42.1–I42.8, I43–I43.9, I47–I48.9, I51.0–I51.4, I60–I63.9, I65–I66.9, I67.0–I67.3, I67.5–I67.7, I68.0–I68.2, I69.0–I69.3, I70.2–I70.8, I71–I73.9, I77–I89.9, I98, I98.2, J30–J35.9, J37–J39.9, J41–J46.9, J60–J63.8, J66–J68.9, J70, J70.8–J70.9, J82, J84–J84.9, J91, J91.8–J92.9, K20–K20.9, K22–K22.6, K22.8–K29.9, K31–K31.8, K35–K38.9, K40–K42.9, K44–K46.9, K50–K52, K52.8–K52.9, K55–K62.6, K62.8–K62.9, K63.5, K64–K64.9, K66.8, K67, K68, K70–K70.3, K71.7, K73–K75, K75.1–K75.2, K75.4–K76.2, K76.4–K77, K77.8, K80–K83.9, K85–K86.9, K90–K90.9, K92.8, K93.8, L00–L05.9, L08–L08.9, L10–L14.0, L51–L51.9, L88–L89.9, L93–L93.2, L97–L98.4, M00–M03.0, M03.2–M03.6, M05–M09.8, M30–M36.8, M40–M43.1, M65–M65.0, M71.0–M71.1, M72.5–M72.6, M80–M82.8, M86.3–M86.4, M87–M87.0, M88–M89.0, M89.5, M89.7–M89.9, N00–N08.8, N10–N12.9, N13.6, N15–N16.8, N18–N18.9, N20–N23.0, N25–N28.1, N29–N30.3, N30.8–N32.0, N32.3–N32.4, N34–N34.3, N36–N36.9, N39–N39.2, N41–N41.9, N44–N44.0, N45–N45.9, N49–N49.9, N60–N60.9, N72–N72.0, N75–N77.8, N80–N81.9, N83–N83.9, N84.0–N84.1, N87–N87.9, P04.3–P04.4, P70.2, P96.0–P96.1, Q00–Q07.9, Q10.4–Q18.9, Q20–Q28.9, Q30–Q36, Q37–Q45.9, Q50–Q87.8, Q89–Q89.8, Q90–Q93.9, Q95–Q99.8, R78.0–R78.5, R95–R95.9, X45–X45.9, X65–X65.9, Y15–Y15.9 |
| 2 | Neoplasms | C00–C13.9, C15–C22.8, C23–C25.9, C30–C34.9, C37–C38.8, C40–C41.9, C43–C45.9, C47–C54.9, C56–C57.8, C60–C63.8, C64–C67.9, C68.0–C68.8, C69.0–C69.8, C70–C73.9, C75–C75.8, C81–C82.9, C83.0–C83.8, C84–C85.0, C85.2–C85.8, C86–C86.6, C88–C91.0, C91.2–C91.3, C91.6, C92–C92.6, C93–C93.1, C93.3, C93.8, C94–C94.5, C94.7–C96.9, D00.1–D00.2, D01.0–D01.3, D02.0–D02.3, D03–D06.9, D07.0–D07.2, D07.4–D07.5, D09.0, D09.2–D09.3, D09.8, D10.0–D10.7, D11–D12.9, D13.0–D13.7, D14.0–D14.3, D15–D16.9, D22–D24.9, D26.0–D27.9, D28.0–D28.1, D28.7, D29.0–D29.8, D30.0–D30.8, D31–D36, D36.1–D36.7, D37.1–D37.5, D38.0–D38.5, D39.1–D39.2, D39.8, D40.0–D40.8, D41.0–D41.8, D42–D43.9, D44.0–D44.8, D45–D47.9, D48.0–D48.6, D49.2–D49.4, D49.6, K62.0–K62.1, K63.5, N60–N60.9, N84.0–N84.1, N87–N87.9 |
| 3 | Lip and oral cavity cancer | C00–C08.9, D10.0–D10.5, D11–D11.9 |
| 3 | Nasopharynx cancer | C11–C11.9, D10.6 |
| 3 | Other pharynx cancer | C09–C10.9, C12–C13.9, D10.7 |
| 3 | Oesophageal cancer | C15–C15.9, D00.1, D13.0 |
| 3 | Stomach cancer | C16–C16.9, D00.2, D13.1, D37.1 |
| 3 | Colon and rectum cancer | C18–C21.9, D01.0–D01.3, D12–D12.9, D37.3–D37.5 |
| 3 | Liver cancer | C22–C22.8, D13.4 |
| 4 | Hepatoblastoma | C22.2 |
| 3 | Gallbladder and biliary tract cancer | C23–C24.9, D13.5 |
| 3 | Pancreatic cancer | C25–C25.9, D13.6–D13.7 |
| 3 | Larynx cancer | C32–C32.9, D02.0, D14.1, D38.0 |
| 3 | Tracheal, bronchus, and lung cancer | C33–C34.9, D02.1–D02.3, D14.2–D14.3, D38.1 |
| 3 | Malignant skin melanoma | C43–C43.9, D03–D03.9, D22–D23.9, D48.5 |
| 3 | Non-melanoma skin cancer | C44–C44.9, D04–D04.9, D49.2 |
| 4 | Non-melanoma skin cancer (squamous-cell carcinoma) | C44–C44.9, D04–D04.9, D49.2 |
| 3 | Soft tissue and other extraosseous sarcomas | C49–C49.9 |
| 3 | Malignant neoplasm of bone and articular cartilage | C40–C41.9 |
| 3 | Breast cancer | C50–C50.9, D05–D05.9, D24–D24.9, D48.6, D49.3 |
| 3 | Cervical cancer | C53–C53.9, D06–D06.9, D26.0 |
| 3 | Uterine cancer | C54–C54.9, D07.0–D07.2, D26.1–D26.9 |
| 3 | Ovarian cancer | C56–C56.9, D27–D27.9, D39.1 |
| 3 | Prostate cancer | C61–C61.9, D07.5, D29.1, D40.0 |
| 3 | Testicular cancer | C62–C62.9, D29.2–D29.8, D40.1–D40.8 |
| 3 | Kidney cancer | C64–C65.9, D30.0–D30.1, D41.0–D41.1 |
| 3 | Bladder cancer | C67–C67.9, D09.0, D30.3, D41.4–D41.8, D49.4 |
| 3 | Brain and central nervous system cancer | C70–C72.9, C75.1–C75.3 |
| 3 | Eye cancer | C69.0–C69.8 |
| 4 | Retinoblastoma | C69.2 |
| 4 | Other eye cancers | C69.0–C69.1, C69.3–C69.8 |
| 3 | Neuroblastoma and other peripheral nervous cell tumours | C47–C47.9 |
| 3 | Thyroid cancer | C73–C73.9, D09.3, D09.8, D34–D34.9, D44.0 |
| 3 | Mesothelioma | C45–C45.9 |
| 3 | Hodgkin lymphoma | C81–C81.9 |
| 3 | Non-Hodgkin lymphoma | C82–C82.9, C83.0–C83.8, C84–C85.0, C85.2–C85.8, C86–C86.6, C96–C96.9 |
| 4 | Burkitt lymphoma | C83.7 |
| 4 | Other non-Hodgkin lymphoma | C82–C82.9, C83.0–C83.6, C83.8, C84–C85.0, C85.2–C85.8, C86–C86.6, C96–C96.9 |
| 3 | Multiple myeloma | C88–C90.9 |
| 3 | Leukaemia | C91–C91.0, C91.2–C91.3, C91.6, C92–C92.6, C93–C93.1, C93.3, C93.8, C94–C94.5, C94.7–C95.9 |
| 4 | Acute lymphoid leukaemia | C91.0, C91.2–C91.3, C91.6 |
| 4 | Acute myeloid leukaemia | C92.0, C92.3–C92.6, C93.0, C94.0, C94.2, C94.4–C94.5 |
| 4 | Chronic myeloid leukaemia | C92.1–C92.2 |
| 4 | Other leukaemia | C93.1, C93.3, C93.8, C94.1, C94.3, C94.7–C94.8 |
| 3 | Other malignant neoplasms | C17–C17.9, C30–C31.9, C37–C38.8, C48–C48.9, C4A, C51–C52.9, C57–C57.8, C60–C60.9, C63–C63.8, C66–C66.9, C68.0–C68.8, C75–C75.0, C75.4–C75.8, D07.4, D09.2, D13.2–D13.3, D14.0, D15–D16.9, D28.0–D28.1, D28.7, D29.0, D30.2, D30.4–D30.8, D31–D31.9, D35–D35.2, D35.5–D36, D36.1–D36.7, D37.2, D38.2–D38.5, D39.2, D39.8, D41.2–D41.3, D44.1–D44.8, D48.0–D48.4 |
| 3 | Other neoplasms | D32–D33.9, D35.3–D35.4, D42–D43.9, D45–D47.9, D49.6, K62.0–K62.1, K63.5, N60–N60.9, N84.0–N84.1, N87–N87.9 |
| 4 | Myelodysplastic, myeloproliferative, and other haematopoietic neoplasms | D45–D47.9 |
| 4 | Other benign and in situ neoplasms | N60–N60.9 |
| 2 | Cardiovascular diseases | B33.2, G45–G46.8, I01–I01.9, I02.0, I05–I09.9, I11–I11.9, I20–I25.9, I27.0, I27.2, I28–I28.9, I30–I31.1, I31.8–I37.8, I38–I41.9, I42.1–I42.8, I43–I43.9, I47–I48.9, I51.0–I51.4, I60–I63.9, I65–I66.9, I67.0–I67.3, I67.5–I67.6, I68.0–I68.2, I69.0–I69.3, I70.2–I70.8, I71–I73.9, I77–I83.9, I86–I89.0, I89.9, I98, K75.1 |
| 3 | Rheumatic heart disease | I01–I01.9, I02.0, I05–I09.9 |
| 3 | Ischaemic heart disease | I20–I25.9 |
| 3 | Stroke | G45–G46.8, I60–I63.9, I65–I66.9, I67.0–I67.3, I67.5–I67.6, I68.1–I68.2, I69.0–I69.3 |
| 4 | Ischaemic stroke | G45–G46.8, I63–I63.9, I65–I66.9, I67.2–I67.3, I67.5–I67.6, I69.3 |
| 4 | Intracerebral haemorrhage | I61–I62, I62.1–I62.9, I68.1–I68.2, I69.1–I69.2 |
| 4 | Subarachnoid haemorrhage | I60–I60.9, I62.0, I67.0–I67.1, I69.0 |
| 3 | Hypertensive heart disease | I11–I11.9 |
| 3 | Non-rheumatic valvular heart disease | I34–I37.8 |
| 4 | Non-rheumatic calcific aortic valve disease | I35–I35.9 |
| 4 | Non-rheumatic degenerative mitral valve disease | I34–I34.9 |
| 4 | Other non-rheumatic valve diseases | I36–I37.8 |
| 3 | Cardiomyopathy and myocarditis | B33.2, I40–I41.9, I42.1–I42.8, I43–I43.9, I51.4 |
| 4 | Myocarditis | B33.2, I40–I41.9, I51.4 |
| 4 | Alcoholic cardiomyopathy | I42.6 |
| 4 | Other cardiomyopathy | I42.1–I42.5, I42.7–I42.8, I43–I43.9 |
| 3 | Pulmonary Arterial Hypertension | I27.0 |
| 3 | Atrial fibrillation and flutter | I48–I48.9 |
| 3 | Aortic aneurysm | I71–I71.9 |
| 3 | Lower extremity peripheral arterial disease | I70.2–I70.8, I73–I73.9 |
| 3 | Endocarditis | I33–I33.9, I38–I39.9 |
| 3 | Other cardiovascular and circulatory diseases | I27.2, I28–I28.9, I30–I31.1, I31.8–I32.8, I47–I47.9, I51.0–I51.3, I68.0, I72–I72.9, I77–I83.9, I86–I89.0, I89.9, I98, K75.1 |
| 2 | Chronic respiratory diseases | D86–D86.2, D86.9, G47.3, J30–J35.9, J37–J39.9, J41–J46.9, J60–J63.8, J66–J68.9, J70, J70.8–J70.9, J82, J84–J84.9, J91, J91.8–J92.9 |
| 3 | Chronic obstructive pulmonary disease | J41–J44.9 |
| 3 | Pneumoconiosis | J60–J63.8, J92.0 |
| 4 | Silicosis | J62–J62.9 |
| 4 | Asbestosis | J61–J61.0, J92.0 |
| 4 | Coal workers pneumoconiosis | J60–J60.0 |
| 4 | Other pneumoconiosis | J63–J63.8 |
| 3 | Asthma | J45–J46.9 |
| 3 | Interstitial lung disease and pulmonary sarcoidosis | D86–D86.2, D86.9, J84–J84.9 |
| 3 | Other chronic respiratory diseases | G47.3, J30–J35.9, J37–J39.9, J66–J68.9, J70, J70.8–J70.9, J82, J91, J91.8–J92, J92.9 |
| 2 | Digestive diseases | B18–B18.9, I84–I85.9, I98.2, K20–K20.9, K22–K22.6, K22.8–K29.9, K31–K31.8, K35–K38.9, K40–K42.9, K44–K46.9, K50–K52, K52.8–K52.9, K55–K62, K62.2–K62.6, K62.8–K62.9, K64–K64.9, K66.8, K67, K68, K70–K70.3, K71.7, K73–K75, K75.2, K75.4–K76.2, K76.4–K77, K77.8, K80–K83.9, K85–K86.9, K90–K90.9, K92.8, K93.8, M09.1 |
| 3 | Cirrhosis and other chronic liver diseases | B18–B18.9, I85–I85.9, I98.2, K70–K70.3, K71.7, K73–K75, K75.2, K75.4–K76.2, K76.4–K76.9, K77.8 |
| 3 | Upper digestive system diseases | K25–K29.9 |
| 4 | Peptic ulcer disease | K25–K28.9 |
| 4 | Gastritis and duodenitis | K29–K29.9 |
| 3 | Appendicitis | K35–K37.9, K38.3–K38.9 |
| 3 | Paralytic ileus and intestinal obstruction | K56–K56.9 |
| 3 | Inguinal, femoral, and abdominal hernia | K40–K42.9, K44–K46.9 |
| 3 | Inflammatory bowel disease | K50–K52, K52.8–K52.9, M09.1 |
| 4 | Ulcerative colitis | K51–K52, K52.8–K52.9 |
| 4 | Crohn's disease | K50–K50.9, M09.1 |
| 3 | Vascular intestinal disorders | K55–K55.9 |
| 3 | Gallbladder and biliary diseases | K80–K83.9 |
| 3 | Pancreatitis | K85–K86.9 |
| 3 | Other digestive diseases | I84–I84.9, K20–K20.9, K22–K22.6, K22.8–K24, K31–K31.8, K38–K38.2, K57–K62, K62.2–K62.6, K62.8–K62.9, K64–K64.9, K66.8, K67, K68, K77, K90–K90.9, K92.8, K93.8 |
| 2 | Neurological disorders | F00–F02.0, F02.2–F02.3, F02.8–F03.9, G10–G13.8, G20–G20.9, G23–G24, G24.1–G25.0, G25.2–G25.3, G25.5, G25.8–G26.0, G30–G31.1, G31.8–G31.9, G35–G37.9, G40–G41.9, G61–G61.9, G70–G71.1, G71.3–G72, G72.2–G73.7, G90–G90.9, G95–G95.9, M33–M33.9 |
| 3 | Alzheimer's disease and other dementias | F00–F02.0, F02.8–F03.9, G30–G31.1, G31.8–G31.9 |
| 3 | Parkinson's disease | F02.3, G20–G20.9 |
| 3 | Idiopathic epilepsy | G40–G41.9 |
| 3 | Multiple sclerosis | G35–G35.9 |
| 3 | Motor neuron disease | G12.2–G12.9 |
| 3 | Other neurological disorders | F02.2, G10–G12.1, G13–G13.8, G23–G24, G24.1–G25.0, G25.2–G25.3, G25.5, G25.8–G26.0, G36–G37.9, G61–G61.9, G70–G71.1, G71.3–G72, G72.2–G73.7, G90–G90.9, G95–G95.9, M33–M33.9 |
| 2 | Mental disorders | F24, F50.0–F50.5 |
| 3 | Eating disorders | F50.0–F50.5 |
| 4 | Anorexia nervosa | F50.0–F50.5 |
| 2 | Substance use disorders | E24.4, F10–F16.9, F18–F18.9, G31.2, G62.1, G72.1, P04.3–P04.4, P96.1, Q86.0, R78.0–R78.5, X45–X45.9, X65–X65.9, Y15–Y15.9 |
| 3 | Alcohol use disorders | E24.4, F10–F10.9, G31.2, G62.1, G72.1, P04.3, Q86.0, R78.0, X45–X45.9, X65–X65.9, Y15–Y15.9 |
| 3 | Drug use disorders | F11–F16.9, F18–F18.9, P04.4, P96.1, R78.1–R78.5 |
| 4 | Opioid use disorders | F11–F11.9, P96.1, R78.1 |
| 4 | Cocaine use disorders | F14–F14.9, R78.2 |
| 4 | Amphetamine use disorders | F15–F15.9 |
| 4 | Other drug use disorders | F13–F13.9, F16–F16.9, F18–F18.9, P04.4, R78.3–R78.5 |
| 2 | Diabetes and kidney diseases | D63.1, E10–E11.9, I12–I13.9, N00–N08.8, N15.0, N18–N18.9, P70.2, Q61–Q62.8 |
| 3 | Diabetes mellitus | E10–E10.1, E10.3–E11.1, E11.3–E11.9, P70.2 |
| 4 | Diabetes mellitus type 1 | E10–E10.1, E10.3–E10.9, P70.2 |
| 4 | Diabetes mellitus type 2 | E11–E11.1, E11.3–E11.9 |
| 3 | Chronic kidney disease | D63.1, E10.2, E11.2, I12–I13.9, N02–N08.8, N15.0, N18–N18.9, Q61–Q62.8 |
| 4 | Chronic kidney disease due to diabetes mellitus type 1 | E10.2 |
| 4 | Chronic kidney disease due to diabetes mellitus type 2 | E11.2 |
| 4 | Chronic kidney disease due to hypertension | I12–I13.9 |
| 4 | Chronic kidney disease due to glomerulonephritis | N03–N06.9 |
| 4 | Chronic kidney disease due to other and unspecified causes | N02–N02.9, N07–N08.8, N15.0, Q61–Q62.8 |
| 3 | Acute glomerulonephritis | N00–N01.9 |
| 2 | Skin and subcutaneous diseases | A46–A46.0, A66–A67.9, B86, D86.3, H05.0–H05.1, I89.1–I89.8, L00–L05.9, L08–L08.9, L10–L14.0, L51–L51.9, L88–L89.9, L97–L98.4, M72.5–M72.6 |
| 3 | Bacterial skin diseases | A46–A46.0, A66–A67.9, H05.0–H05.1, I89.1–I89.8, L00–L05.9, L08–L08.9, L88, L97–L98.4, M72.5–M72.6 |
| 4 | Cellulitis | H05.0, L03–L03.9, M72.5–M72.6 |
| 4 | Pyoderma | A46–A46.0, A66–A67.9, H05.1, I89.1–I89.8, L00–L02.9, L04–L05.9, L08–L08.9, L88, L97–L98.4 |
| 3 | Decubitus ulcer | L89–L89.9 |
| 3 | Other skin and subcutaneous diseases | D86.3, L10–L14.0, L51–L51.9 |
| 2 | Musculoskeletal disorders | I27.1, I67.7, L93–L93.2, M00–M03.0, M03.2–M03.6, M05–M09.0, M09.2–M09.8, M30–M32.9, M34–M36.8, M40–M43.1, M65–M65.0, M71.0–M71.1, M80–M82.8, M86.3–M86.4, M87–M87.0, M88–M89.0, M89.5, M89.7–M89.9 |
| 3 | Rheumatoid arthritis | M05–M06.9, M08.0–M08.8 |
| 3 | Other musculoskeletal disorders | I27.1, I67.7, L93–L93.2, M00–M03.0, M03.2–M03.6, M07–M08, M08.9–M09.0, M09.2–M09.8, M30–M32.9, M34–M36.8, M40–M43.1, M65–M65.0, M71.0–M71.1, M80–M82.8, M86.3–M86.4, M87–M87.0, M88–M89.0, M89.5, M89.7–M89.9 |
| 2 | Other non-communicable diseases | D25–D26, D28.2, D55–D58.9, D59.1, D59.3, D59.5, D60–D61.9, D64.0, D66–D67, D68.0–D69.4, D69.6–D69.8, D70–D70.0, D70.4–D75.8, D76–D77, D86.8, D89–D89.2, E03–E03.1, E03.3–E06.3, E06.5–E07.1, E16.1–E16.9, E20–E23.0, E23.2–E24.1, E24.3, E24.8–E27.2, E27.4–E34, E34.1–E34.8, E65–E66.0, E66.2–E68, E70–E85.2, E88–E88.2, E88.4–E88.9, G71.2, N10–N12.9, N13.6, N15, N15.1–N16.8, N20–N23.0, N25–N28.1, N29–N30.3, N30.8–N32.0, N32.3–N32.4, N34–N34.3, N36–N36.9, N39–N39.2, N41–N41.9, N44–N44.0, N45–N45.9, N49–N49.9, N72–N72.0, N75–N77.8, N80–N81.9, N83–N83.9, P96.0, Q00–Q07.9, Q10.4–Q18.9, Q20–Q28.9, Q30–Q36, Q37–Q45.9, Q50–Q60.6, Q63–Q86, Q86.1–Q87.8, Q89–Q89.8, Q90–Q93.9, Q95–Q99.8, R95–R95.9 |
| 3 | Congenital birth defects | G71.2, P96.0, Q00–Q07.9, Q10.4–Q18.9, Q20–Q28.9, Q30–Q36, Q37–Q45.9, Q50–Q60.6, Q63–Q86, Q86.1–Q87.8, Q89–Q89.8, Q90–Q93.9, Q95–Q99.8 |
| 4 | Neural tube defects | Q00–Q01.9, Q05–Q05.9 |
| 4 | Congenital heart anomalies | Q20–Q28.9 |
| 4 | Orofacial clefts | Q35–Q36, Q37–Q37.9 |
| 4 | Down syndrome | Q90–Q90.9 |
| 4 | Other chromosomal abnormalities | Q87–Q87.8, Q91–Q93.9, Q95–Q95.9, Q97–Q97.9, Q99–Q99.8 |
| 4 | Congenital musculoskeletal and limb anomalies | Q65–Q79, Q79.6–Q79.9 |
| 4 | Urogenital congenital anomalies | P96.0, Q50–Q60.6, Q63–Q64.9 |
| 4 | Digestive congenital anomalies | Q38–Q45.9, Q79.0–Q79.5 |
| 4 | Other congenital birth defects | G71.2, Q02–Q04.9, Q06–Q07.9, Q10.4–Q18.9, Q30–Q34.9, Q80–Q86, Q86.1–Q86.8, Q89–Q89.8 |
| 3 | Urinary diseases and male infertility | N10–N12.9, N13.6, N15, N15.1–N16.8, N20–N23.0, N25–N28.1, N29–N30.3, N30.8–N32.0, N32.3–N32.4, N34–N34.3, N36–N36.9, N39–N39.2, N41–N41.9, N44–N44.0, N45–N45.9, N49–N49.9 |
| 4 | Urinary tract infections and interstitial nephritis | N10–N12.9, N13.6, N15, N15.1–N16.8, N30–N30.3, N30.8–N30.9, N34–N34.3, N39.0–N39.2 |
| 4 | Urolithiasis | N20–N23.0 |
| 4 | Other urinary diseases | N25–N28.1, N29–N29.8, N31–N32.0, N32.3–N32.4, N36–N36.9, N39, N41–N41.9, N44–N44.0, N45–N45.9, N49–N49.9 |
| 3 | Gynaecological diseases | D25–D26, D28.2, E28.2, N72–N72.0, N75–N77.8, N80–N81.9, N83–N83.9 |
| 4 | Uterine fibroids | D25–D26, D28.2 |
| 4 | Endometriosis | N80–N80.9 |
| 4 | Genital prolapse | N81–N81.9 |
| 4 | Other gynaecological diseases | N72–N72.0, N75–N77.8, N83–N83.9 |
| 3 | Haemoglobinopathies and haemolytic anaemias | D55–D58.9, D59.1, D59.3, D59.5, D60–D61.9, D64.0 |
| 4 | Thalassaemias | D56–D56.9 |
| 4 | Sickle cell disorders | D57–D57.8 |
| 4 | G6PD deficiency | D55–D55.2 |
| 4 | Other haemoglobinopathies and haemolytic anaemias | D55.3–D55.9, D58–D58.9, D59.1, D59.3, D59.5, D60–D61.9, D64.0 |
| 3 | Endocrine, metabolic, blood, and immune disorders | D66–D67, D68.0–D69.4, D69.6–D69.8, D70–D70.0, D70.4–D75.8, D76–D77, D86.8, D89–D89.2, E03–E03.1, E03.3–E06.3, E06.5–E07.1, E16.1–E16.9, E20–E23.0, E23.2–E24.1, E24.3, E24.8–E27.2, E27.4–E28.1, E28.3–E34, E34.1–E34.8, E65–E66.0, E66.2–E68, E70–E85.2, E88–E88.2, E88.4–E88.9 |
| 4 | Thyroid diseases | E03–E03.1, E03.3–E06.3, E06.5–E07, E07.1 |
| 4 | Other endocrine, metabolic, blood, and immune disorders | D66–D67, D68.0–D69.4, D69.6–D69.8, D70–D70.0, D70.4–D75.8, D76–D77, D86.8, D89–D89.2, E07.0, E16.1–E16.9, E20–E23.0, E23.2–E24.1, E24.3, E24.8–E27.2, E27.4–E28.1, E28.3–E34, E34.1–E34.8, E67–E68, E70–E77.9, E79–E83.9, E85–E85.2, E88–E88.2, E88.4–E88.9 |
| 3 | Sudden infant death syndrome | R95–R95.9 |
| 1 | Injuries | D52.1, D59.0, D59.2, D59.6, D69.5, D70.1–D70.2, D78–D78.8, E03.2, E06.4, E09–E09.9, E16.0, E23.1, E24.2, E27.3, E36–E36.8, E66.1, E88.3, E89–E89.9, G21.0–G21.1, G24.0, G25.1, G25.4, G25.6–G25.7, G72.0, G93.7, G97–G97.9, I95.2–I95.3, I97–I97.9, I98.9, J70.0–J70.5, J95–J95.9, K43–K43.9, K52.0, K62.7, K91–K91.9, K94–K95.8, L55–L55.9, L56.3, L56.8–L56.9, L58–L58.9, M87.1, N14–N14.4, N30.4, N65–N65.1, N99–N99.9, P93–P93.8, P96.2, P96.5, R50.2, U00–U03, V00–V86.9, V87.2–V87.3, V88.2–V88.3, V90–V98.8, W00–W46.2, W49–W62.9, W64–W70.9, W73–W75.9, W77–W81.9, W83–W94.9, W97.9, W99–X06.9, X08–X39.9, X47–X48.9, X50–X54.9, X57–X58.9, X60–X64.9, X66–X83.9, X85–Y08.9, Y35–Y84.9, Y87.0–Y87.1, Y88–Y88.3, Y89.0–Y89.1 |
| 2 | Transport injuries | V00–V86.9, V87.2–V87.3, V88.2–V88.3, V90–V98.8 |
| 3 | Road injuries | V01–V04.9, V06–V80.9, V82–V82.9, V87.2–V87.3 |
| 4 | Pedestrian road injuries | V01–V04.9, V06–V09.9 |
| 4 | Cyclist road injuries | V10–V19.9 |
| 4 | Motorcyclist road injuries | V20–V29.9 |
| 4 | Motor vehicle road injuries | V30–V79.9, V87.2–V87.3 |
| 4 | Other road injuries | V80–V80.9, V82–V82.9 |
| 3 | Other transport injuries | V00–V00.8, V05–V05.9, V81–V81.9, V83–V86.9, V88.2–V88.3, V90–V98.8 |
| 2 | Unintentional injuries | D52.1, D59.0, D59.2, D59.6, D69.5, D70.1–D70.2, D78–D78.8, E03.2, E06.4, E09–E09.9, E16.0, E23.1, E24.2, E27.3, E36–E36.8, E66.1, E88.3, E89–E89.9, G21.0–G21.1, G24.0, G25.1, G25.4, G25.6–G25.7, G72.0, G93.7, G97–G97.9, I95.2–I95.3, I97–I97.9, I98.9, J70.0–J70.5, J95–J95.9, K43–K43.9, K52.0, K62.7, K91–K91.9, K94–K95.8, L55–L55.9, L56.3, L56.8–L56.9, L58–L58.9, M87.1, N14–N14.4, N30.4, N65–N65.1, N99–N99.9, P93–P93.8, P96.2, P96.5, R50.2, W00–W46.2, W49–W62.9, W64–W70.9, W73–W75.9, W77–W81.9, W83–W94.9, W97.9, W99–X06.9, X08–X39.9, X47–X48.9, X50–X54.9, X57–X58.9, Y40–Y84.9, Y88–Y88.3 |
| 3 | Falls | W00–W19.9 |
| 3 | Drowning | W65–W70.9, W73–W74.9 |
| 3 | Fire, heat, and hot substances | X00–X06.9, X08–X19.9 |
| 3 | Poisonings | X47–X48.9 |
| 4 | Poisoning by carbon monoxide | X47–X47.9 |
| 4 | Poisoning by other means | X48–X48.9 |
| 3 | Exposure to mechanical forces | W20–W38.9, W40–W43.9, W45.0–W45.2, W46–W46.2, W49–W52 |
| 4 | Unintentional firearm injuries | W32–W34.9 |
| 4 | Other exposure to mechanical forces | W20–W31.9, W35–W38.9, W40–W43.9, W45.0–W45.2, W46–W46.2, W49–W52 |
| 3 | Adverse effects of medical treatment | D52.1, D59.0, D59.2, D59.6, D69.5, D70.1–D70.2, D78–D78.8, E03.2, E06.4, E09–E09.9, E16.0, E23.1, E24.2, E27.3, E36–E36.8, E66.1, E88.3, E89–E89.9, G21.0–G21.1, G24.0, G25.1, G25.4, G25.6–G25.7, G72.0, G93.7, G97–G97.9, I95.2–I95.3, I97–I97.9, I98.9, J70.0–J70.5, J95–J95.9, K43–K43.9, K52.0, K62.7, K91–K91.9, K94–K95.8, M87.1, N14–N14.4, N30.4, N65–N65.1, N99–N99.9, P93–P93.8, P96.2, P96.5, R50.2, Y40–Y84.9, Y88–Y88.3 |
| 3 | Animal contact | W52.0–W62.9, W64–W64.9, X20–X29.9 |
| 4 | Venomous animal contact | X20–X29.9 |
| 4 | Non-venomous animal contact | W52.0–W62.9, W64–W64.9 |
| 3 | Foreign body | W44–W45, W45.3–W45.9, W75–W75.9, W78–W80.9, W83–W84.9 |
| 4 | Pulmonary aspiration and foreign body in airway | W75–W75.9, W78–W80.9, W83–W84.9 |
| 4 | Foreign body in other body part | W44–W45, W45.3–W45.9 |
| 3 | Environmental heat and cold exposure | L55–L55.9, L56.3, L56.8–L56.9, L58–L58.9, W88–W94.9, W97.9, W99–W99.9, X30–X32.9, X39–X39.9 |
| 3 | Exposure to forces of nature | X33–X38.9 |
| 3 | Other unintentional injuries | W39–W39.9, W77–W77.9, W81–W81.9, W85–W87.9, X50–X54.9, X57–X58.9 |
| 2 | Self-harm and interpersonal violence | U00–U03, X60–X64.9, X66–X83.9, X85–Y08.9, Y35–Y38.9, Y87.0–Y87.1, Y89.0–Y89.1 |
| 3 | Self-harm | X60–X64.9, X66–X83.9, Y87.0 |
| 4 | Self-harm by firearm | X72–X74.9 |
| 4 | Self-harm by other specified means | X60–X64.9, X66–X71.9, X75–X83.9, Y87.0 |
| 3 | Interpersonal violence | X85–Y08.9, Y87.1 |
| 4 | Physical violence by firearm | X93–X95.9 |
| 4 | Physical violence by sharp object | X99–X99.9 |
| 4 | Physical violence by other means | X85–X92.9, X96–X98.9, Y00–Y04.9, Y06–Y08.9, Y87.1 |
| 3 | Conflict and terrorism | U00–U03, Y36–Y38.9, Y89.1 |
| 3 | Police conflict and executions | Y35–Y35.9, Y89.0 |
|  |  |  |

## 3.4 Covariate data sources

| **Covariate** | **Data sources** | **Data processing** | **Citations** |
| --- | --- | --- | --- |
| Household median income | 2000-2019 Small Area Income and Poverty Estimates [1]; 2000-2019 Bureau of Labor Statistics, Consumer Price Index [2] | Data were adjusted for inflation using the consumer price index. | [1] US Census Bureau. Small Area Income and Poverty Estimates. <https://www.census.gov/programs-surveys/saipe/data/datasets.html>. Accessed October 6, 2022.  [2] US Bureau of Labor Statistics. Consumer Price Index: All Urban Consumers History, All Items 1913-2021. <https://www.bls.gov/data/>. Accessed October 6, 2022. |
| Population density | 2000-2019 NCHS bridged race files [3-4]; 2020 cartographic boundary file, state county for United States [5] accessed using the tigris package [6] | The area of each county was calculated using an Albers Equal Area Conic projection. The total population of each county was divided by the total area of the county and was then log-transformed. | [3] National Center for Health Statistics, Centers for Disease Control and Prevention, US Census Bureau. United States Bridged-Race Intercensal Population Estimates 2000-2009. Hyattsville, United States: National Center for Health Statistics, Centers for Disease Control and Prevention, 2012. <https://www.cdc.gov/nchs/nvss/bridged_race.htm>. Accessed October 30, 2012.  [4] National Center for Health Statistics, Centers for Disease Control and Prevention, US Census Bureau. United States Vintage 2020 Bridged-Race Postcensal Population Estimates 2010-2020. Hyattsville, United States: National Center for Health Statistics, Centers for Disease Control and Prevention, 2020. <https://www.cdc.gov/nchs/nvss/bridged_race.htm>. Accessed February 17, 2022.  [5] US Census Bureau. TIGER/Line Shapefile, 2020 Cartographic Boundary File, State-County for United States, 1:20,000,000.  https://www.census.gov/geographies/mapping-files/time-series/geo/tiger-line-file.2020.html#list-tab-790442341. Accessed October 12, 2022.  [6] Walker K (2022). *tigris: Load Census TIGER/Line Shapefiles*. R package version 2.0, <https://github.com/walkerke/tigris>. |
| Percent of the population age 25 and older who have completed a bachelor’s degree by race and ethnicity | 1990 census [6]; 2000 census [7]; 2010-2020 ACS [8] | ACS estimates for Black, American Indian or Alaskan Native (AIAN), and Asian or Pacific Islander (Asian) were not available stratified by Latino ethnicity and were used as proxies for non-Latino Black, non-Latino AIAN, and non-Latino Asian estimates, respectively. Imputation via a small area estimation model was used to generate and smooth missing values. | [6] Minnesota Population Center. 1990 Census Summary Tape File 4, Table NPB44. IPUMS National Historical Geographic Information System: Version 15.0. Minneapolis, MN: IPUMS 2020. <https://www.nhgis.org/>. Accessed August 25, 2020.  [7] Minnesota Population Center. 2000 Census Summary File 4, Table NPCT064C. IPUMS National Historical Geographic Information System: Version 15.0. Minneapolis, MN: IPUMS 2020. <https://www.nhgis.org/>. Accessed August 25, 2020.  [8] US Census Bureau. American Community Survey, 2010-2020 American Community Survey 5-Year Estimates, Tables C15002A-C15002I; using Census data portal; <https://data.census.gov/cedsci/>. Accessed October 5, 2022. |
| Percent of the population below the poverty line by race and ethnicity | 1990 census [9]; 2000 census [10]; 2010-2020 ACS [11] | ACS estimates for Black, American Indian or Alaskan Native (AIAN), and Asian or Pacific Islander (Asian) were not available stratified by Latino ethnicity and were used as proxies for non-Latino Black, non-Latino AIAN, and non-Latino Asian estimates, respectively. Imputation via a small area estimation model was used to generate and smooth missing values. | [9] Minnesota Population Center. 1990 Census Summary Tape File 4, Table NPB100. IPUMS National Historical Geographic Information System: Version 15.0. Minneapolis, MN: IPUMS 2020. <https://www.nhgis.org/>. Accessed August 30, 2020.  [10] Minnesota Population Center. 2000 Census Summary File 4, Table NPCT142A. IPUMS National Historical Geographic Information System: Version 15.0. Minneapolis, MN: IPUMS 2020. <https://www.nhgis.org/>. Accessed August 30, 2020.  [11] US Census Bureau. American Community Survey, 2010-2020 American Community Survey 5-Year Estimates, Tables B17001A-B17001I; using Census data portal; <https://data.census.gov/cedsci/>. Accessed October 5, 2022. |
| Percent of the population that is foreign-born by race and ethnicity | 1990 census [12]; 2000 census [13]; 2010-2020 ACS [14] | ACS estimates for Black, American Indian or Alaskan Native (AIAN), and Asian or Pacific Islander (Asian) were not available stratified by Latino ethnicity and were used as proxies for non-Latino Black, non-Latino AIAN, and non-Latino Asian estimates, respectively. Imputation via a small area estimation model was used to generate and smooth missing values. | [12] Minnesota Population Center. 1990 Census Summary Tape File 4, Table NPB28. IPUMS National Historical Geographic Information System: Version 15.0. Minneapolis, MN: IPUMS 2020. <https://www.nhgis.org/>. Accessed October 8, 2020.  [13] Minnesota Population Center. 2000 Census Summary File 4, Table NPCT043A. IPUMS National Historical Geographic Information System: Version 15.0. Minneapolis, MN: IPUMS 2020. <https://www.nhgis.org/>. Accessed October 8, 2020.  [14] US Census Bureau. American Community Survey, 2010-2020 American Community Survey 5-Year Estimates, Tables B05003A-B05003I; using Census data portal; <https://data.census.gov/cedsci/>. Accessed October 5, 2022. |

## 3.5 Population mask

|  | **AIAN** | | | **Asian** | | | **Black** | | | **Latino** | | | **White** | | | **Total** | | |
| --- | --- | --- | --- | --- | --- | --- | --- | --- | --- | --- | --- | --- | --- | --- | --- | --- | --- | --- |
| **Strata** | **Counties masked** | **Person-years masked*** | **Percent person-**  **years in**  **strata** | **Counties masked** | **Person-years masked*** | **Percent person-**  **years in**  **strata** | **Counties masked** | **Person-years masked*** | **Percent person-**  **years in**  **strata** | **Counties masked** | **Person-years masked*** | **Percent person-**  **years in**  **strata** | **Counties masked** | **Person-years masked*** | **Percent person-**  **years in**  **strata** | **Counties masked** | **Person-years masked*** | **Percent person-**  **years in**  **strata** |
| **Total** | 2636 (84.8%) | 9.1 (17.8%) |  | 2443 (78.6%) | 8.5 (2.6%) |  | 1622 (52.2%) | 7.5 (1.0%) |  | 1632 (52.5%) | 11.4 (1.2%) |  | 59 (1.9%) | 0.8 (0.0%) |  | 31 (1.0%) | 0.4 (0.0%) |  |
| **Urban or rural code** | |  |  |  |  |  |  |  |  |  |  |  |  |  |  |  |  |  |
| *Large central metro* | 8 (10.1%) | 0.1 (0.7%) | 16.5% | 0 (0.0%) | 0.0 (0.0%) | 51.8% | 0 (0.0%) | 0.0 (0.0%) | 42.1% | 0 (0.0%) | 0.0 (0.0%) | 49.5% | 0 (0.0%) | 0.0 (0.0%) | 22.1% | 0 (0.0%) | 0.0 (0.0%) | 30.6% |
| *Large fringe metro* | 309 (78.0%) | 1.5 (28.7%) | 10.4% | 184 (46.5%) | 0.9 (1.1%) | 24.7% | 83 (21.0%) | 0.5 (0.3%) | 20.8% | 103 (26.0%) | 0.7 (0.4%) | 17.2% | 0 (0.0%) | 0.0 (0.0%) | 24.8% | 0 (0.0%) | 0.0 (0.0%) | 23.0% |
| *Medium metro* | 359 (73.6%) | 1.5 (14.9%) | 19.5% | 239 (49.0%) | 0.9 (1.7%) | 15.9% | 115 (23.6%) | 0.5 (0.4%) | 19.0% | 121 (24.8%) | 0.7 (0.4%) | 19.8% | 1 (0.2%) | 0.0 (0.0%) | 22.1% | 0 (0.0%) | 0.0 (0.0%) | 21.0% |
| *Small metro* | 356 (80.9%) | 1.3 (22.0%) | 11.6% | 241 (54.8%) | 1.0 (8.1%) | 3.8% | 125 (28.4%) | 0.5 (0.9%) | 6.9% | 149 (33.9%) | 0.8 (1.4%) | 5.9% | 2 (0.5%) | 0.0 (0.0%) | 10.4% | 1 (0.2%) | 0.0 (0.0%) | 8.9% |
| *Micropolitan* | 717 (87.5%) | 2.1 (21.4%) | 19.5% | 693 (84.6%) | 3.5 (37.0%) | 2.9% | 384 (46.9%) | 2.4 (4.9%) | 6.1% | 307 (37.5%) | 2.3 (4.8%) | 4.9% | 9 (1.1%) | 0.1 (0.0%) | 11.2% | 6 (0.7%) | 0.0 (0.0%) | 9.2% |
| *Noncore* | 1694 (92.7%) | 2.6 (22.5%) | 22.4% | 1805 (98.8%) | 2.2 (83.2%) | 0.8% | 1340 (73.3%) | 3.6 (9.1%) | 5.1% | 1399 (76.6%) | 6.8 (25.7%) | 2.7% | 59 (3.2%) | 0.7 (0.2%) | 9.4% | 31 (1.7%) | 0.4 (0.1%) | 7.4% |
| **Census region** |  |  |  |  |  |  |  |  |  |  |  |  |  |  |  |  |  |  |
| South | 1246 (88.6%) | 3.9 (23.5%) | 32.0% | 1137 (80.8%) | 4.0 (5.7%) | 21.6% | 451 (32.1%) | 2.8 (0.6%) | 56.1% | 686 (48.8%) | 5.4 (1.5%) | 35.9% | 19 (1.4%) | 0.2 (0.0%) | 34.8% | 8 (0.6%) | 0.1 (0.0%) | 37.0% |
| West | 269 (62.4%) | 1.3 (5.6%) | 44.3% | 309 (71.7%) | 1.2 (0.8%) | 46.5% | 319 (74.0%) | 1.2 (1.6%) | 9.2% | 152 (35.3%) | 0.9 (0.2%) | 40.7% | 18 (4.2%) | 0.3 (0.0%) | 19.5% | 9 (2.1%) | 0.1 (0.0%) | 23.2% |
| Northeast | 172 (79.3%) | 0.9 (30.6%) | 6.0% | 110 (50.7%) | 0.8 (1.3%) | 20.0% | 69 (31.8%) | 0.6 (0.4%) | 16.4% | 67 (30.9%) | 0.7 (0.5%) | 14.1% | 0 (0.0%) | 0.0 (0.0%) | 19.3% | 0 (0.0%) | 0.0 (0.0%) | 18.0% |
| Midwest | 949 (90.0%) | 3.0 (33.5%) | 17.7% | 887 (84.1%) | 2.5 (6.5%) | 11.9% | 783 (74.2%) | 3.0 (2.1%) | 18.4% | 727 (68.9%) | 4.4 (4.9%) | 9.2% | 22 (2.1%) | 0.3 (0.0%) | 26.4% | 14 (1.3%) | 0.2 (0.0%) | 21.8% |

*Person-years are given in millions.

Modelled age-standardised mortality estimates were masked (not displayed) in all years for county and racial–ethnic group combinations with a mean annual population of fewer than 1000 people. The number of unique counties masked (and corresponding percentage of all counties), the person-years in millions represented by these county-years (and corresponding percentage of all person-years), and the percentage of the person-years masked in each county and racial–ethnic group combination are listed in this table. AIAN = non-Latino and non-Hispanic American Indian or Alaska Native.

## 3.6 Hyperparameter posterior means and standard errors for stomach cancer model

| **Model** | **Cause** | **Sex** | **Parameter** |  | **Mean (SE)*** |
| --- | --- | --- | --- | --- | --- |
| By racial–ethnic group and county | Stomach cancer (Level 3) | Females | $\gamma_{1}$ | Standard deviation, Proportion with Bachelors Degree | 0·064 (SE = 0·045) |
| By racial–ethnic group and county | Stomach cancer (Level 3) | Females | $\gamma_{1}$ | Standard deviation, Proportion in poverty | 0·131 (SE = 0·056) |
| By racial–ethnic group and county | Stomach cancer (Level 3) | Females | $\gamma_{1}$ | Standard deviation, Proportion foreign born | 0·732 (SE = 0·288) |
| By racial–ethnic group and county | Stomach cancer (Level 3) | Females | $\gamma_{2}$ | Standard deviation | 0·049 (SE = 0·026) |
| By racial–ethnic group and county | Stomach cancer (Level 3) | Females | $\gamma_{2}$ | Correlation parameter, County | 0·806 (SE = 0·31) |
| By racial–ethnic group and county | Stomach cancer (Level 3) | Females | $\gamma_{3}$ | Standard deviation | 0·017 (SE = 0·002) |
| By racial–ethnic group and county | Stomach cancer (Level 3) | Females | $\gamma_{3}$ | Correlation parameter, Year | >0·999 (SE < 0·001) |
| By racial–ethnic group and county | Stomach cancer (Level 3) | Females | $\gamma_{3}$ | Correlation parameter, Age | 0·981 (SE = 0·005) |
| By racial–ethnic group and county | Stomach cancer (Level 3) | Females | $\gamma_{4}$ | Standard deviation | 0·049 (SE = 0·008) |
| By racial–ethnic group and county | Stomach cancer (Level 3) | Females | $\gamma_{4}$ | Correlation parameter, County | 0·997 (SE = 0·001) |
| By racial–ethnic group and county | Stomach cancer (Level 3) | Females | $\gamma_{4}$ | Correlation parameter, Year spline | 0·967 (SE = 0·01) |
| By racial–ethnic group and county | Stomach cancer (Level 3) | Females | $\gamma_{4}$ | Correlation parameter, Age spline | 0·756 (SE = 0·058) |
| By racial–ethnic group and county | Stomach cancer (Level 3) | Males | $\gamma_{1}$ | Standard deviation, Proportion with Bachelors Degree | 0·178 (SE = 0·087) |
| By racial–ethnic group and county | Stomach cancer (Level 3) | Males | $\gamma_{1}$ | Standard deviation, Proportion in poverty | 0·08 (SE = 0·038) |
| By racial–ethnic group and county | Stomach cancer (Level 3) | Males | $\gamma_{1}$ | Standard deviation, Proportion foreign born | 0·544 (SE = 0·22) |
| By racial–ethnic group and county | Stomach cancer (Level 3) | Males | $\gamma_{2}$ | Standard deviation | 0·098 (SE = 0·012) |
| By racial–ethnic group and county | Stomach cancer (Level 3) | Males | $\gamma_{2}$ | Correlation parameter, County | 0·98 (SE = 0·016) |
| By racial–ethnic group and county | Stomach cancer (Level 3) | Males | $\gamma_{3}$ | Standard deviation | 0·015 (SE = 0·002) |
| By racial–ethnic group and county | Stomach cancer (Level 3) | Males | $\gamma_{3}$ | Correlation parameter, Year | >0·999 (SE < 0·001) |
| By racial–ethnic group and county | Stomach cancer (Level 3) | Males | $\gamma_{3}$ | Correlation parameter, Age | 0·985 (SE = 0·004) |
| By racial–ethnic group and county | Stomach cancer (Level 3) | Males | $\gamma_{4}$ | Standard deviation | 0·052 (SE = 0·007) |
| By racial–ethnic group and county | Stomach cancer (Level 3) | Males | $\gamma_{4}$ | Correlation parameter, County | 0·998 (SE = 0·001) |
| By racial–ethnic group and county | Stomach cancer (Level 3) | Males | $\gamma_{4}$ | Correlation parameter, Year spline | 0·962 (SE = 0·01) |
| By racial–ethnic group and county | Stomach cancer (Level 3) | Males | $\gamma_{4}$ | Correlation parameter, Age spline | 0·808 (SE = 0·041) |
| By county | Stomach cancer (Level 3) | Females | $\gamma_{2}$ | Standard deviation | 0·15 (SE = 0·016) |
| By county | Stomach cancer (Level 3) | Females | $\gamma_{2}$ | Correlation parameter, County | 0·988 (SE = 0·009) |
| By county | Stomach cancer (Level 3) | Females | $\gamma_{3}$ | Standard deviation | 0·017 (SE = 0·003) |
| By county | Stomach cancer (Level 3) | Females | $\gamma_{3}$ | Correlation parameter, Year | >0·999 (SE < 0·001) |
| By county | Stomach cancer (Level 3) | Females | $\gamma_{3}$ | Correlation parameter, Age | 0·966 (SE = 0·015) |
| By county | Stomach cancer (Level 3) | Females | $\gamma_{4}$ | Standard deviation | 0·06 (SE = 0·011) |
| By county | Stomach cancer (Level 3) | Females | $\gamma_{4}$ | Correlation parameter, County | 0·994 (SE = 0·003) |
| By county | Stomach cancer (Level 3) | Females | $\gamma_{4}$ | Correlation parameter, Year spline | 0·965 (SE = 0·013) |
| By county | Stomach cancer (Level 3) | Females | $\gamma_{4}$ | Correlation parameter, Age spline | 0·482 (SE = 0·154) |
| By county | Stomach cancer (Level 3) | Males | $\gamma_{2}$ | Standard deviation | 0·149 (SE = 0·027) |
| By county | Stomach cancer (Level 3) | Males | $\gamma_{2}$ | Correlation parameter, County | 0·985 (SE = 0·014) |
| By county | Stomach cancer (Level 3) | Males | $\gamma_{3}$ | Standard deviation | 0·017 (SE = 0·003) |
| By county | Stomach cancer (Level 3) | Males | $\gamma_{3}$ | Correlation parameter, Year | >0·999 (SE < 0·001) |
| By county | Stomach cancer (Level 3) | Males | $\gamma_{3}$ | Correlation parameter, Age | 0·967 (SE = 0·015) |
| By county | Stomach cancer (Level 3) | Males | $\gamma_{4}$ | Standard deviation | 0·07 (SE = 0·012) |
| By county | Stomach cancer (Level 3) | Males | $\gamma_{4}$ | Correlation parameter, County | 0·997 (SE = 0·002) |
| By county | Stomach cancer (Level 3) | Males | $\gamma_{4}$ | Correlation parameter, Year spline | 0·948 (SE = 0·02) |
| By county | Stomach cancer (Level 3) | Males | $\gamma_{4}$ | Correlation parameter, Age spline | 0·662 (SE = 0·154) |

*Estimates are reported by TMB on the log standard deviation and logit correlation parameter scale. For convenience, we have transformed these using a delta transformation for the standard error. SE = standard error; TMB = Template Model Builder package in R version 3.6.110.

## 3.7 County and racial–ethnic groups in the all-cause mortality validation set

| State | Merged county | Racial–ethnic group* |
| --- | --- | --- |
| Alabama | Jefferson | All racial–ethnic groups, Black |
|  | Madison | All racial–ethnic groups |
|  | Mobile | All racial–ethnic groups, Black |
| Alaska | Anchorage Municipality | All racial–ethnic groups |
| Arizona | Maricopa | All racial–ethnic groups, Black, Latino, White |
|  | Pima | All racial–ethnic groups, Latino |
|  | Pinal | All racial–ethnic groups |
| Arkansas | Pulaski | All racial–ethnic groups |
| California | Alameda | All racial–ethnic groups, Latino |
|  | Contra Costa | All racial–ethnic groups, White |
|  | Fresno | All racial–ethnic groups, Latino |
|  | Kern | All racial–ethnic groups, Latino, White |
|  | Los Angeles | All racial–ethnic groups, Asian, Black, Latino, White |
|  | Merced | All racial–ethnic groups |
|  | Monterey | All racial–ethnic groups, Latino |
|  | Orange | All racial–ethnic groups, Latino, White |
|  | Riverside | All racial–ethnic groups, Latino, White |
|  | Sacramento | All racial–ethnic groups |
|  | San Bernardino | All racial–ethnic groups, Black, Latino |
|  | San Diego | All racial–ethnic groups, Latino, White |
|  | San Francisco | All racial–ethnic groups |
|  | San Joaquin | All racial–ethnic groups, Latino |
|  | San Mateo | All racial–ethnic groups |
|  | Santa Clara | All racial–ethnic groups, Asian, Latino |
|  | Solano | All racial–ethnic groups |
|  | Sonoma | All racial–ethnic groups |
|  | Stanislaus | All racial–ethnic groups |
|  | Tulare | All racial–ethnic groups, Latino |
|  | Ventura | All racial–ethnic groups |
| Colorado | Adams/Arapahoe/Boulder/Broomfield/Denver/Jefferson/Weld | All racial–ethnic groups, Latino, White |
|  | El Paso | All racial–ethnic groups |
| Connecticut | Fairfield | All racial–ethnic groups, White |
|  | Hartford | All racial–ethnic groups |
|  | New Haven | All racial–ethnic groups, White |
| District of Columbia | District of Columbia | All racial–ethnic groups, Black |
| Florida | Brevard | All racial–ethnic groups |
|  | Broward | All racial–ethnic groups, Black, White |
|  | Miami-Dade | All racial–ethnic groups, Black, Latino |
|  | Duval | All racial–ethnic groups, Black, White |
|  | Hillsborough | All racial–ethnic groups, Black, Latino, White |
|  | Lee | All racial–ethnic groups |
|  | Orange | All racial–ethnic groups |
|  | Palm Beach | All racial–ethnic groups, Latino, White |
|  | Pasco | All racial–ethnic groups |
|  | Pinellas | All racial–ethnic groups |
|  | Polk | All racial–ethnic groups |
|  | Seminole | All racial–ethnic groups |
| Georgia | Bibb | All racial–ethnic groups |
|  | Cobb | All racial–ethnic groups, White |
|  | DeKalb | All racial–ethnic groups, Black |
|  | Fulton | All racial–ethnic groups, Black, White |
|  | Gwinnett | All racial–ethnic groups |
| Hawaii | Honolulu | All racial–ethnic groups, Asian |
| Illinois | Cook | All racial–ethnic groups, Black, Latino, White |
|  | DuPage | All racial–ethnic groups |
|  | Kane | All racial–ethnic groups |
|  | Lake | All racial–ethnic groups |
|  | Will | All racial–ethnic groups, White |
| Indiana | Allen | All racial–ethnic groups |
|  | Elkhart | All racial–ethnic groups |
|  | Lake | All racial–ethnic groups, Black |
|  | Marion | All racial–ethnic groups, Black, White |
| Iowa | Polk | All racial–ethnic groups |
| Kansas | Sedgwick | All racial–ethnic groups, White |
| Kentucky | Jefferson | All racial–ethnic groups, White |
| Louisiana | Caddo | All racial–ethnic groups |
|  | East Baton Rouge | All racial–ethnic groups, Black |
|  | Jefferson | All racial–ethnic groups |
|  | Orleans | All racial–ethnic groups, Black |
| Maryland | Anne Arundel | All racial–ethnic groups |
|  | Baltimore | All racial–ethnic groups |
|  | Baltimore City | All racial–ethnic groups, Black |
|  | Montgomery/Prince George's | All racial–ethnic groups, Black |
| Massachusetts | Bristol | All racial–ethnic groups |
|  | Essex | All racial–ethnic groups |
|  | Hampden | All racial–ethnic groups |
|  | Middlesex | All racial–ethnic groups, White |
|  | Suffolk | All racial–ethnic groups |
|  | Worcester | All racial–ethnic groups |
| Michigan | Genesee | All racial–ethnic groups |
|  | Kent | All racial–ethnic groups |
|  | Macomb | All racial–ethnic groups, White |
|  | Oakland | All racial–ethnic groups |
|  | Wayne | All racial–ethnic groups, Black, White |
| Minnesota | Hennepin | All racial–ethnic groups, White |
|  | Ramsey | All racial–ethnic groups |
| Mississippi | Hinds | All racial–ethnic groups, Black |
| Missouri | Jackson | All racial–ethnic groups, White |
|  | Saint Louis | All racial–ethnic groups, Black, White |
| Nebraska | Douglas | All racial–ethnic groups |
| Nevada | Clark | All racial–ethnic groups, Latino, White |
|  | Washoe | All racial–ethnic groups |
| New Jersey | Bergen | All racial–ethnic groups, White |
|  | Burlington | All racial–ethnic groups |
|  | Camden | All racial–ethnic groups |
|  | Essex | All racial–ethnic groups, Black |
|  | Hudson | All racial–ethnic groups |
|  | Mercer | All racial–ethnic groups |
|  | Middlesex | All racial–ethnic groups |
|  | Monmouth | All racial–ethnic groups, White |
|  | Ocean | All racial–ethnic groups, White |
|  | Passaic | All racial–ethnic groups |
|  | Union | All racial–ethnic groups |
| New Mexico | Bernalillo | All racial–ethnic groups, Latino |
|  | Dona Ana | All racial–ethnic groups |
|  | San Juan | All racial–ethnic groups |
| New York | Bronx | All racial–ethnic groups, Black, Latino |
|  | Erie | All racial–ethnic groups, White |
|  | Kings | All racial–ethnic groups, Black, Latino, White |
|  | Monroe | All racial–ethnic groups, White |
|  | Nassau | All racial–ethnic groups, White |
|  | New York | All racial–ethnic groups, Latino |
|  | Onondaga | All racial–ethnic groups |
|  | Queens | All racial–ethnic groups, Asian, Black, Latino |
|  | Suffolk | All racial–ethnic groups, White |
|  | Westchester | All racial–ethnic groups, White |
| North Carolina | Durham | All racial–ethnic groups |
|  | Forsyth | All racial–ethnic groups |
|  | Mecklenburg | All racial–ethnic groups, White |
|  | Wake | All racial–ethnic groups, White |
| Ohio | Cuyahoga | All racial–ethnic groups, Black, White |
|  | Franklin | All racial–ethnic groups, Black |
|  | Hamilton | All racial–ethnic groups, Black, White |
|  | Lucas | All racial–ethnic groups |
|  | Montgomery | All racial–ethnic groups, White |
| Oklahoma | Oklahoma | All racial–ethnic groups, White |
|  | Tulsa | All racial–ethnic groups, White |
| Oregon | Multnomah | All racial–ethnic groups |
| Pennsylvania | Allegheny | All racial–ethnic groups, Black, White |
|  | Chester | All racial–ethnic groups, White |
|  | Delaware | All racial–ethnic groups, White |
|  | Lancaster | All racial–ethnic groups, White |
|  | Montgomery | All racial–ethnic groups, White |
|  | Philadelphia | All racial–ethnic groups, Black, Latino, White |
| Rhode Island | Providence | All racial–ethnic groups, White |
| South Carolina | Greenville | All racial–ethnic groups, White |
|  | Spartanburg | All racial–ethnic groups |
| Tennessee | Davidson | All racial–ethnic groups, Black |
|  | Knox | All racial–ethnic groups |
|  | Montgomery | All racial–ethnic groups |
|  | Shelby | All racial–ethnic groups, Black |
| Texas | Bell | All racial–ethnic groups |
|  | Bexar | All racial–ethnic groups, Latino, White |
|  | Cameron | All racial–ethnic groups, Latino |
|  | Collin | All racial–ethnic groups, White |
|  | Dallas | All racial–ethnic groups, Black, Latino, White |
|  | Denton | All racial–ethnic groups, White |
|  | El Paso | All racial–ethnic groups, Latino |
|  | Fort Bend | All racial–ethnic groups |
|  | Galveston | All racial–ethnic groups |
|  | Harris | All racial–ethnic groups, Black, Latino, White |
|  | Hidalgo | All racial–ethnic groups, Latino |
|  | Jefferson | All racial–ethnic groups |
|  | McLennan | All racial–ethnic groups |
|  | Montgomery | All racial–ethnic groups |
|  | Tarrant | All racial–ethnic groups, Latino, White |
|  | Travis | All racial–ethnic groups, White |
|  | Webb | All racial–ethnic groups, Latino |
| Utah | Salt Lake | All racial–ethnic groups, White |
|  | Utah | All racial–ethnic groups, White |
| Virginia | Chesapeake City | All racial–ethnic groups |
|  | Fairfax/Fairfax City | All racial–ethnic groups |
|  | Norfolk City | All racial–ethnic groups |
|  | York/Newport News City | All racial–ethnic groups |
| Washington | Clark | All racial–ethnic groups |
|  | King | All racial–ethnic groups, White |
|  | Pierce | All racial–ethnic groups, White |
|  | Spokane | All racial–ethnic groups |
|  | Yakima | All racial–ethnic groups |
| Wisconsin | Milwaukee | All racial–ethnic groups, Black, White |
|  | Rock | All racial–ethnic groups |

* “All racial–ethnic groups” was validated separately from individual racial–ethnic groups.

## 3.8 All-cause mortality model validation results

|  | **Model 1** | | | | **Model 2** | | | **Model 3** | | |
| --- | --- | --- | --- | --- | --- | --- | --- | --- | --- | --- |
| **Racial–ethnic group** | **Size** | **Mean**  **relative**  **error (%)** | **Mean**  **absolute**  **relative**  **error (%)** | **Coverage (%)** | **Mean**  **relative**  **error (%)** | **Mean**  **absolute**  **relative**  **error (%)** | **Coverage (%)** | **Mean**  **relative**  **error (%)** | **Mean**  **absolute**  **relative**  **error (%)** | **Coverage (%)** |
| Across all racial–ethnic groups | 10 | 1·15 | 6·73 | 99·03 | -0·1 | 6·61 | 99·72 | 0·07 | 6·65 | 99·41 |
|  | 100 | 1·13 | 6·7 | 98·83 | -0·02 | 6·85 | 99·62 | 0·2 | 6·74 | 99·18 |
|  | 1000 | 0·44 | 6 | 97·09 | 0·04 | 6·42 | 97·59 | -0·02 | 6·4 | 97·45 |
|  | 3000 | 0·69 | 5 | 95·94 | 0·43 | 5·29 | 96·23 | 0·46 | 5·27 | 96·16 |
|  | 5000 | 0·3 | 4·55 | 94·8 | 0·11 | 4·72 | 94·88 | 0·15 | 4·72 | 94·93 |
|  | 10 000 | 0·29 | 3·73 | 94·42 | 0·19 | 3·8 | 94·55 | 0·2 | 3·83 | 94·68 |
|  | 25 000 | 0·16 | 2·9 | 92·87 | 0·04 | 2·97 | 92·89 | 0·13 | 2·97 | 93·01 |
|  | 100 000 | -0·05 | 2·05 | 87·56 | -0·05 | 2·08 | 87·05 | -0·04 | 2·09 | 87·23 |
| Asian | 10 | 8·67 | 8·72 | 100 | 7·77 | 7·99 | 100 | 6·98 | 7·4 | 100 |
|  | 100 | 8·23 | 8·51 | 100 | 6·8 | 7·57 | 100 | 6·29 | 7·17 | 100 |
|  | 1000 | 5·33 | 6·2 | 98·24 | 5·54 | 7·51 | 98·53 | 4·42 | 6·42 | 98·38 |
|  | 3000 | 3·26 | 4·37 | 97·94 | 1·98 | 4·33 | 98·09 | 2·44 | 4·35 | 98·09 |
|  | 5000 | 2·66 | 4·9 | 97·79 | 1·79 | 5·51 | 98·09 | 2·04 | 5·16 | 98·68 |
|  | 10 000 | 1·71 | 3·99 | 96·62 | 1·1 | 4·15 | 96·91 | 1·43 | 4·18 | 96·32 |
|  | 25 000 | 0·59 | 3·13 | 96·32 | 0·15 | 3·13 | 96·91 | 0·42 | 3·14 | 96·32 |
|  | 100 000 | 0·07 | 2·28 | 91·18 | 0·1 | 2·35 | 90·74 | 0·15 | 2·35 | 90·59 |
| Black | 10 | 2·9 | 7·32 | 99·17 | 0·58 | 6·63 | 99·95 | 1·25 | 7·29 | 99·64 |
|  | 100 | 2·81 | 7·31 | 99·2 | 0·72 | 6·98 | 99·77 | 1·32 | 7·53 | 99·56 |
|  | 1000 | 1·31 | 6·31 | 96·54 | 0·46 | 6·43 | 96·8 | 0·48 | 6·5 | 96·67 |
|  | 3000 | 0·86 | 5·27 | 95·57 | 0·24 | 5·29 | 95·52 | 0·32 | 5·29 | 96·08 |
|  | 5000 | 0·56 | 4·54 | 94·53 | 0·11 | 4·48 | 94·62 | 0·2 | 4·49 | 95·11 |
|  | 10 000 | 0·56 | 3·87 | 93·69 | 0·34 | 3·77 | 94·1 | 0·39 | 3·85 | 94·54 |
|  | 25 000 | 0·35 | 2·98 | 91·54 | 0·25 | 2·91 | 91·96 | 0·29 | 2·96 | 92·34 |
|  | 100 000 | 0·02 | 1·99 | 87·08 | -0·01 | 1·99 | 86·26 | 0·01 | 2 | 86·81 |
| Latino | 10 | 2·57 | 8·77 | 99·53 | -0·5 | 7·66 | 99·97 | 1·03 | 7·62 | 100 |
|  | 100 | 2·71 | 8·78 | 99·39 | 0 | 7·7 | 99·86 | 1·51 | 7·88 | 99·98 |
|  | 1000 | 1·12 | 7·45 | 97·66 | -0·32 | 7·21 | 98·3 | 0·51 | 7·89 | 98·74 |
|  | 3000 | 1·68 | 6·54 | 97·92 | 1 | 6·78 | 97·21 | 1·49 | 7·16 | 97·51 |
|  | 5000 | 0·94 | 5·83 | 96·97 | 0·48 | 5·95 | 96·3 | 0·79 | 6·2 | 96·96 |
|  | 10 000 | 0·17 | 4·84 | 96·37 | -0·12 | 4·87 | 95·16 | 0·04 | 5·06 | 95·81 |
|  | 25 000 | 0·18 | 3·79 | 94·86 | 0 | 4·01 | 92·84 | 0·14 | 3·98 | 94·46 |
|  | 100 000 | -0·09 | 2·78 | 89·78 | -0·13 | 2·82 | 88·04 | -0·11 | 2·85 | 89·33 |
| White | 10 | -1·07 | 5·19 | 98·63 | -0·76 | 5·95 | 99·44 | -1·54 | 5·72 | 98·93 |
|  | 100 | -1·09 | 5·13 | 98·24 | -0·87 | 6·29 | 99·38 | -1·51 | 5·66 | 98·48 |
|  | 1000 | -0·71 | 5·04 | 97·03 | -0·35 | 5·92 | 97·6 | -0·86 | 5·56 | 97·15 |
|  | 3000 | -0·1 | 4·08 | 94·97 | 0·15 | 4·56 | 95·98 | -0·13 | 4·32 | 95·38 |
|  | 5000 | -0·32 | 3·86 | 93·62 | -0·18 | 4·15 | 94·06 | -0·35 | 4·03 | 93·51 |
|  | 10 000 | 0·11 | 3·05 | 93·65 | 0·22 | 3·23 | 94·34 | 0·11 | 3·16 | 94·06 |
|  | 25 000 | 0·01 | 2·38 | 92·35 | -0·06 | 2·44 | 93·2 | 0 | 2·43 | 92·41 |
|  | 100 000 | -0·06 | 1·69 | 86·42 | -0·05 | 1·72 | 86·73 | -0·05 | 1·71 | 86·13 |

*“Across all racial–ethnic groups” indicates that errors and coverage were calculated across all racial–ethnic groups.

# 4 Supplemental Methods Figures

##

## 4.1 Analysis flow chart


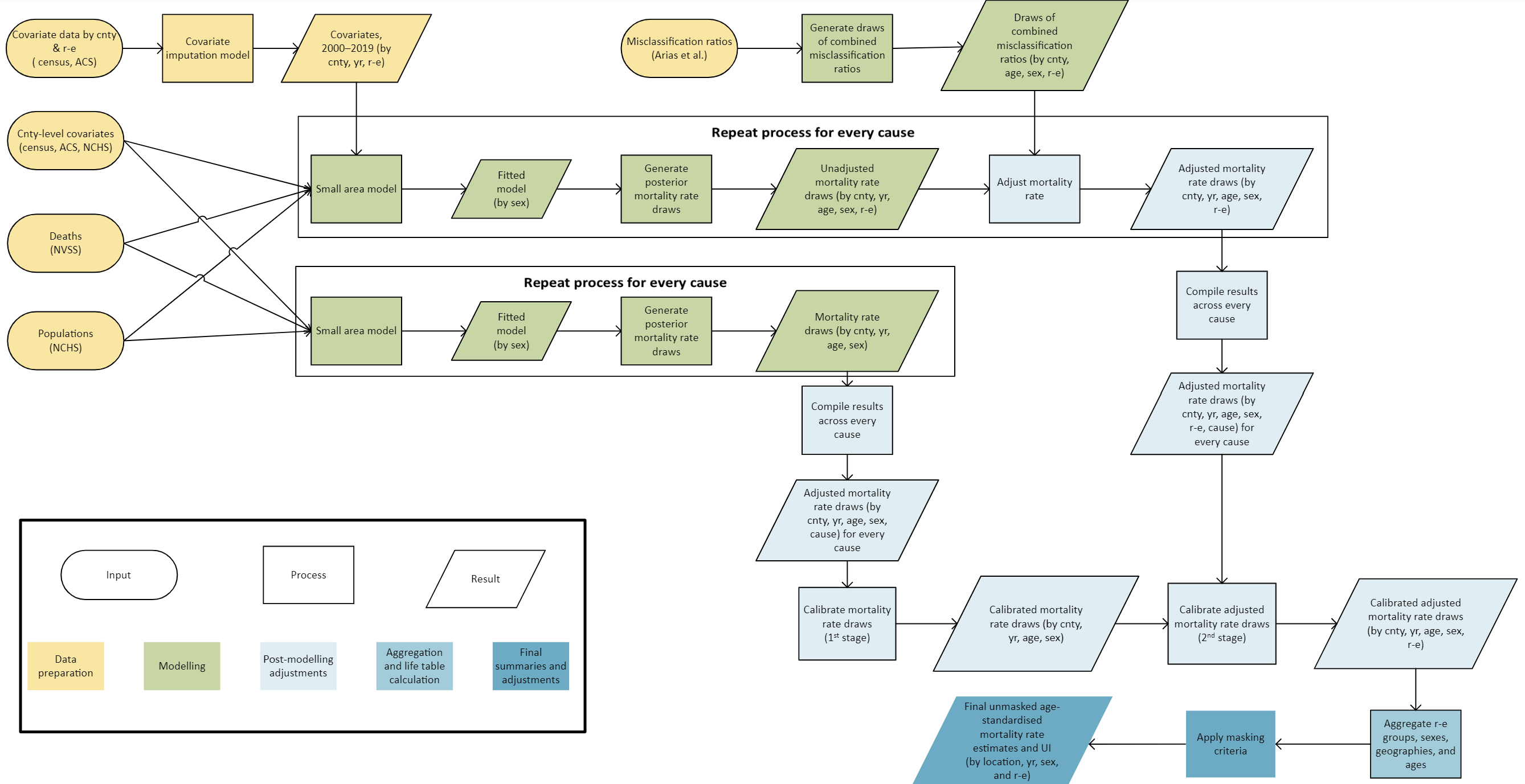


ACS = American Community Survey; cnty = county; NCHS = National Center for Health Statistics; NVSS = National Vital Statistics System; r-e = race–ethnicity; UI = uncertainty interval; yr = year.

## 4.2 Hyperprior sensitivity analysis results for selected Level 2 causes of death


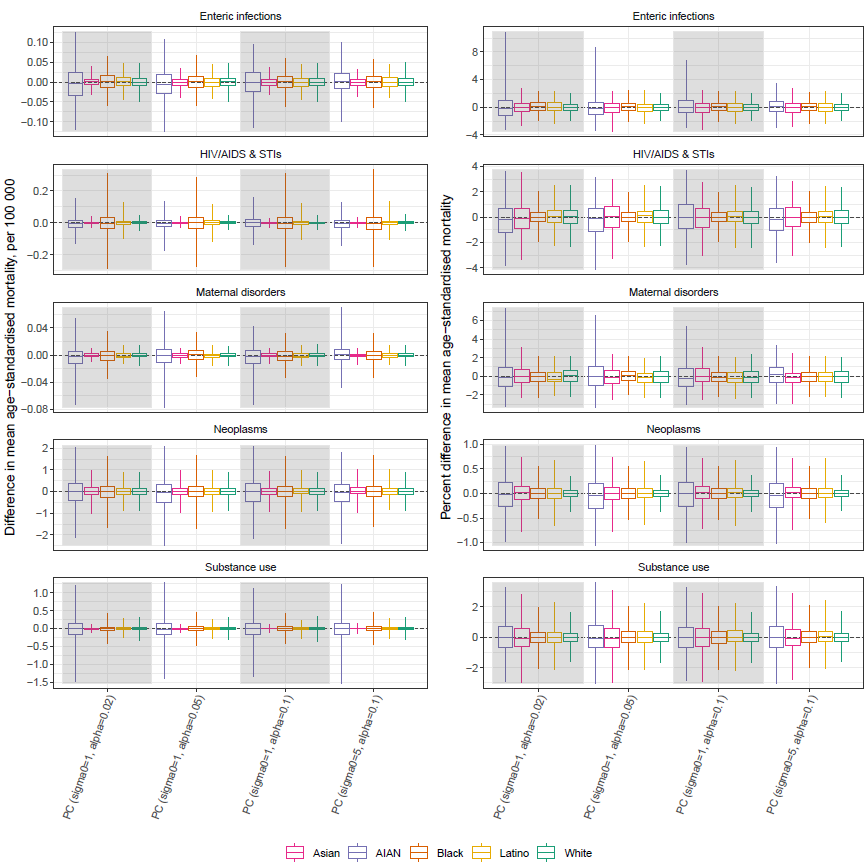


Population-weighted summary of differences (left: absolute differences; right: percent difference) in age-standardised mortality by county and racial–ethnic group for males and females combined in 2000, 2010, and 2019 compared with that of the model used in this paper (penalised complexity models, $\sigma_{0}=5, \alpha=0.05$). Boxes show the IQR, while the whiskers extend to 1^st^ and 99^th^ percentiles, weighted by the average population across all years in each county and racial–ethnic group combination. The data shown are for both masked and unmasked data. AIAN = non-Latino and non-Hispanic American Indian or Alaska Native.


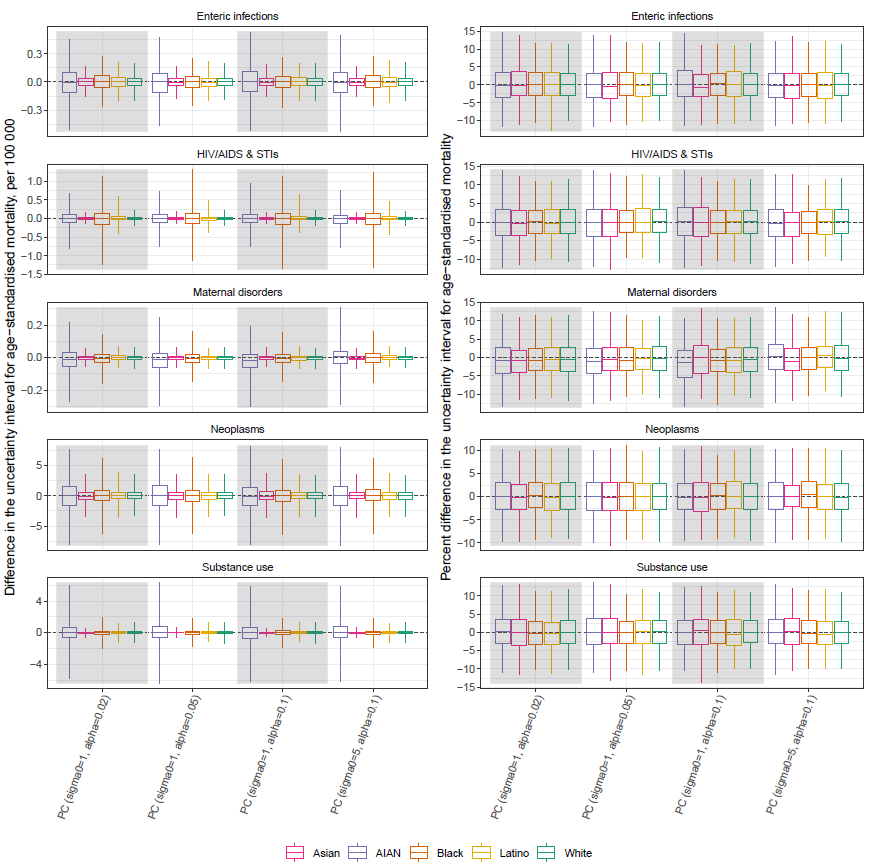


Population-weighted summary of the uncertainty intervals (UI) of differences (left: absolute differences in UI; right: percent difference in UI) in age-standardised mortality by county and racial–ethnic group for males and females combined in 2000, 2010, and 2019 compared with that of the model used in this paper (penalised complexity models, $\sigma_{0}=5, \alpha=0.05$). Boxes show the IQR, while the whiskers extend to 1^st^ and 99^th^ percentiles, weighted by the average population across all years in each county and racial–ethnic group combination. The data shown are for both masked and unmasked data. AIAN = non-Latino and non-Hispanic American Indian or Alaska Native.

## 4.3 All-cause mortality model validation results


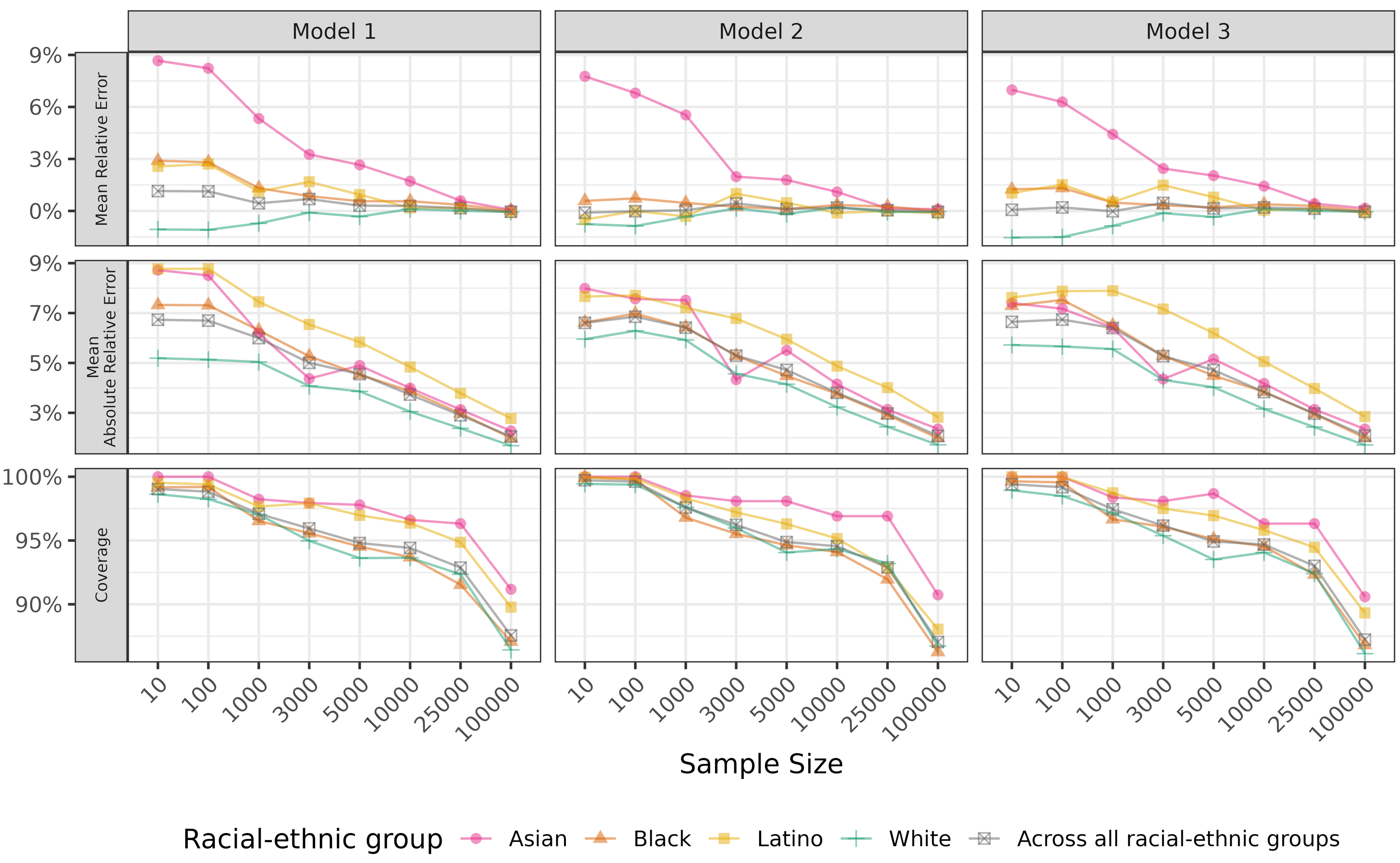


*“Across all racial–ethnic groups” means that errors and coverage were calculated across all racial–ethnic groups.

## 4.4 Impact of misclassification adjustment on national age-standardised all-cause mortality


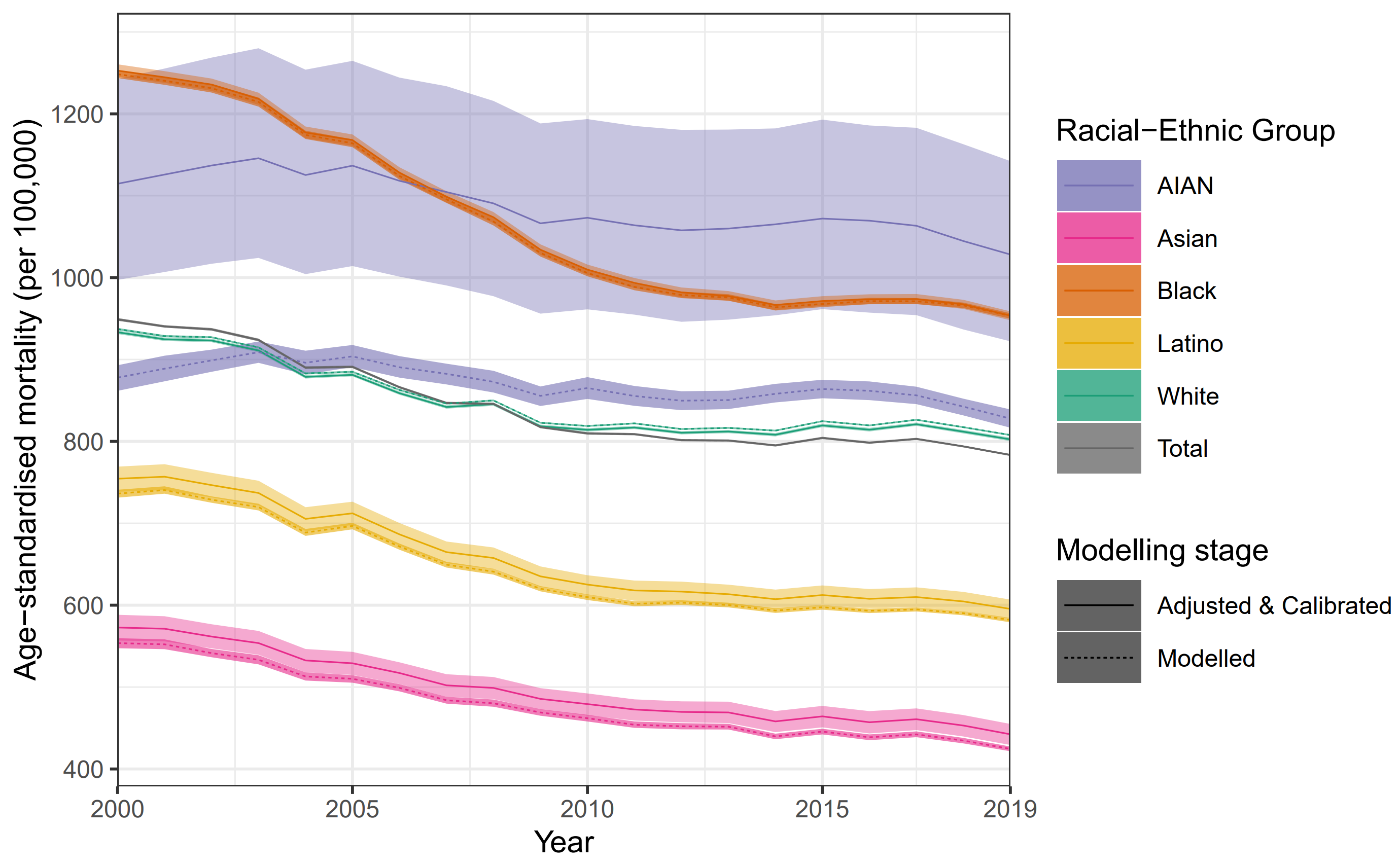


National age-standardised mortality estimates before adjustment for misclassification (“Modelled”) and after adjustment and calibration (“Adjusted & Calibrated”)—ie, the final estimates.

## 4.5 Impact of misclassification adjustment on county age-standardised all-cause mortality estimates


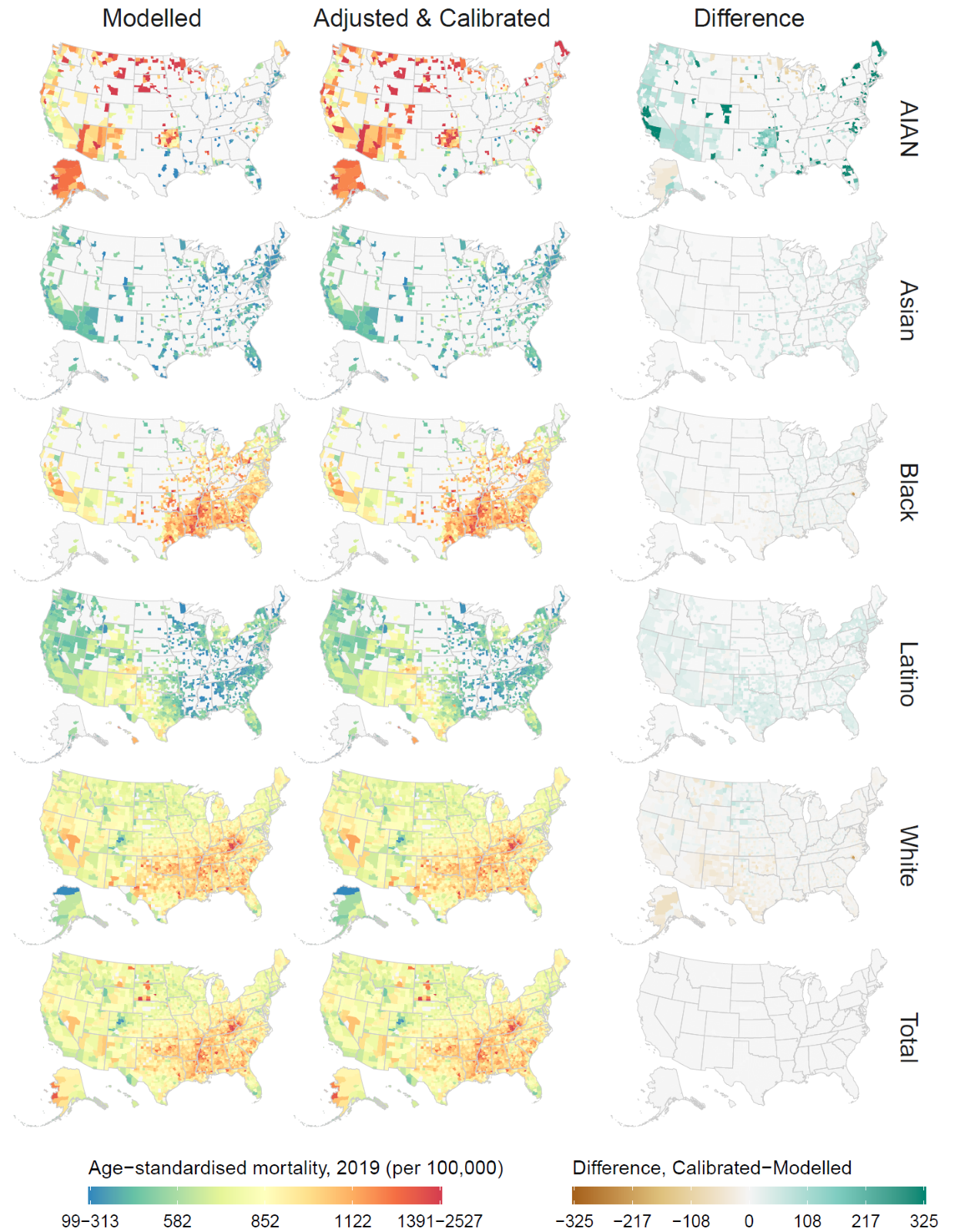


County age-standardised all-cause mortality estimates before adjustment for misclassification (“Modelled”) and after adjustment and calibration (“Adjusted & Calibrated”)—ie, the final estimates. Estimates have been masked for county and racial–ethnic groups with a mean annual population fewer than 1000 people because model performance declined notably below this threshold. AIAN = non-Latino and non-Hispanic American Indian or Alaska Native.

# 5 Supplemental Results Tables

## eTable 1: Change in absolute disparities in stomach cancer mortality among the AIAN, Asian, Black, and Latino populations compared with the White population in the same county, 2000 to 2019

| **Racial–ethnic group** | **Measure** | **Number of counties** | **Disparity < 0 in 2000, more negative in 2019** | **Disparity > 0 in 2000, bigger in 2019** | **Disparity < 0 in 2000, > 0 in 2019** | **Disparity > 0 in 2000, < 0 in 2019** | **Disparity < 0 in 2000, less negative in 2019** | **Disparity > 0 in 2000, less positive in 2019** |
| --- | --- | --- | --- | --- | --- | --- | --- | --- |
| AIAN | Total | 465 | 21 (4·5%) | 124 (26·7%) | 19 (4·1%) | 17 (3·7%) | 50 (10·8%) | 234 (50·3%) |
|  | Significant |  | 0 (0%) | 3 (0·6%) | 0 (0%) | 0 (0%) | 5 (1·1%) | 27 (5·8%) |
| Asian | Total | 667 | 0 (0%) | 61 (9·1%) | 4 (0·6%) | 2 (0·3%) | 2 (0·3%) | 598 (89·7%) |
|  | Significant |  | 0 (0%) | 0 (0%) | 0 (0%) | 0 (0%) | 0 (0%) | 266 (39·9%) |
| Black | Total | 1486 | 0 (0%) | 7 (0·5%) | 1 (0·1%) | 1 (0·1%) | 1 (0·1%) | 1476 (99·3%) |
|  | Significant |  | 0 (0%) | 0 (0%) | 0 (0%) | 0 (0%) | 0 (0%) | 1164 (78·3%) |
| Latino | Total | 1469 | 12 (0·8%) | 268 (18·2%) | 185 (12·6%) | 20 (1·4%) | 142 (9·7%) | 842 (57·3%) |
|  | Significant |  | 0 (0%) | 1 (0·1%) | 0 (0%) | 0 (0%) | 6 (0·4%) | 232 (15·8%) |
| Total | Total | 3051 | 64 (2·1%) | 1419 (46·5%) | 21 (0·7%) | 68 (2·2%) | 6 (0·2%) | 1473 (48·3%) |
|  | Significant |  | 0 (0%) | 522 (17·1%) | 0 (0%) | 0 (0%) | 0 (0%) | 751 (24·6%) |

Number of counties in each category of change (# counties [percentage]). AIAN = non-Latino and non-Hispanic American Indian or Alaska Native.

## eTable 2: Change in relative disparities in stomach cancer mortality among the AIAN, Asian, Black, and Latino populations compared with the White population in the same county, 2000 to 2019

| **Racial–ethnic group** | **Measure** | **Number of counties** | **Disparity < 1 in 2000, smaller in 2019** | **Disparity > 1 in 2000, larger in 2019** | **Disparity < 1 in 2000, > 1 in 2019** | **Disparity > 1 in 2000, < 1 in 2019** | **Disparity < 1 in 2000, larger in 2019** | **Disparity > 1 in 2000, smaller in 2019** |
| --- | --- | --- | --- | --- | --- | --- | --- | --- |
| AIAN | Total | 465 | 44 (9·5%) | 279 (60%) | 19 (4·1%) | 17 (3·7%) | 27 (5·8%) | 79 (17%) |
|  | Significant |  | 5 (1·1%) | 31 (6·7%) | 0 (0%) | 0 (0%) | 2 (0·4%) | 2 (0·4%) |
| Asian | Total | 667 | 1 (0·1%) | 201 (30·1%) | 4 (0·6%) | 2 (0·3%) | 1 (0·1%) | 458 (68·7%) |
|  | Significant |  | 0 (0%) | 4 (0·6%) | 0 (0%) | 0 (0%) | 0 (0%) | 62 (9·3%) |
| Black | Total | 1486 | 1 (0·1%) | 156 (10·5%) | 1 (0·1%) | 1 (0·1%) | 0 (0%) | 1327 (89·3%) |
|  | Significant |  | 0 (0%) | 2 (0·1%) | 0 (0%) | 0 (0%) | 0 (0%) | 251 (16·9%) |
| Latino | Total | 1469 | 28 (1·9%) | 669 (45·5%) | 185 (12·6%) | 20 (1·4%) | 126 (8·6%) | 441 (30%) |
|  | Significant |  | 0 (0%) | 28 (1·9%) | 0 (0%) | 0 (0%) | 0 (0%) | 16 (1·1%) |
| Total | Total | 3051 | 67 (2·2%) | 1906 (62·5%) | 21 (0·7%) | 68 (2·2%) | 3 (0·1%) | 986 (32·3%) |
|  | Significant |  | 0 (0%) | 1075 (35·2%) | 0 (0%) | 0 (0%) | 0 (0%) | 108 (3·5%) |

Number of counties in each category of change (# counties [percentage]). AIAN = non-Latino and non-Hispanic American Indian or Alaska Native.

# 6 Supplemental Results Figures

eFigure 1: Age-standardised mortality rate, stomach cancer, 2000


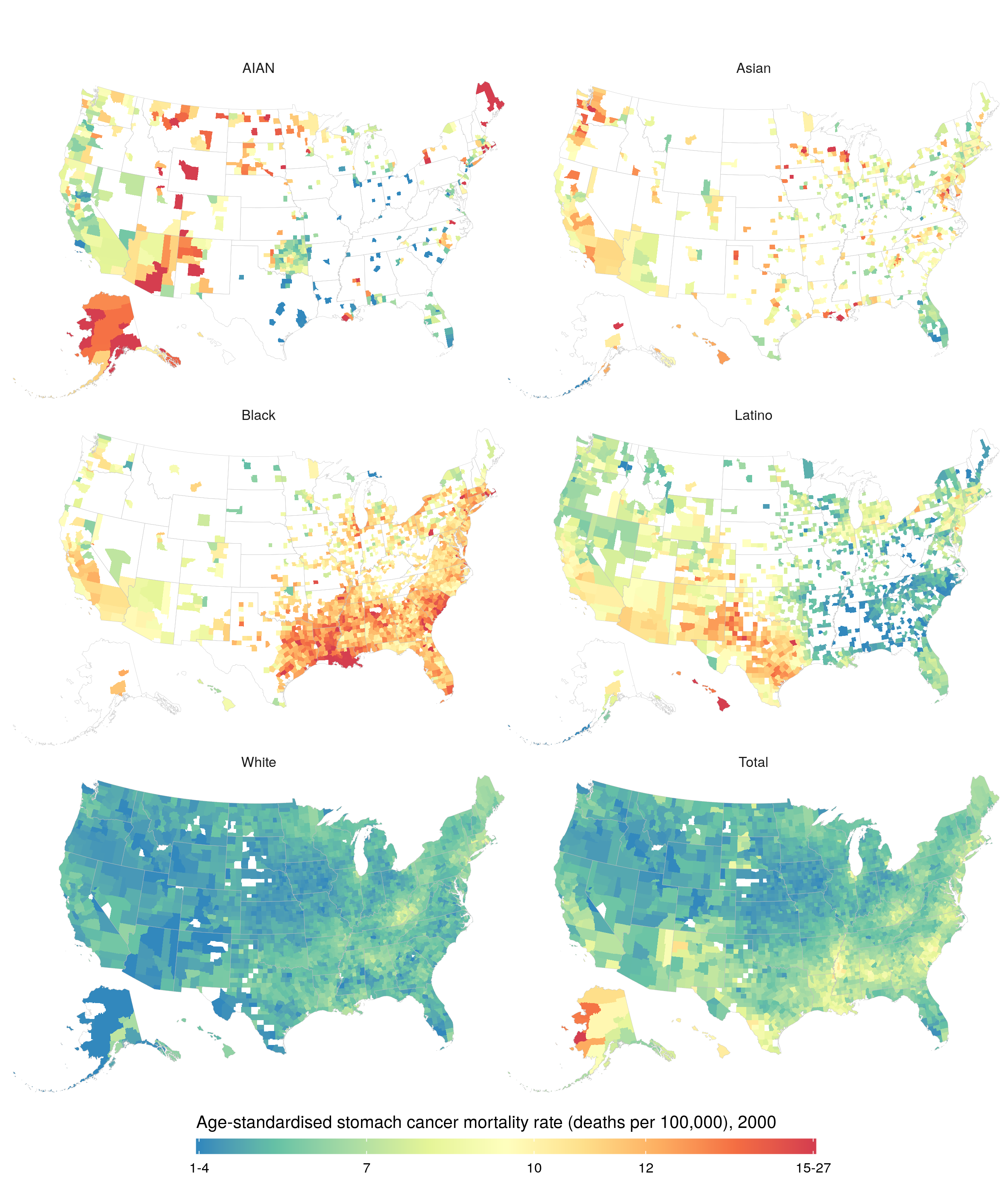
 Note: estimates are not shown for county-racial-ethnic group combinations with an average annual population less than 1,000 people.

eFigure 2: Age-standardised mortality rate ratio compared to the White population in the same county, stomach cancer, 2000


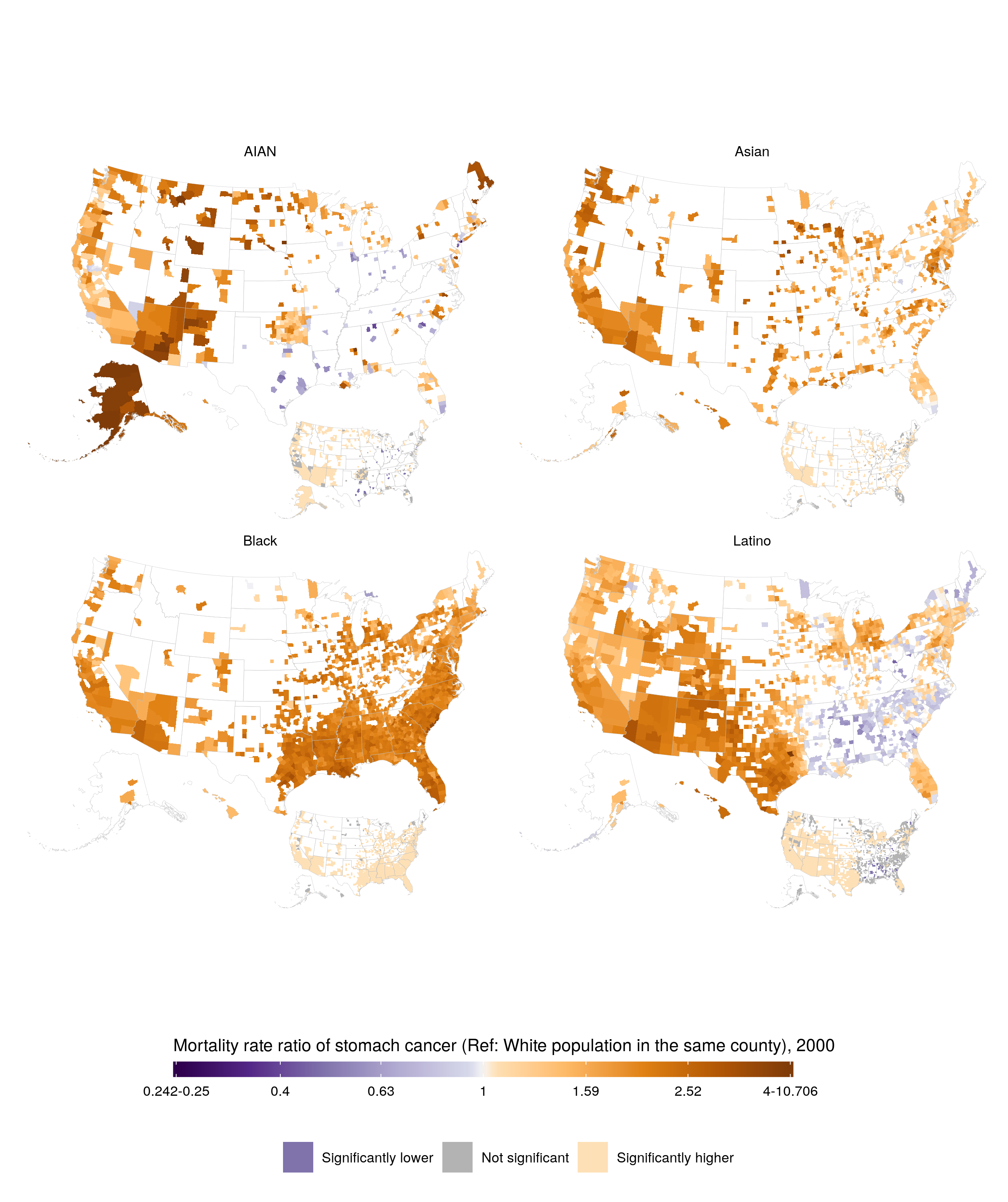
 Note: estimates are not shown for county-racial-ethnic group combinations with an average annual population less than 1,000 people.

eFigure 3: Age-standardised absolute difference in mortality compared to the White population in the same county, stomach cancer, 2000


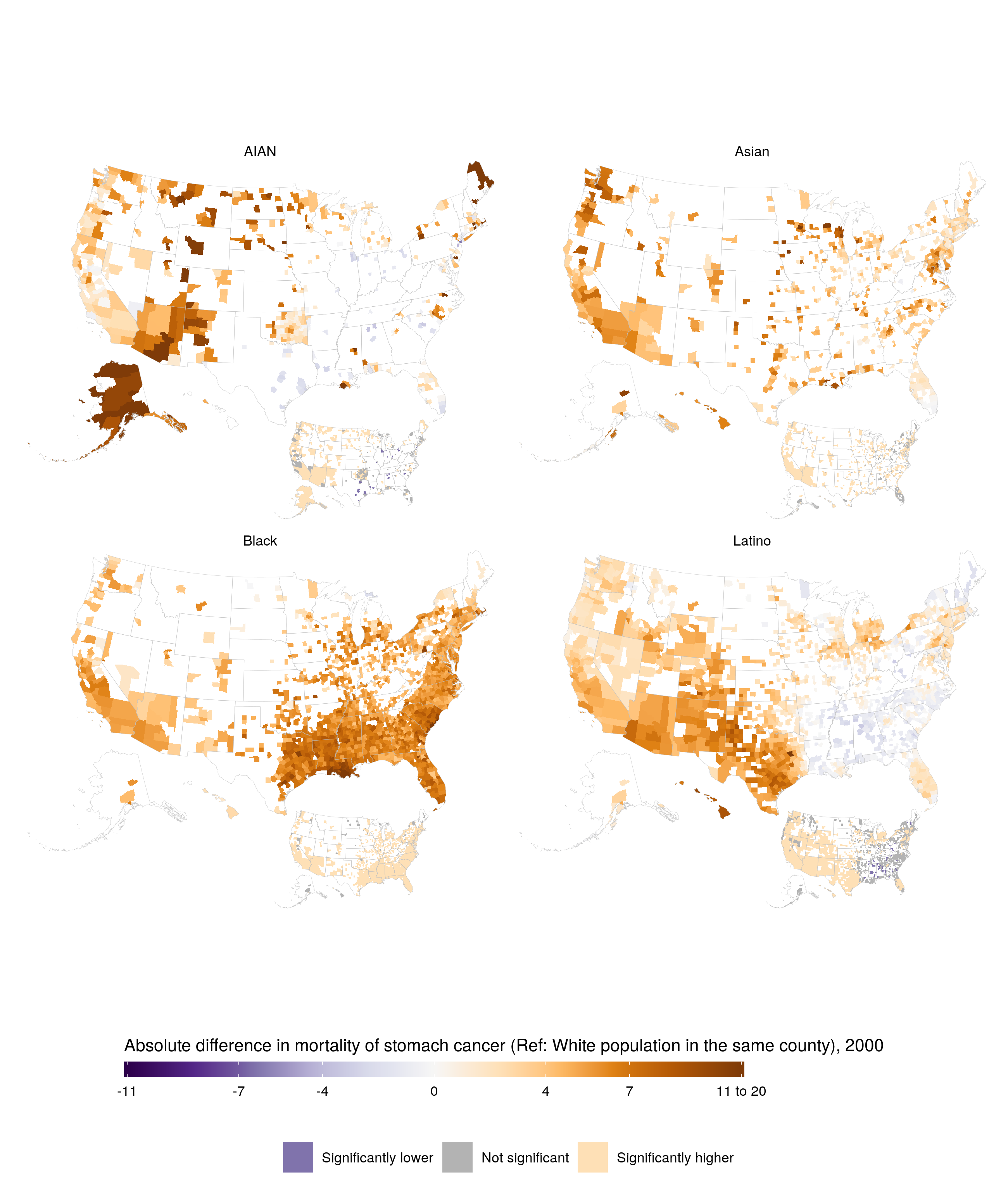
 Note: estimates are not shown for county-racial-ethnic group combinations with an average annual population less than 1,000 people.

eFigure 4: Age-standardised absolute difference in mortality compared to the White population in the same county, stomach cancer, 2019


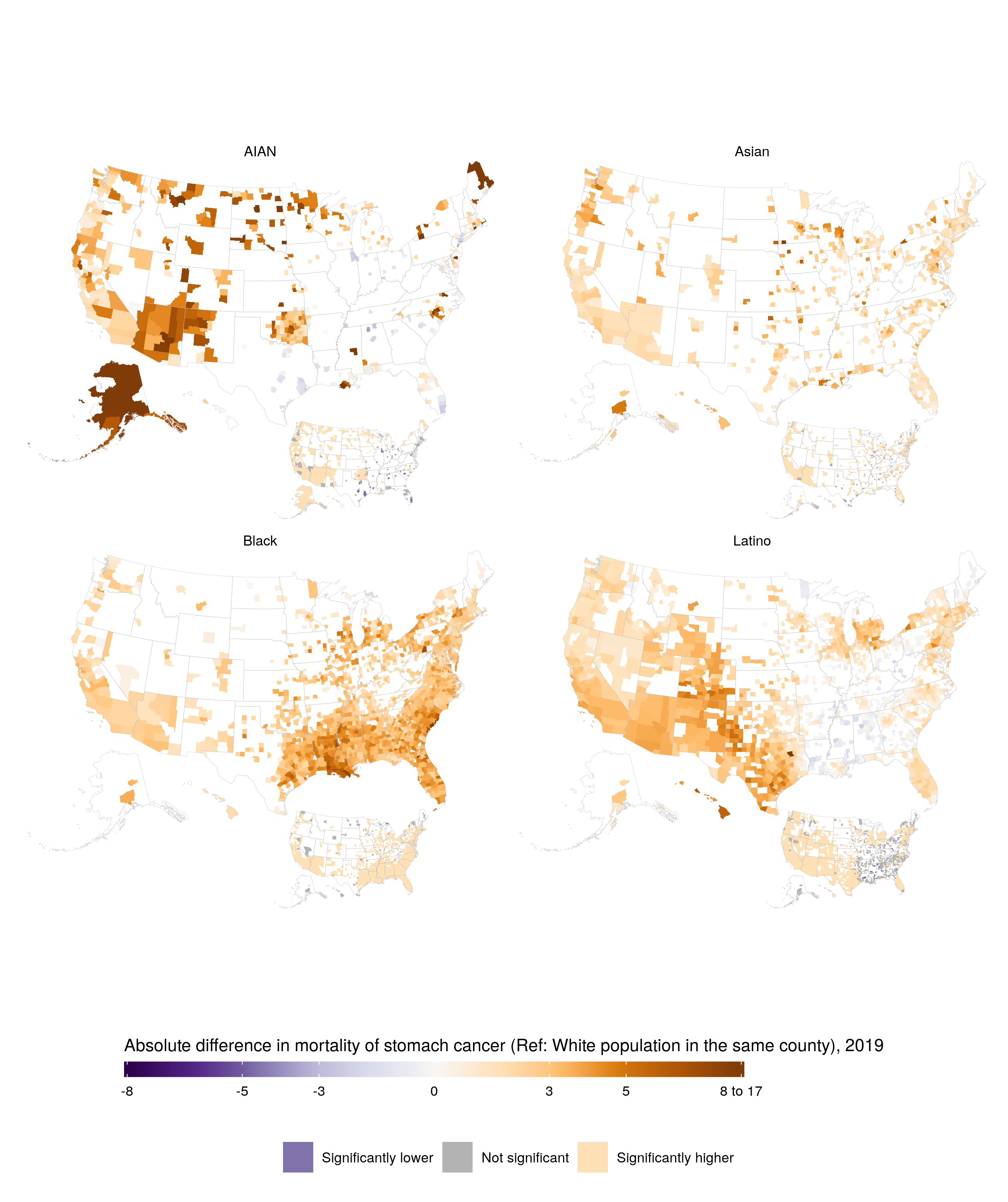
 Note: estimates are not shown for county-racial-ethnic group combinations with an average annual population less than 1,000 people.

eFigure 5: Percent change in age-standardised mortality rate, stomach cancer, 2000-2010


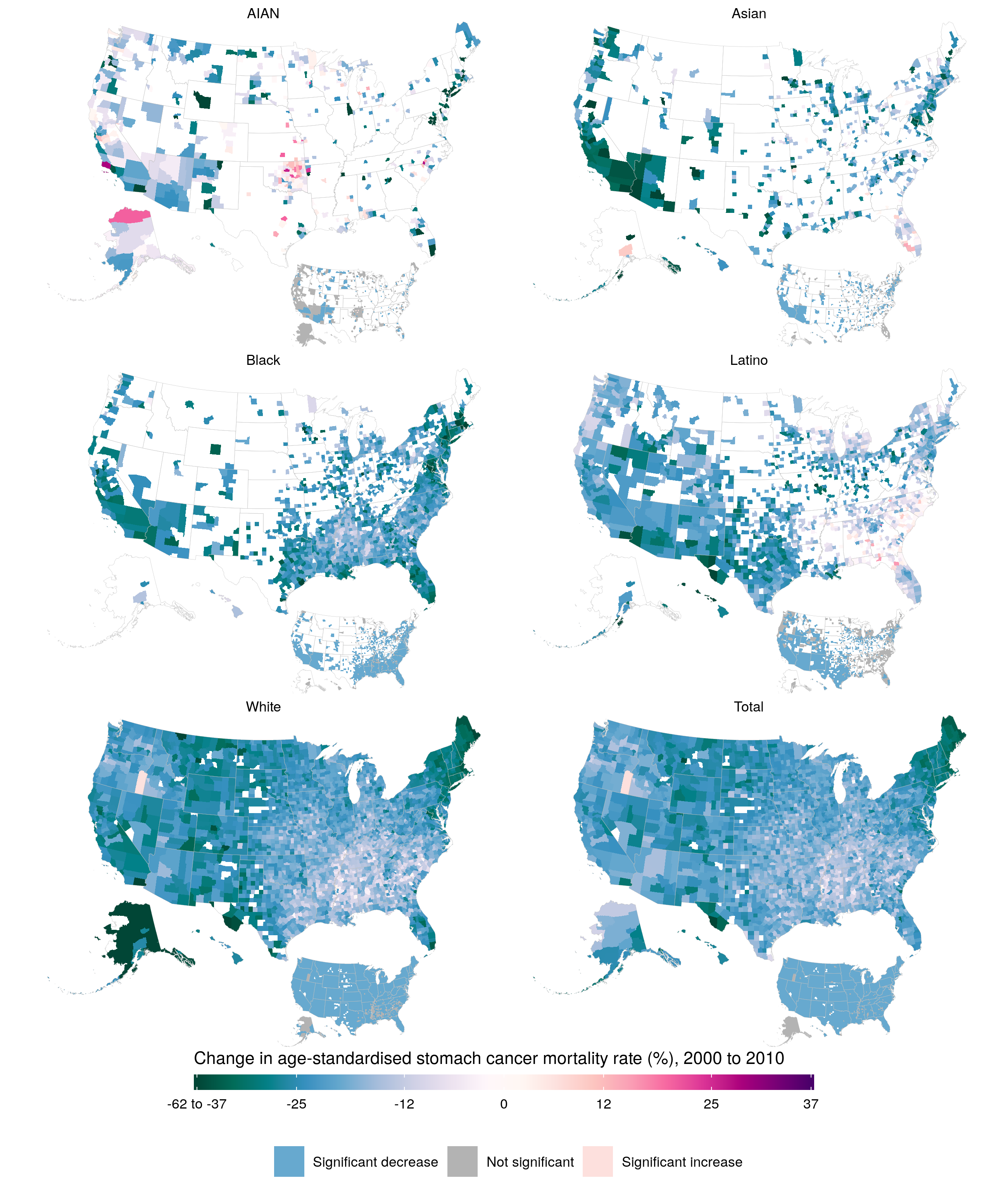
 Note: estimates are not shown for county-racial-ethnic group combinations with an average annual population less than 1,000 people.

eFigure 6: Absolute change in age-standardised mortality rate, stomach cancer, 2000-2010


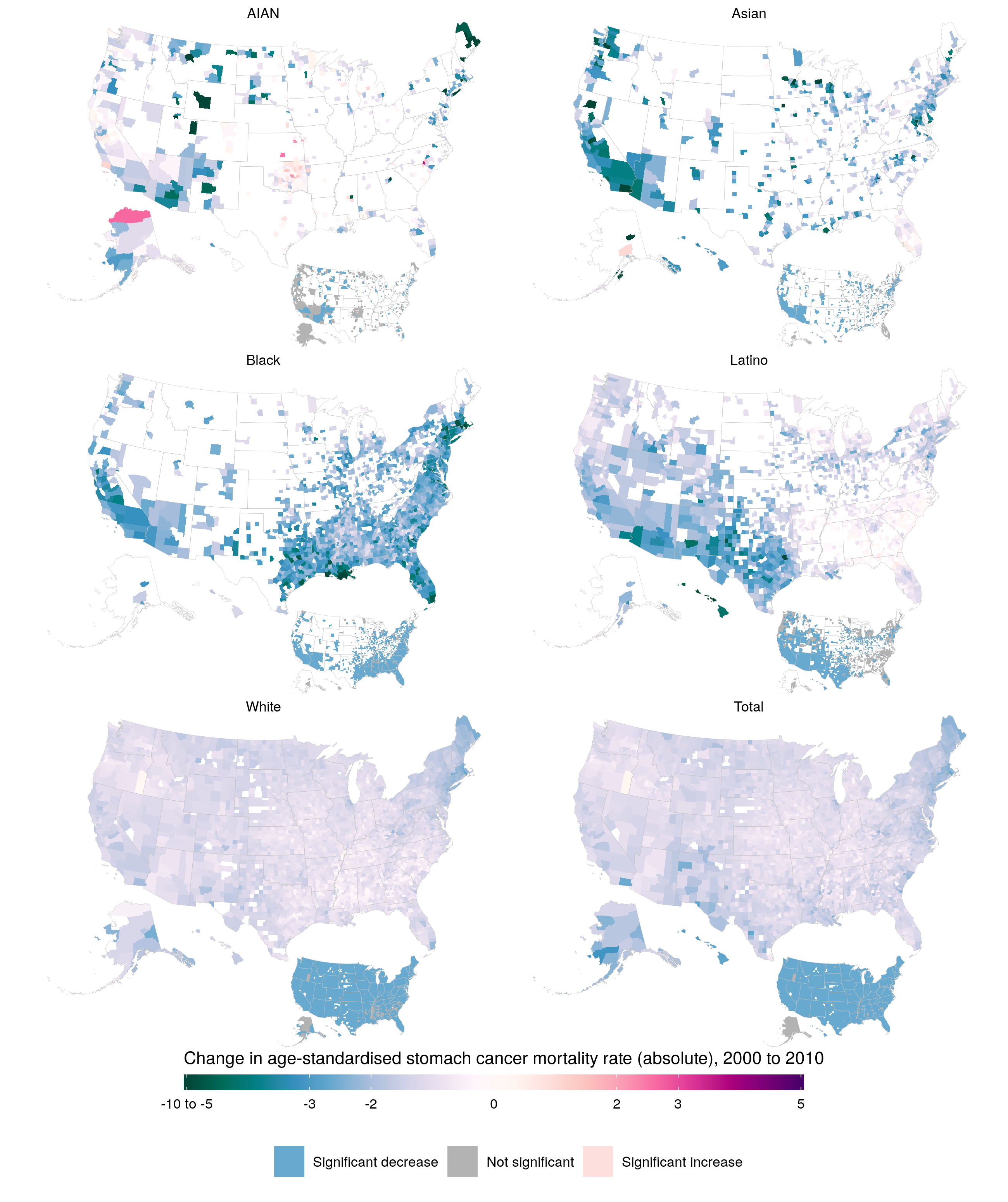
 Note: estimates are not shown for county-racial-ethnic group combinations with an average annual population less than 1,000 people.

eFigure 7: Percent change in age-standardised mortality rate, stomach cancer, 2010-2019


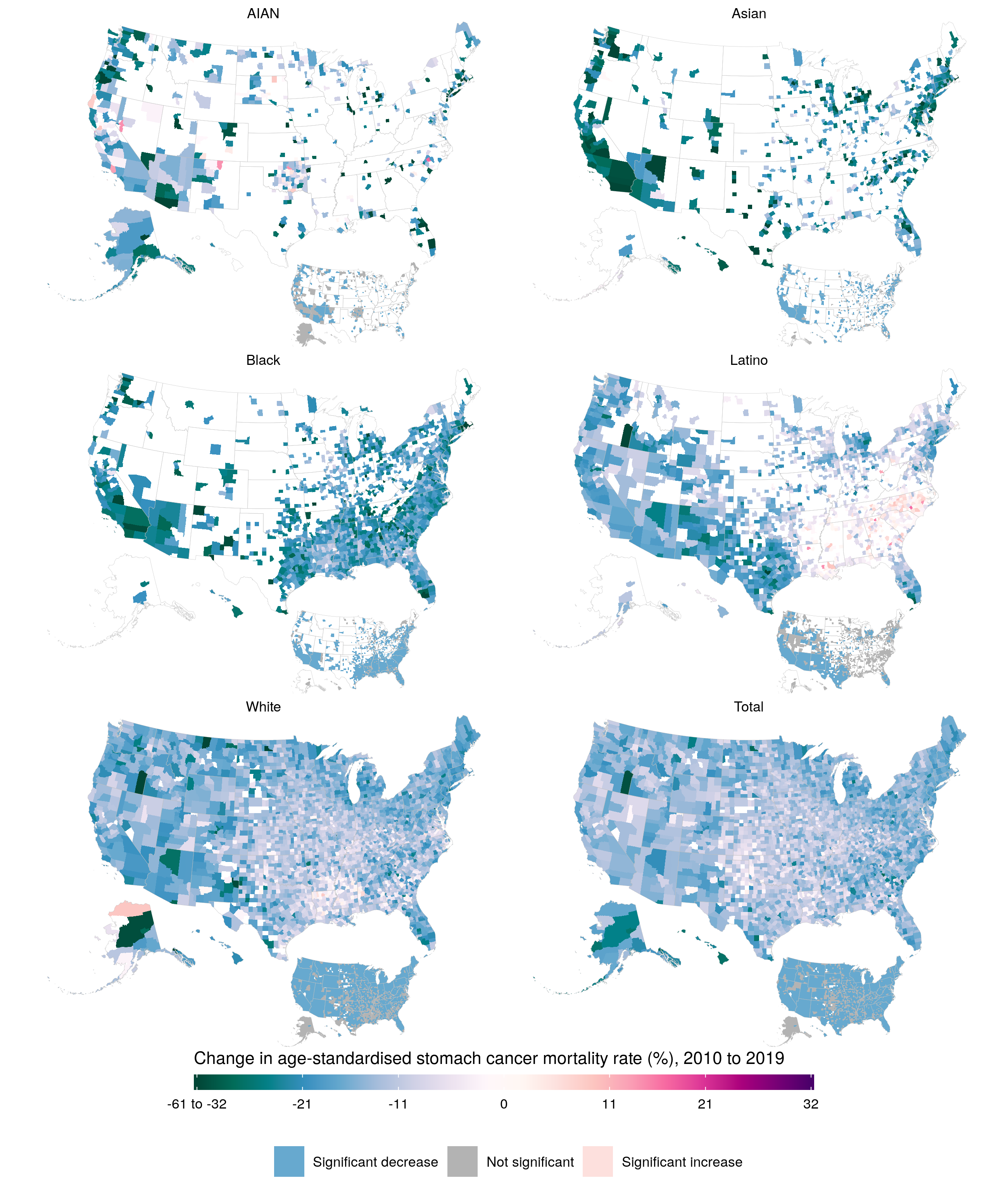
 Note: estimates are not shown for county-racial-ethnic group combinations with an average annual population less than 1,000 people.

eFigure 8: Absolute change in age-standardised mortality rate, stomach cancer, 2010-2019


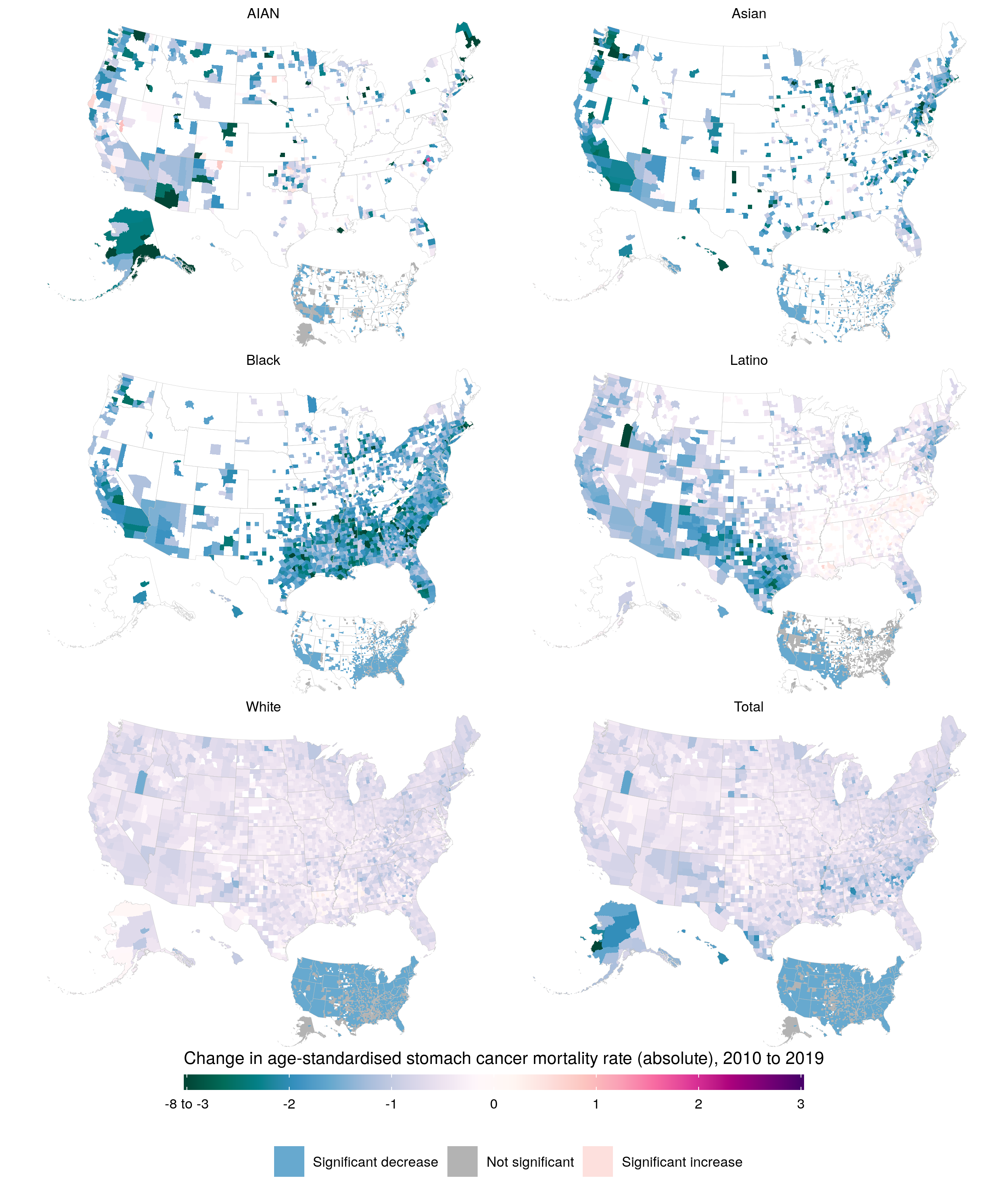
 Note: estimates are not shown for county-racial-ethnic group combinations with an average annual population less than 1,000 people.

eFigure 9: Absolute change in age-standardised mortality rate, stomach cancer, 2000-2019


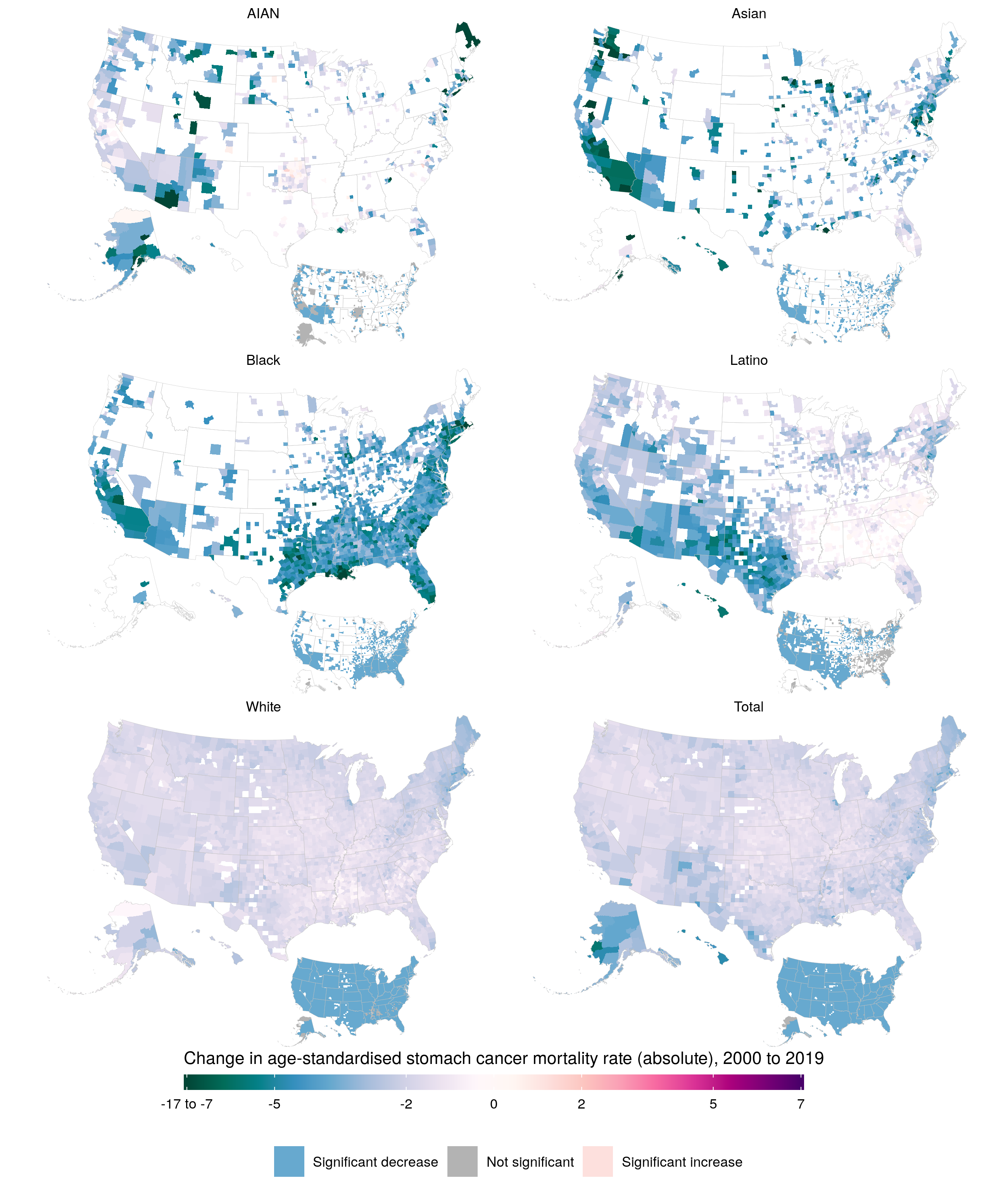
 Note: estimates are not shown for county-racial-ethnic group combinations with an average annual population less than 1,000 people.

eFigure 10: National-level age-standardised mortality rate, stomach cancer, 2019, Males


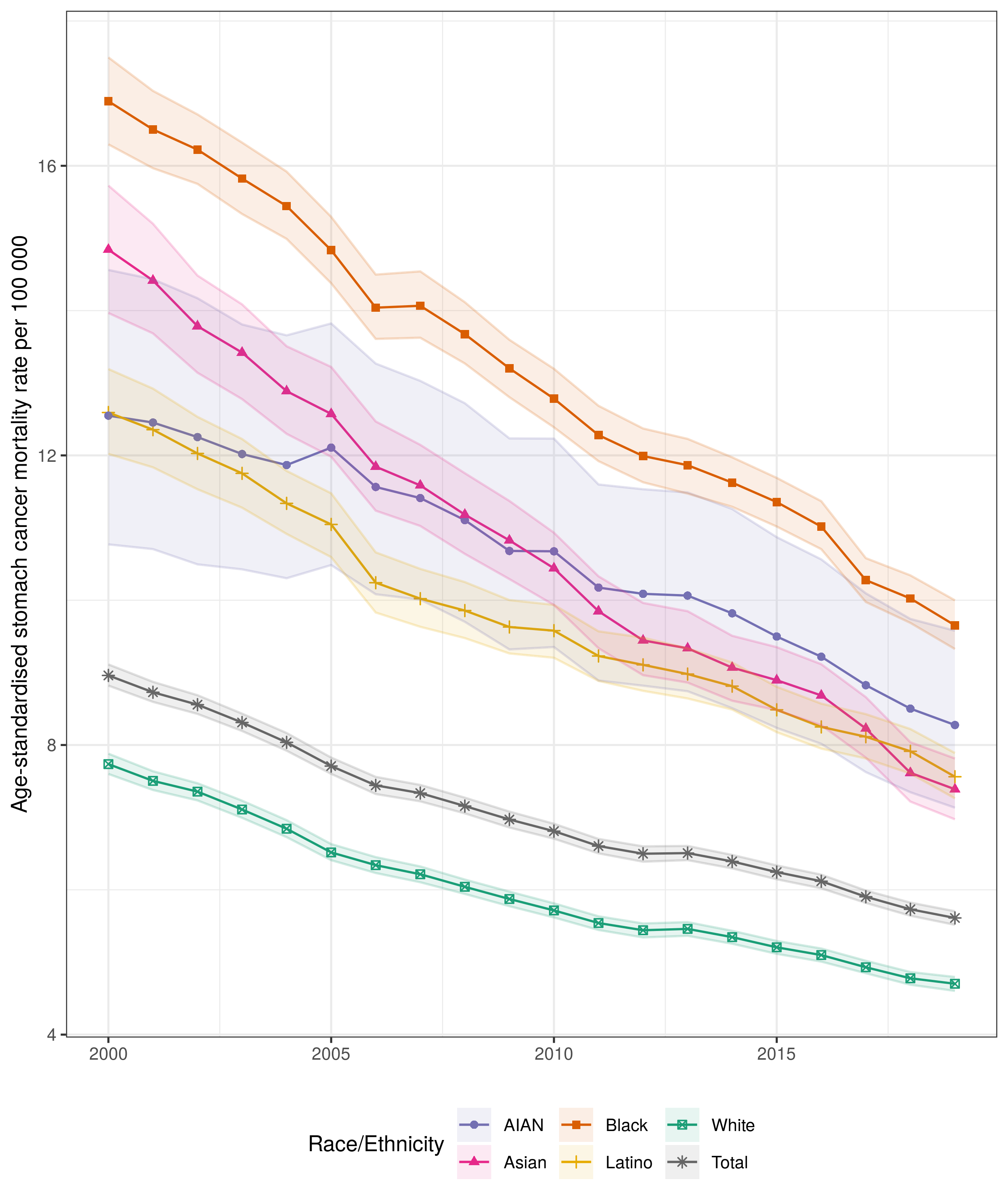


eFigure 11: Age-standardised mortality rate, stomach cancer, 2019, Males


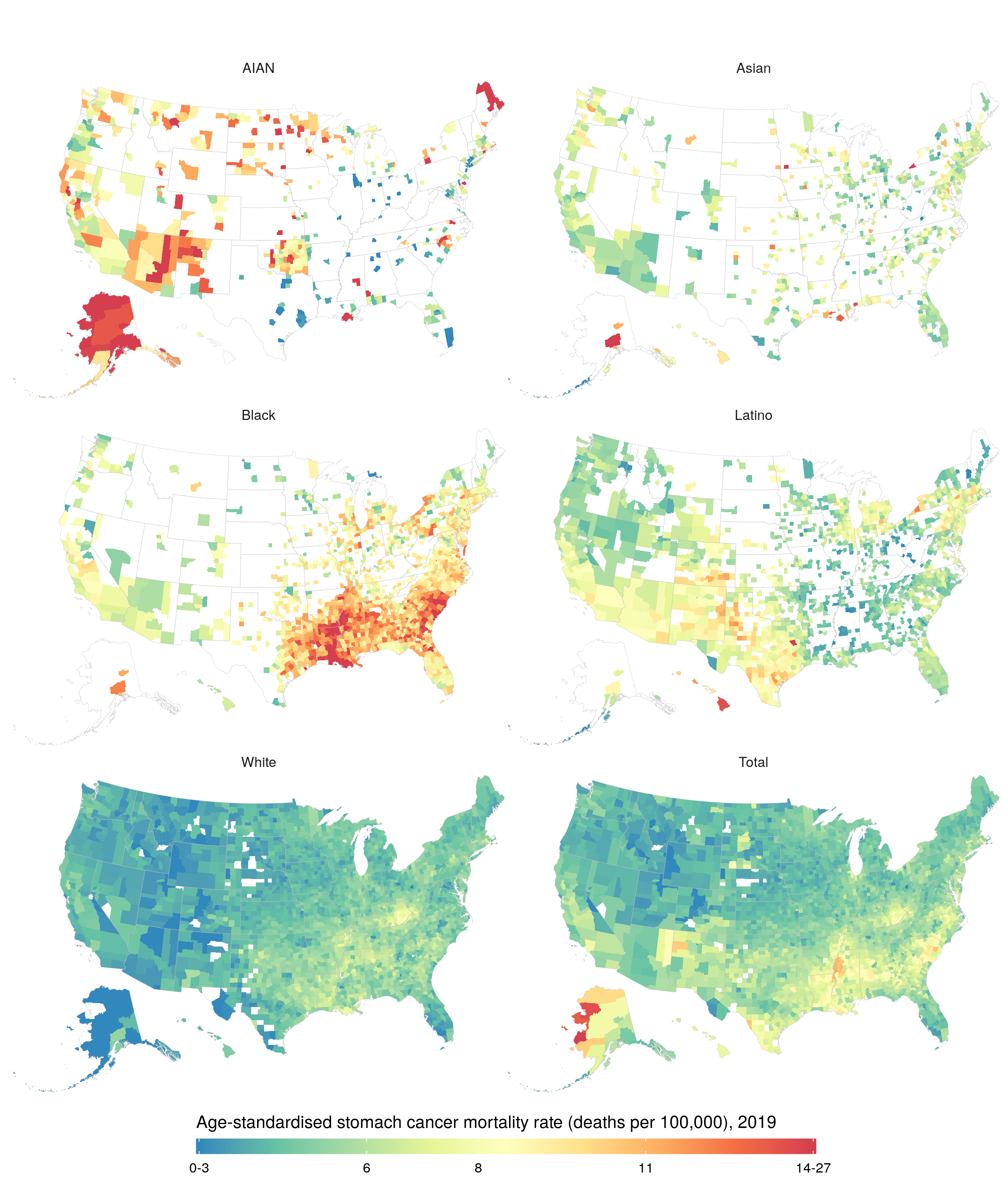
 Note: estimates are not shown for county-racial-ethnic group combinations with an average annual population less than 1,000 people.

eFigure 12: Age-standardised mortality rate ratio compared to the White population in the same county, stomach cancer, 2019, Males


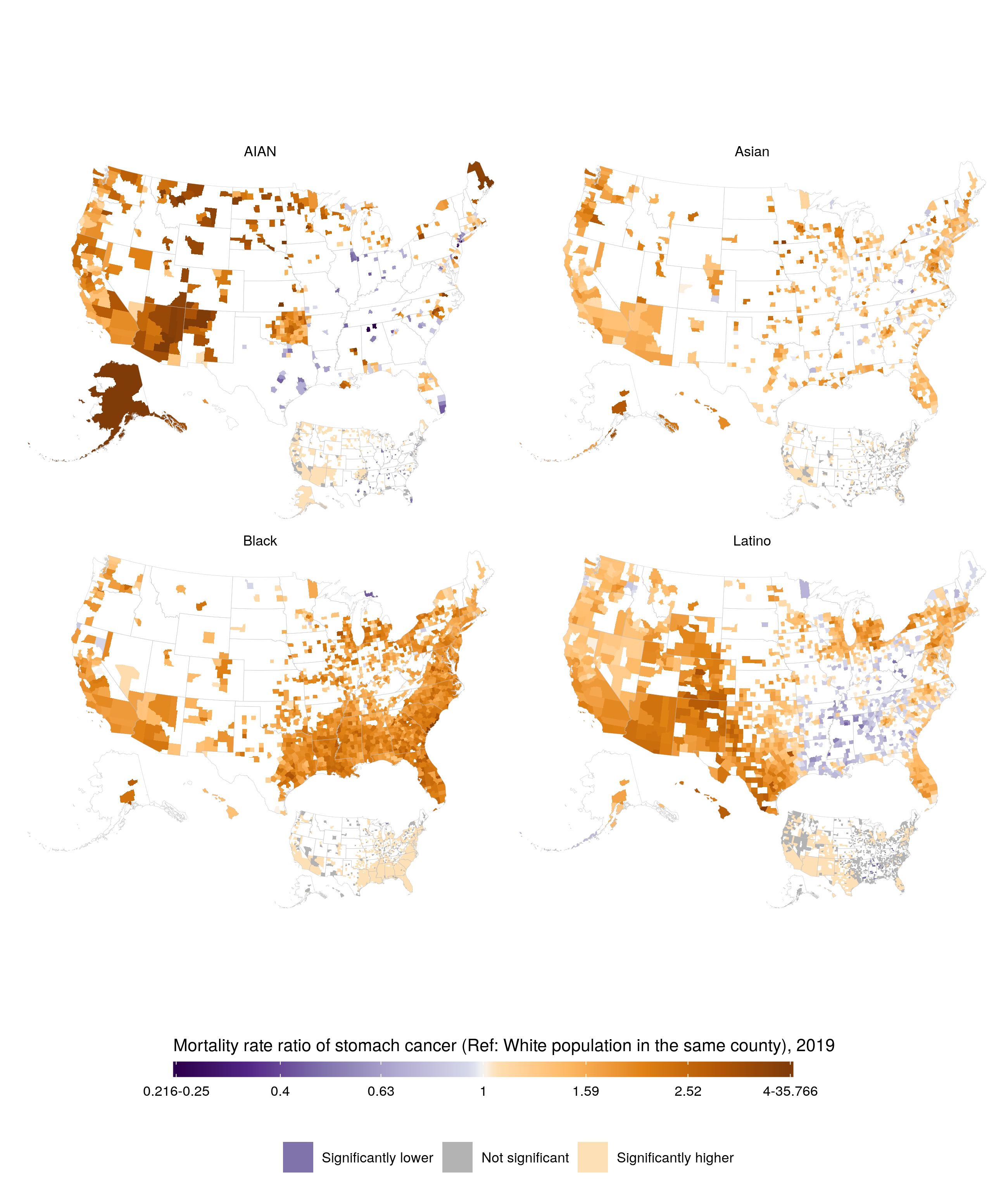
 Note: estimates are not shown for county-racial-ethnic group combinations with an average annual population less than 1,000 people.

eFigure 13: National-level age-standardised mortality rate, stomach cancer, 2019, Females


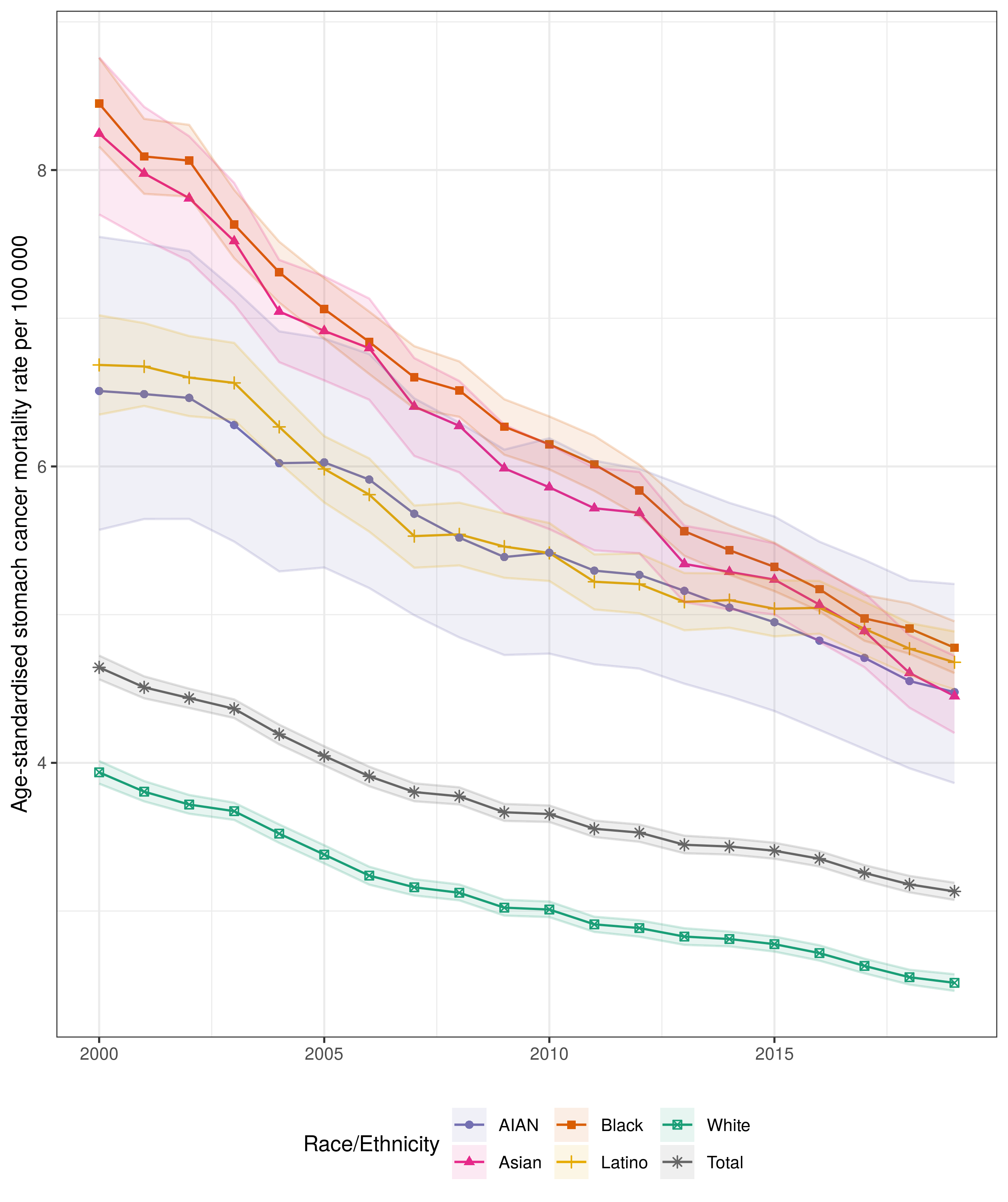


eFigure 14: Age-standardised mortality rate, stomach cancer, 2019, Females


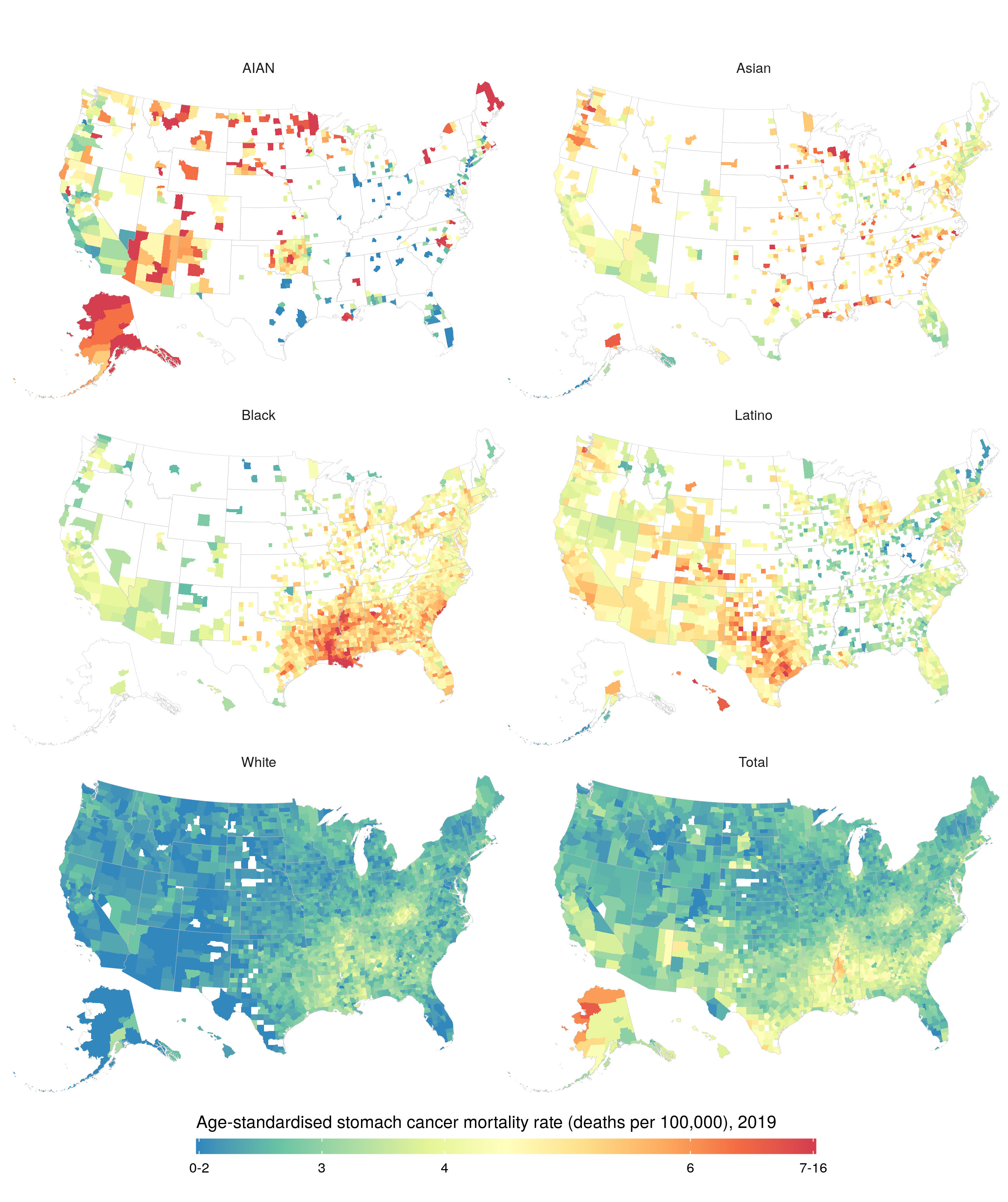
 Note: estimates are not shown for county-racial-ethnic group combinations with an average annual population less than 1,000 people.

eFigure 15: Age-standardised mortality rate ratio compared to the White population in the same county, stomach cancer, 2019, Females


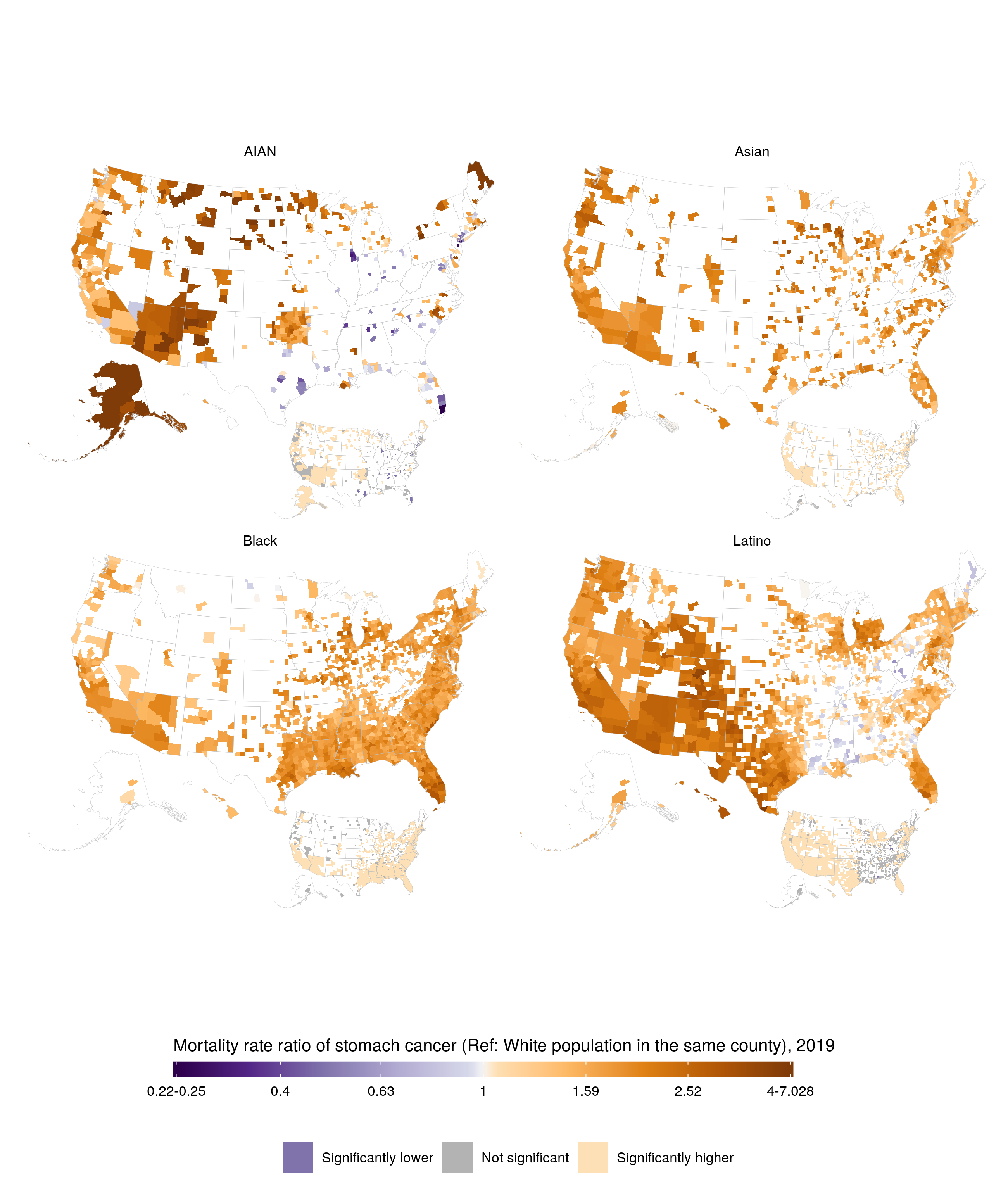
 Note: estimates are not shown for county-racial-ethnic group combinations with an average annual population less than 1,000 people.
